# Supplementary material for: Loss of Elp3 blocks intestinal tuft cell differentiation via an mTORC1-Atf4 axis
Source: EMBO J. 2024 Jul 31;43(18):6. doi: 10.1038/s44318-024-00184-4 (PMC11405396; doi:10.1038/s44318-024-00184-4)
Supplement: Supplementary file 1 — Table EV1 [file 44318_2024_184_MOESM1_ESM.docx]

**Table EV1 : Table Cell Populations**

| Sample | WT.U047 | WT.U049 | KO.U048 | KO.U050 |
| --- | --- | --- | --- | --- |
| **Genotype** | **WT** | | **KO Elp3** | |
| Enterocyte progenitors | 151 | 209 | 164 | 289 |
| Enteroendocrine cells | 39 | 27 | 29 | 35 |
| Goblet cells | 62 | 50 | 49 | 60 |
| Immature Enterocytes | 289 | 350 | 351 | 452 |
| Immature Goblet cells | 112 | 199 | 106 | 176 |
| Immature Tuft cells | 22 | 46 | 6 | 22 |
| Mature Enterocytes | 883 | 761 | 1045 | 915 |
| Paneth cells | 89 | 85 | 106 | 93 |
| Stem cells | 51 | 98 | 38 | 119 |
| Transit Amplifying cells | 117 | 144 | 124 | 240 |
| Tuft cells | 47 | 53 | 16 | 48 |
| TOTAL | 1862 | 2022 | 2034 | 2449 |
|  |  |  |  |  |
| Proportion (ratio on total number) |  |  |  |  |
| Enterocyte progenitors | 0,081095596 | 0,103363007 | 0,080629302 | 0,11800735 |
| Enteroendocrine cells | 0,02094522 | 0,013353116 | 0,01425762 | 0,014291548 |
| Goblet cells | 0,03329753 | 0,024727992 | 0,024090462 | 0,024499796 |
| Immature Enterocytes | 0,155209452 | 0,173095945 | 0,172566372 | 0,184565129 |
| Immature Goblet cells | 0,060150376 | 0,098417409 | 0,052114061 | 0,071866068 |
| Immature Tuft cells | 0,011815252 | 0,022749753 | 0,002949853 | 0,008983258 |
| Mature Enterocytes | 0,474221267 | 0,37636004 | 0,513765978 | 0,373621886 |
| Paneth cells | 0,047798067 | 0,042037587 | 0,052114061 | 0,037974684 |
| Stem cells | 0,027389903 | 0,048466864 | 0,018682399 | 0,048591262 |
| Transit Amplifying cells | 0,062835661 | 0,071216617 | 0,060963618 | 0,097999183 |
| Tuft cells | 0,025241676 | 0,026211672 | 0,007866273 | 0,019599837 |
|  |  |  |  |  |
|  |  |  |  |  |
