# Supplementary material for: Loss of Elp3 blocks intestinal tuft cell differentiation via an mTORC1-Atf4 axis
Source: EMBO J. 2024 Jul 31;43(18):6. doi: 10.1038/s44318-024-00184-4 (PMC11405396; doi:10.1038/s44318-024-00184-4)
Supplement: Supplementary file 2 — Table EV2 [file 44318_2024_184_MOESM2_ESM.docx]

**Table EV2 : Reagents or resources**

|  |  |  | |
| --- | --- | --- | --- |
| **REAGENT or RESOURCE** | **SOURCE** | **IDENTIFIER** |  |
|  |  |  | |
| **Antibodies** |  |  | |
|  |  |  | |
| Anti-Dclk1 | Abcam | Cat# ab31704, RRID:AB_873537 | |
| Anti-FFAR3/GPR41 | Abcam | Cat# ab236654, RRID:AB_2892609 | |
| Anti-IL-13Rα1 | Abcam | Cat# ab79277, RRID:AB_1640587 | |
| Anti-pIRE1α (Ser724) | Abcam | Cat# ab124945, RRID:AB_11001365 | |
| Anti-pHistone H3 (Ser10) | Abcam | Cat# ab5176, RRID:AB_304763 | |
| Anti-IL-13Rα1 | LSBio | Cat# LS-C117959 | |
|  |  |  | |
| Anti-Elp3 (D5H12) | Cell Signaling | Cat# 5728, RRID:AB_11178379 | |
| Anti-pSTAT6 (Tyr641) | Cell signaling | Cat# 9361, RRID:AB_331595 | |
| Anti-STAT6 | Cell Signaling | Cat# 9362, RRID:AB_2271211 | |
| Anti-pJAK2 (Tyr1007/1008) | Cell signaling | Cat# 3771, RRID:AB_330403 | |
| Anti-JAK1 (6G4) | Cell signaling | Cat# 3344, RRID:AB_2265054 | |
| Anti-Atf4 (D4B8) | Cell signaling | Cat# 11815, RRID:AB_2616025 | |
| Anti-IRE1a (14C10) | Cell signaling | Cat# 3294, RRID:AB_823545 | |
| Anti-BIP (C50B12) | Cell signaling | Cat# 3177, RRID:AB_2119845 | |
| Anti-Atf6 (D4Z8V) | Cell signaling | Cat# 65880, RRID:AB_2799696 | |
| Anti-peIF2α (Ser51) | Cell signaling | Cat# 9721, RRID:AB_330951 | |
| Anti-eIF2α | Cell signaling | Cat# 9722, RRID:AB_2230924 | |
| Anti-CHOP (L63F7) | Cell signaling | Cat# 2895, RRID:AB_2089254 | |
| Anti-XBP1s (D2C1F) | Cell Signaling | Cat# 12782, RRID:AB_2687943 | |
| Anti-pS6 Ribosomal Protein (Ser235/236) (D57.2.2E) | Cell signaling | Cat# 4858, RRID:AB_916156 | |
| Anti-S6 Ribosomal Protein (5G10) | Cell signaling | Cat# 2217, RRID:AB_331355 | |
| Anti-PHGDH | Cell Signaling | Cat# 13428, RRID:AB_2750870 | |
| Anti-GAPDH (D16H11) | Cell Signaling | Cat# 5174, RRID:AB_10622025 | |
| Anti-p4E-BP1 (Thr37 / Thr46) (236B4) | Cell signaling | Cat# 2855, RRID:AB_560835 | |
| Anti-4E-BP1 (53H11) | Cell signaling | Cat# 9644, RRID:AB_2097841 | |
| Anti-Tuberin/TSC2 (D93F12) | Cell Signaling | Cat# 4308, RRID:AB_10547134 | |
| Anti-Hamartin/TSC1 (D43E2) | Cell Signaling | Cat# 6935, RRID:AB_10860420 | |
| Anti-Aldolase A (D73H4) | Cell Signaling | Cat# 8060, RRID:AB_2797635 | |
| Anti-Hexokinase I (C35C4) | Cell Signaling | Cat# 2024, RRID:AB_2116996 | |
| Anti-Pkm2 (D78A4) | Cell Signaling | Cat# 4053, RRID:AB_1904096 | |
| Anti-Enolase 1 | Cell Signaling | Cat# 3810, RRID:AB_2246524 | |
| Anti-Enolase 2 (E2H9X) | Cell Signaling | Cat# 24330, RRID:AB_2868543 | |
| Anti-Pgam1 (D3J9T) | Cell Signaling | Cat# 12098, RRID:AB_2736922 | |
| Anti-Pgk1 | Cell Signaling | Cat# 68540 | |
|  |  |  | |
| Anti-PSAT1 | Proteintech | Cat# 10501-1-AP, RRID:AB_2172597 | |
| Anti-ASNS | Proteintech | Cat# 14681-1-AP, RRID:AB_2060119 | |
|  |  |  | |
| Anti-Mucin2 (H-300) | Santa Cruz Biotechnology | Cat# sc-15334, RRID:AB_2146667 | |
| Anti-JAK2 (C-10) | Santa Cruz Biotechnology | Cat# sc-390539, RRID:AB_2885075 | |
| Tyk2 (C-8) | Santa Cruz Biotechnology | Cat# sc-5271, RRID:AB_628419 | |
| Anti-Chr-A (C-12) | Santa Cruz Biotechnology | Cat# sc-393941, RRID:AB_2801371 | |
| Anti-NPRL2 (F-3) | Santa Cruz Biotechnology | Cat# sc-376986 | |
|  |  |  | |
|  |  |  | |
| Anti-β-Actin-Peroxidase | Sigma-Aldrich | Cat# A3854, RRID:AB_262011 | |
| Anti-Flag | Sigma-Aldrich | Cat# F7425 | |
|  |  |  | |
| Anti-Elp3 | Abclonal Technology | Cat# A0020 | |
| Anti-Dclk1 mouse | Invitrogen | Cat# MA5-26800 | |
| Anti-Ctu2 | Novus | Cat# NBP1-88457 | |
|  |  |  | |
| Sheep anti-Mouse IgG, HRP Conjugated | Cytiva | Cat# NA931, RRID:AB_772210 | |
| Donkey anti-Rabbit IgG, HRP Conjugated | Cytiva | Cat# NA934, RRID:AB_772206 | |
|  |  |  | |
| Anti-Ki67 (B56) | BD Biosciences | Cat# 550609, RRID:AB_393778 | |
| Brilliant Violet 711 Hamster Anti-Mouse KLRG1 Monoclonal Antibody (2F1) | BD Biosciences | Cat# 564014, RRID:AB_2738542 | |
|  |  |  | |
| Goat anti-Mouse IgG (H+L) Antibody, Alexa Fluor 488 Conjugated | Thermo Fisher Scientific | Cat# A-11001, RRID:AB_2534069 | |
| Goat anti-Mouse IgG (H+L) Antibody, Alexa Fluor 568 Conjugated | Thermo Fisher Scientific | Cat# A-11004, RRID:AB_2534072 | |
| Goat anti-Rabbit IgG (H+L) Antibody, Alexa Fluor 488 Conjugated | Thermo Fisher Scientific | Cat# A-11008, RRID:AB_143165 | |
| Goat anti-Rabbit IgG (H+L) Antibody, Alexa Fluor 568 Conjugated | Thermo Fisher Scientific | Cat# A-11036, RRID:AB_10563566 | |
| APC anti-mouse CD45R (B220) Monoclonal Antibody (RA3-6B2) | Thermo Fisher Scientific | Cat# 17-0452-82, RRID:AB_469395 | |
| eFluor-450 CD90.2 (Thy-1.2) Monoclonal Antibody (53-2.1) | Thermo Fisher Scientific | Cat# 48-0902-82, RRID:AB_1272200 | |
| Alexa Fluor 488 anti-mouse IL-13 Monoclonal Antibody (eBio13A) | Thermo Fisher Scientific | Cat# 53-7133-82, RRID:AB_2016708 | |
| Alexa-Fluor 488 anti-mouse GATA3 Monoclonal Antibody (TWAJ) | Thermo Fisher Scientific | Cat# 53-9966-42, RRID:AB_2574493 | |
|  |  |  | |
| Purified anti-mouse CD16/32 Monoclonal Antibody (93) | Biolegend | Cat# 101301, RRID:AB_312800 | |
| APC anti-mouse CD3ε Monoclonal Antibody (145-2C11) | Biolegend | Cat# 100311, RRID:AB_312676 | |
| APC anti-mouse F4/80 Monoclonal Antibody (BM8) | Biolegend | Cat# 123115, RRID:AB_893493 | |
| APC anti-mouse FcεR1 Monoclonal Antibody (MAR1) | Biolegend | Cat# 134316, RRID:AB_10640121 | |
| APC anti-mouse CD11c Monoclonal Antibody (N418) | Biolegend | Cat# 117309, RRID:AB_313778 | |
| APC anti-mouse CD170 (Siglec-F) Monoclonal Antibody (S17007L) | Biolegend | Cat# 155507, RRID:AB_2750236 | |
| APC anti-mouse CD4 Monoclonal Antibody (RM4-5) | Biolegend | Cat# 100515, RRID:AB_312718 | |
| APC anti-mouse CD8a Monoclonal Antibody (53-6.7) | Biolegend | Cat# 100711, RRID:AB_312750 | |
| APC anti-mouse CD5 Monoclonal Antibody (53-7.3) | Biolegend | Cat# 100625, RRID:AB_2563928 | |
| APC anti-mouse CD49b Antibody (HMα2) | Biolegend | Cat# 103515, RRID:AB_2566100 | |
| APC anti-mouse Ly-6G/Ly-6C (Gr-1) Monoclonal Antibody (RB6-8C5) | Biolegend | Cat# 108412, RRID:AB_313377 | |
| PE-Cyanine 7 rat anti-mouse CD45 Monoclonal Antibody (30-F11) | Biolegend | Cat# 103114, RRID:AB_312979 | |
| FITC anti-mouse Ly6A/E (Sca1) Monoclonal Antibody (W18174A) | Biolegend | Cat# 160907, RRID:AB_2910335 | |
|  |  |  | |
| **Chemicals, Peptides, and Recombinant Proteins** |  |  | |
|  |  |  | |
| PBS | Lonza | Cat# 17-516F | |
| DPBS | Lonza | Cat# 17-512F | |
| HBSS with Ca2+/Mg2+ | Gibco | Cat# 14025-050 | |
| HBSS without Ca2+/Mg2+ | Gibco | Cat# 14175-095 | |
| EDTA | Millipore | Cat# 324503 | |
| HEPES | CarlRoth | Cat# 9105.4 | |
| Recombinant Mouse IL-13 (carrier-free) (for mouse treatment) | Biolegend | Cat# 575908 | |
| IL-13 Monoclonal Antibody (eBio1316H) | Invitrogen | Cat# 16-7135-85, RRID:AB_763563 | |
| Isrib | Sigma-Aldrich | Cat# SML0843 | |
| Dibenzazepine YO-01027 | MedChemExpress | Cat# 50-149-3990 | |
| Rapamycin | LC Laboratories | Cat# R-5000 | |
| DMSO | Sigma-Aldrich | Cat# D2438 | |
| PEG-400 | Sigma-Aldrich | Cat# 8.07485 | |
| Tween-80 | Sigma-Aldrich | Cat# P4780 | |
| (Hydroxypropyl)methyl cellulose | Sigma-Aldrich | Cat# H7509 | |
| Collagenase D | Roche | Cat# 11088858001 | |
| DNaseI | Roche | Cat# 10104159001 | |
| Dispase II | Sigma-Aldrich | Cat# D4693 | |
| Fetal Bovine Serum (FBS) | Gibco | Cat# 10270106 | |
| Liberase TM | Roche | Cat# 5401119001 | |
| Phorbol 12-myristate 13-acetate | Tocris | Cat# 1201 | |
| Ionomycin | Tocris | Cat# 1704 | |
| Monensin | Biolegend | Cat# 420701 | |
| Brefeldin A | Biolegend | Cat# 420601 | |
| β-mercaptoethanol | Thermo Scientific | Cat# 21985023 | |
| DL-Dithiothreitol DTT | Sigma-Aldrich | Cat# D9779 | |
| Ultrapure BSA | Invitrogen | Cat# AM2616 | |
| Adenosine 5′-triphosphate disodium salt hydrate (ATP) | Sigma-Aldrich | Cat# A7699 | |
| Formaldehyde | Sigma-Aldrich | Cat# F8775 | |
| Sucrose | Sigma-Aldrich | Cat# 84097 | |
| Neg-50™ Frozen Section Medium | Epredia | Cat# 6502 | |
| Antigen Unmasking Solution, Citrate-Based | Vector Laboratories | Cat# H-3300-250 | |
| Triton X-100 | Acros Organics | Cat# 215680025 | |
| Tween-20 | Fisher Bioreagents | Cat# BP337-500 | |
| BSA | Cell Signaling | Cat# 9998 | |
| DAPI | Sigma-Aldrich | Cat# D9542 | |
| ProLong™ Gold Antifade Mountant | Invitrogen | Cat# P36930 | |
| Alcian Blue | Sigma-Aldrich | Cat# 1016470500 | |
| Eosin Y | VWR | Cat# 341972Q | |
| Pierce BCA protein assay | Thermo Scientific | Cat# 23227 | |
| Amersham Protran 0.45 NC nitrocellulose Western blotting membranes | Cytiva | Cat# 10600002 | |
| Pierce™ ECL Western Blotting Substrate | Thermo Scientific | Cat# 32106 | |
| SuperSignal™ West Femto Maximum Sensitivity Substrate | Thermo Scientific | Cat# 34096 | |
| TriPure™ Isolation Reagent | Roche | Cat# 11667165001 | |
| TB Green® Premix Ex Taq™ (Tli RNase H Plus), ROX Plus | Takara Bio | Cat# RR42LR | |
| Matrigel® Growth Factor Reduced (GFR) Basement Membrane Matrix, LDEV-free | Corning | Cat# 354230 | |
| IntestiCult™ Organoid Growth Media | Stemcell | Cat# 06005 | |
| Penicillin/Streptomycin | Westburg | Cat# CA PS-B | |
| ACCUMAX™ Cell detachment solution | Stemcell | Cat# 07921 | |
| Transdux | Systembio | Cat# LV850A-1 | |
| CHIR99021 | Sigma-Aldrich | Cat# SML1046 | |
| Recombinant Mouse IL-13 Protein (organoid treatment) | R&D Systems | Cat# 413-ML | |
| Thapsigargin | Sigma-Aldrich | Cat# SML1845 | |
| Puromycin | InvivoGen | Cat# ant-pr-1 | |
|  |  |  | |
|  |  |  | |
| **Critical Commercial Assays** |  |  | |
|  |  |  | |
| ATP CellTiter-Glo® Luminescent Cell Viability Assay | Promega | Cat# G7572 | |
| Red Blood Cell Lysis Solution (10×) | Miltenyi Biotec | Cat# 130-094-183 | |
| E.Z.N.A.® Total RNA Kit I | Omega Bio-Tek | Cat# R6834-01 | |
| Revert Aid H Minus First Strand cDNA Synthesis kit  RediPlate 96 Ribogreen RNA Kit | Thermo Scientific  Invitrogen | Cat# K1632  Cat# R32700 | |
|  |  |  | |
| **Experimental models: Organisms/strains** |  |  | |
|  |  |  | |
| Mouse: *Elp3-floxed* |  |  | |
| Mouse: *Villin-Cre* | Jackson Laboratory | Cat# 004586 | |
| Mouse: *Rosa26-ATF4loxtg* | Jackson Laboratory | Cat# 029394 | |
| *N. brasiliensis* | from Dr. Benjamin Dewals | N/A | |
|  |  |  | |
| **Recombinant DNAs and ShRNAs** |  |  | |
|  |  |  | |
| pLV[Exp]-Puro-CMV>mCherry | This study | VectorBuilder | |
| pLV[Exp]-Puro-CMV>mAtf4[NM_009716.3]*/mCherry | This study | VectorBuilder | |
| pLV[Exp]-mCherry:T2A:Puro-EF1A>mAsns[NM_001410606.1] | This study | VectorBuilder | |
| shAtf4 #1 - TRCN0000301645 | This study | Sigma-Aldrich | |
| shAtf4 #2 - TRCN0000301646 | This study | Sigma-Aldrich | |
| pLV[Exp]-Hygro-EF1A>FLAG/mNprl2[NM_018879.2] | This study | VectorBuilder | |
| pLV[Exp]-Hygro-EF1A>FLAG/mNprl2[NM_018879.2] Mutant | This study | VectorBuilder | |
| shNprl2 #1 - TRCN0000042420 | This study | Sigma-Aldrich | |
| shNprl2 #2 - TRCN0000042422 | This study | Sigma-Aldrich | |
| mouse shElp3 #1 - TRCN0000039310 | This study | Sigma-Aldrich | |
| mouse shElp3 #2 - TRCN0000039312 | This study | Sigma-Aldrich | |
| human shElp3 - TRCN0000235508 | This study | Sigma-Aldrich | |
| human shCtu2 - TRCN0000165286 | This study | Sigma-Aldrich | |

| **Oligonucleotides** | **Forward** | **Reverse** |
| --- | --- | --- |
|  |  |  |
| β-Actin | GACGGCCAGGTCATCACTAT | ATGCCACAGGATTCCATACC |
| β2m | CACCCCACTGAGACTGATACA | TGATGCTTGATCACATGTCTCG |
| Gapdh | TTTGCCGTGAGTGGAGTCATA | TGCAGTGGCAAAGTGGAGAT |
| 36b4 | ATGGGTACAAGCGCCTCCTG | GCCTTGACCTTTTCAGTAAG |
| Dclk1 | CAAGCCAGCCATGTCGTTC | TTCCTTTGAAGTAGCGGTCAC |
| Tas1R3 | CTTCCTCATGCCACAGGTCAG | TGTGCGGAAGAAGGATGGAA |
| Tas2R105 | CTGGGCTGGGAGTTTTAGGG | CCTGGAAGTTGCTAAGCCGA |
| Tas2R117 | TGGAAACCGACATGTGGACAA | GCCTTAACACCTGCCTGTGA |
| Tas2R136 | TGGAGGAACCAATCCACCTG | AACCATTGCCAACACAAAGGC |
| Tas2R143 | TCCCAGTTAGTTCCCAGGCT | AAGTTCCCGGTGGCTGAAAT |
| SucnR1 | CCGACAGCAGAATGGCACAG | TCCCAAGCAGTCCAAAAATGAA |
| Ffar3 | TTTCTGAGCGTGGCCTATCC | AACCACACTACAGTGAGCCG |
| Trpm5 | AAGCATCACGAGCAACAGCC | TTGCATGGTGGCTTTCCAGT |
| IL-25 | ACAGGGACTTGAATCGGGTC | TGGTAAAGTGGGACGGAGTTG |
| Retnlβ | AAGCCTACACTGTGTTTCCTTTT | GCTTCCTTGATCCTTTGATCCAC |
| IL-4Rα | ACTGGATCTGGGAGCATCAA | CTATCCAGGAACCACTCACACG |
| IL-13Rα1 | GAAGGTGATCCTGAGTCCGC | AGTGTGTGTCAGGGCTTGTA |
| Asns | GACTCTAAGGTGGGAAGCGG | CAGGCACTCTGAGCACTAGC |
| Atf5 | AGATGAGGTCCTCCACCTTCG | AATGGAGGCTGCACCAACAA |
| Eif4ebp1 | GGAGAGCTGCACAGCATTCAGG | GGAGGTATGTGCTGGTGTTCAC |
| Phgdh | CCTCCTTTGGTGTTCAGCAGCT | CGCACACCTTTCTTGCACTGAG |
| Psat1 | CATTGGCAACGCCAAAGGAGAC | GTGACAGCGTTATACAGAGAGGC |
| Psph | CCACATCTGACTCCTGGCATAAG | AGCTTTGCAGCAACGTGCTCCA |
| Nprl2 | TGACCATCCTGCTGGAAGAGCT | CTGCTCGATCACCTTCAAGTGG |
| Sesn2 | CGGCACTCAGAGAAGGTTCA | ACGGGGTAGTCAGGTCATGT |
| Chop | GGAGAGAGTGTTCCAGAAGGAAG | CGTCTCCAAGGTGAAAGGCA |
| Gadd34 | AGAGGCGGCTCAGATTGTTC | CGAAGTGTACCTTCCGAGCTT |
| Stc2 | GCTGTGGTGTGTTTGAGTGTT | ATGAATGACTTTCCCTGGGCAT |
| Defa5 | AGGCTGATCCTATCCACAAAACAG | TGAAGAGCAGACCCTTCTTGGC |
| ChgA | ATCCTCTCTATCCTGCGACAC | GGGCTCTGGTTCTCAAACACT |
| Lgr5 | CCTGGGAAAGCATACCCGTT | GGTTGACTCACAGGACCGTT |
| Ascl2 | AAGCACACCTTGACTGGTACG | AAGTGGACGTTTGCACCTTCA |
| Atoh1 | AGCTTCCTCTGGGGGTTACT | TTCTGTGCCATCATCGCTGT |
| Sis | ACCCTCCTTTTACTCCCGGT | TGCACAGCATCCATGCAAAG |
| Mex3a | AGAGCCTCACGCAACAAGTCTG | CTGGATGCGTTTGATGGTCGCT |
| Colca2 | ACCTCAGGGGCAAACCTGTC | TGGAAGACGTTGGTTCAGGCT |
| Igfbp7 | TTCAGCGGACAGAACTCTTGCC | GTTGGATGCGTGGCACTCATAC |
| Tead2 | AAGGTCTGCTCCTTTGGCAAGC | CTGACGGAGCTTGTGCAGGAAA |
| Polr3g | CCTGATGTTGTGTTGAAGCCTCC | CACTGTTTCCCTCAACTCCTGC |
| Ldhb | CCTCAGATCGTCAAGTACAGCC | ATCCGCTTCCAATCACACGGTG |
| Hpgds | GGAGCAATGTCAAGCTGATGCAG | TCAGAAGGCGAGGTGCTTGATG |
| Ptgs1 | GAATGCCACCTTCATCCGAGAAG | GCTCACATTGGAGAAGGACTCC |
| Ptgs2 | GCGACATACTCAAGCAGGAGCA | AGTGGTAACCGCTCAGGTGTTG |
| Elp3 | GGGAGGAAGTGGATTCTCTG | ACGTCTTTCCCCTGCTCAT |
