# Supplementary figures and images for: Loss of Elp3 blocks intestinal tuft cell differentiation via an mTORC1-Atf4 axis (part 1 of 2)
Source: EMBO J. 2024 Jul 31;43(18):6. doi: 10.1038/s44318-024-00184-4 (PMC11405396; doi:10.1038/s44318-024-00184-4)

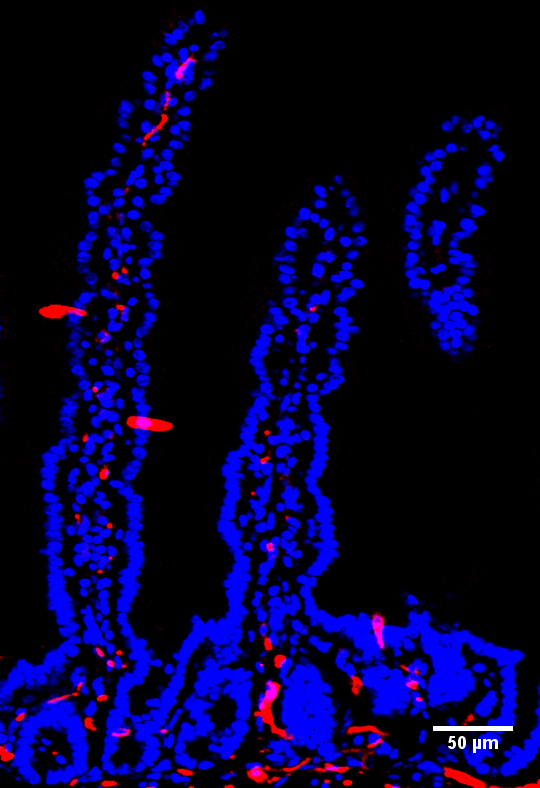

Supplement: Supplementary file 4 — Source data Fig. 1 [file 44318_2024_184_MOESM4_ESM.zip › Figure 1/1A/IF Dclk1 KO NI.tif]

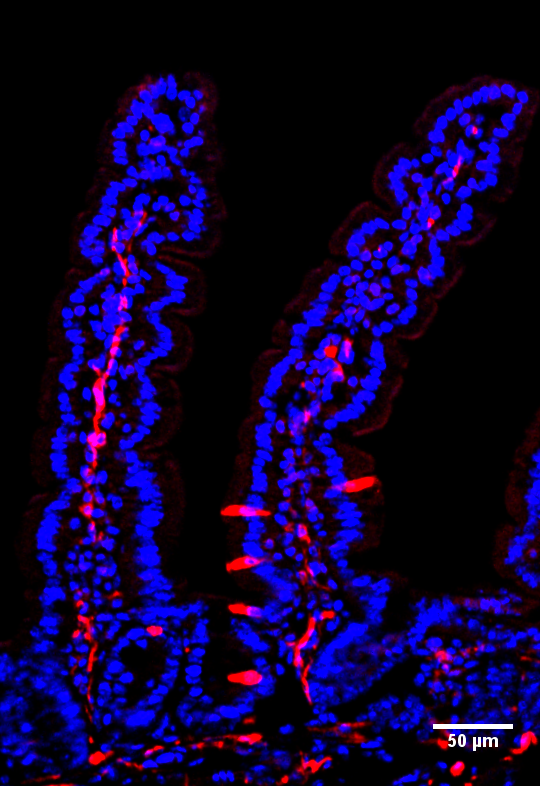

Supplement: Supplementary file 4 — Source data Fig. 1 [file 44318_2024_184_MOESM4_ESM.zip › Figure 1/1A/IF Dclk1 WT NI.tif]

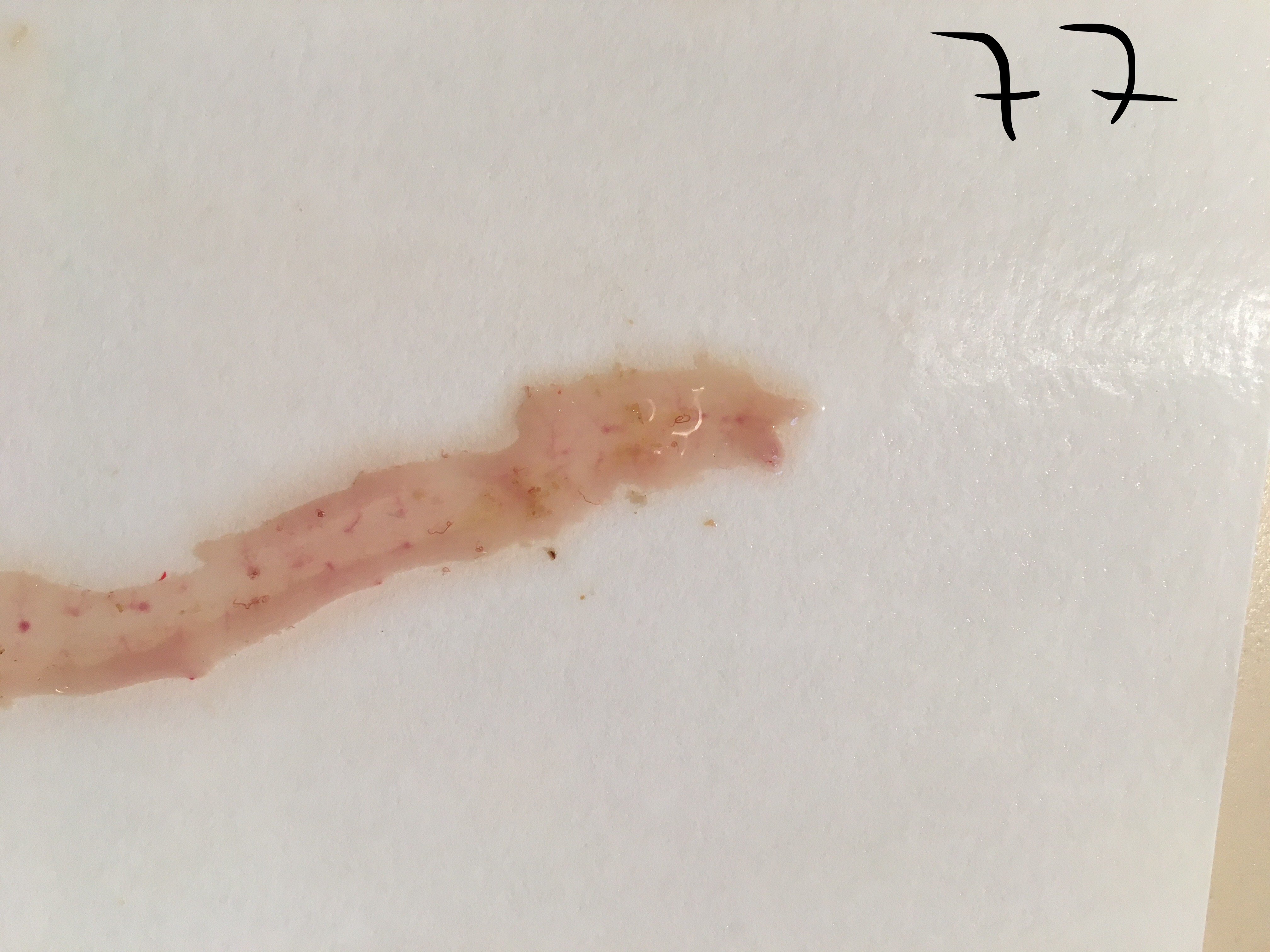

Supplement: Supplementary file 4 — Source data Fig. 1 [file 44318_2024_184_MOESM4_ESM.zip › Figure 1/1D/Elp3KO mouse + Nb D7.jpg]

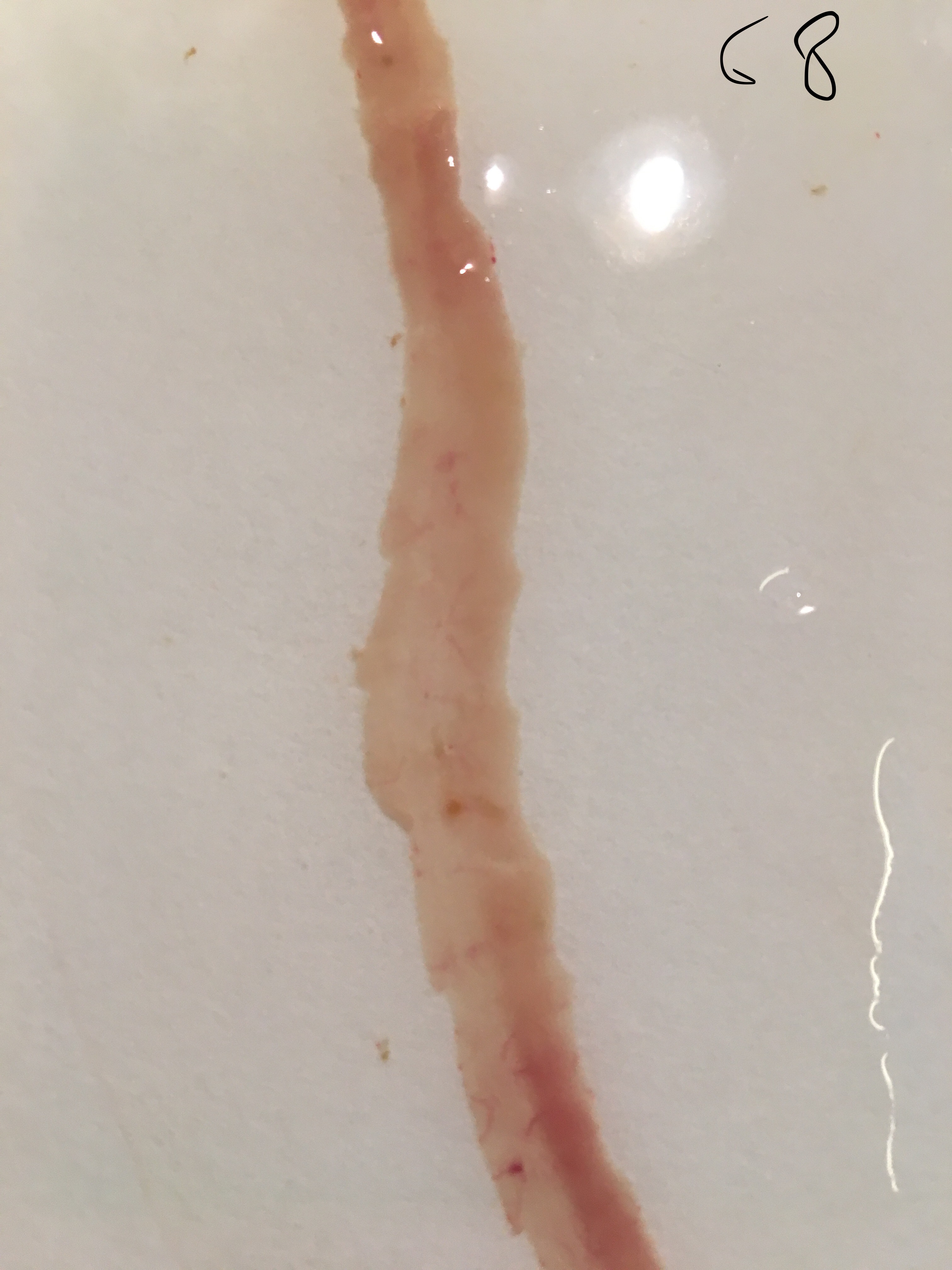

Supplement: Supplementary file 4 — Source data Fig. 1 [file 44318_2024_184_MOESM4_ESM.zip › Figure 1/1D/Elp3WT mouse + Nb D7.jpg]

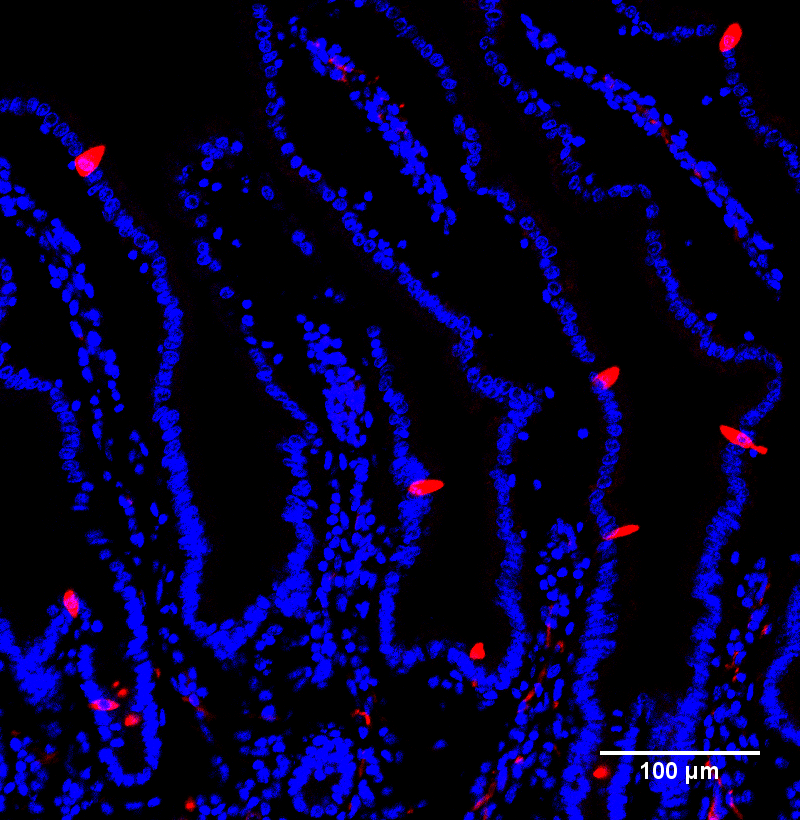

Supplement: Supplementary file 4 — Source data Fig. 1 [file 44318_2024_184_MOESM4_ESM.zip › Figure 1/1F/IF Dclk1 KO Nb D7.tif]

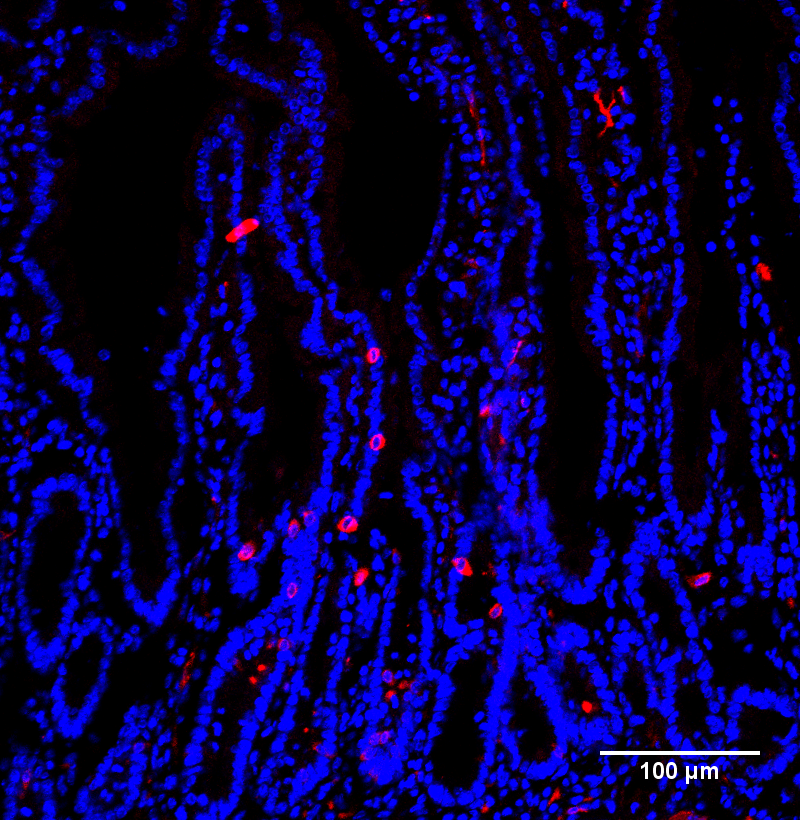

Supplement: Supplementary file 4 — Source data Fig. 1 [file 44318_2024_184_MOESM4_ESM.zip › Figure 1/1F/IF Dclk1 KO Nb D9.tif]

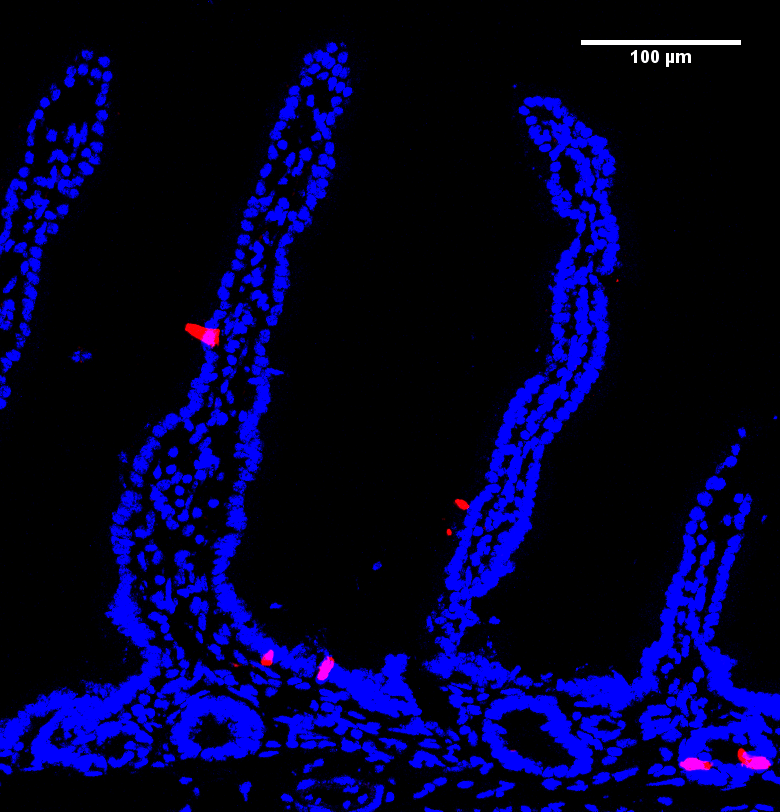

Supplement: Supplementary file 4 — Source data Fig. 1 [file 44318_2024_184_MOESM4_ESM.zip › Figure 1/1F/IF Dclk1 KO NI.tif]

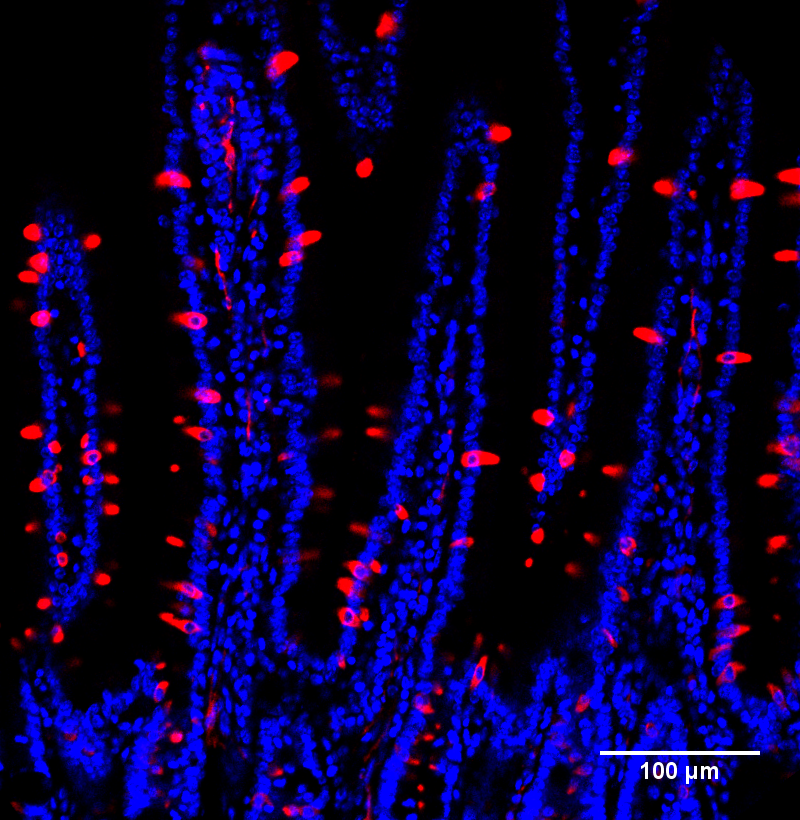

Supplement: Supplementary file 4 — Source data Fig. 1 [file 44318_2024_184_MOESM4_ESM.zip › Figure 1/1F/IF Dclk1 WT Nb D7.tif]

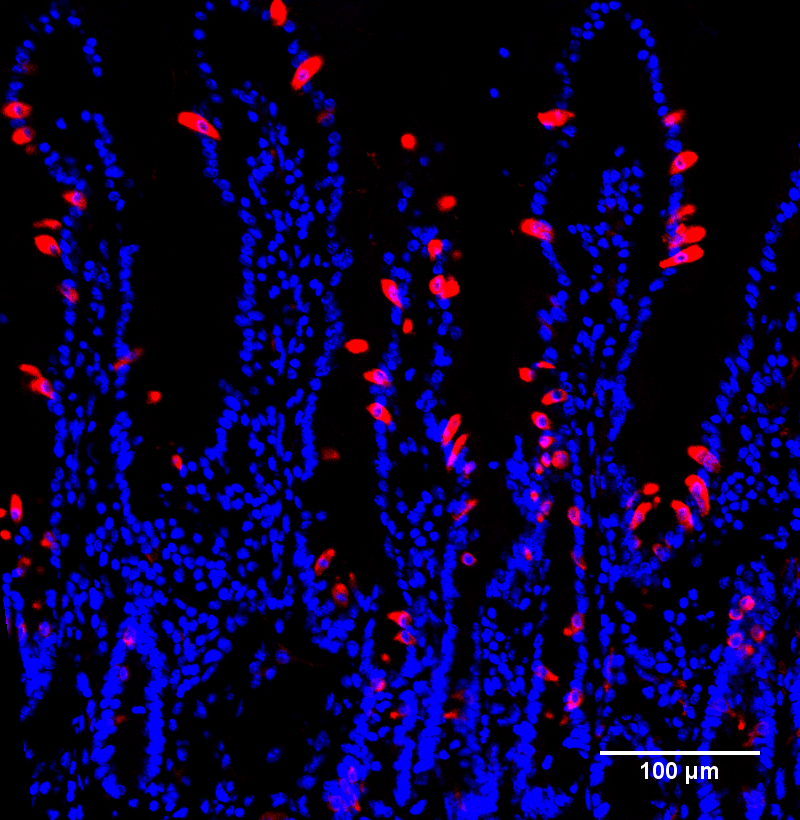

Supplement: Supplementary file 4 — Source data Fig. 1 [file 44318_2024_184_MOESM4_ESM.zip › Figure 1/1F/IF Dclk1 WT Nb D9.tif]

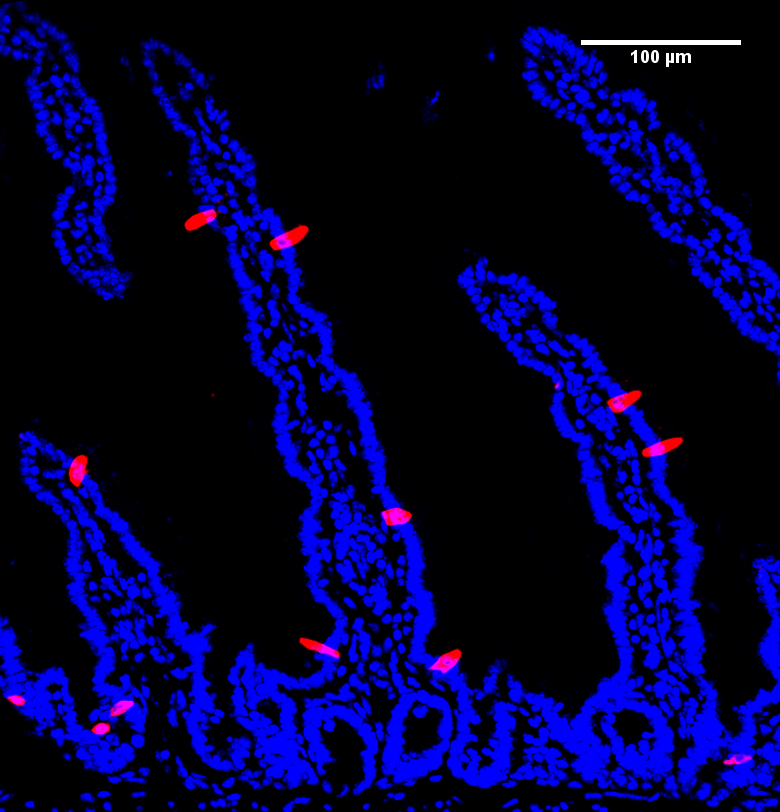

Supplement: Supplementary file 4 — Source data Fig. 1 [file 44318_2024_184_MOESM4_ESM.zip › Figure 1/1F/IF Dclk1 WT NI.tif]

## Slide 1
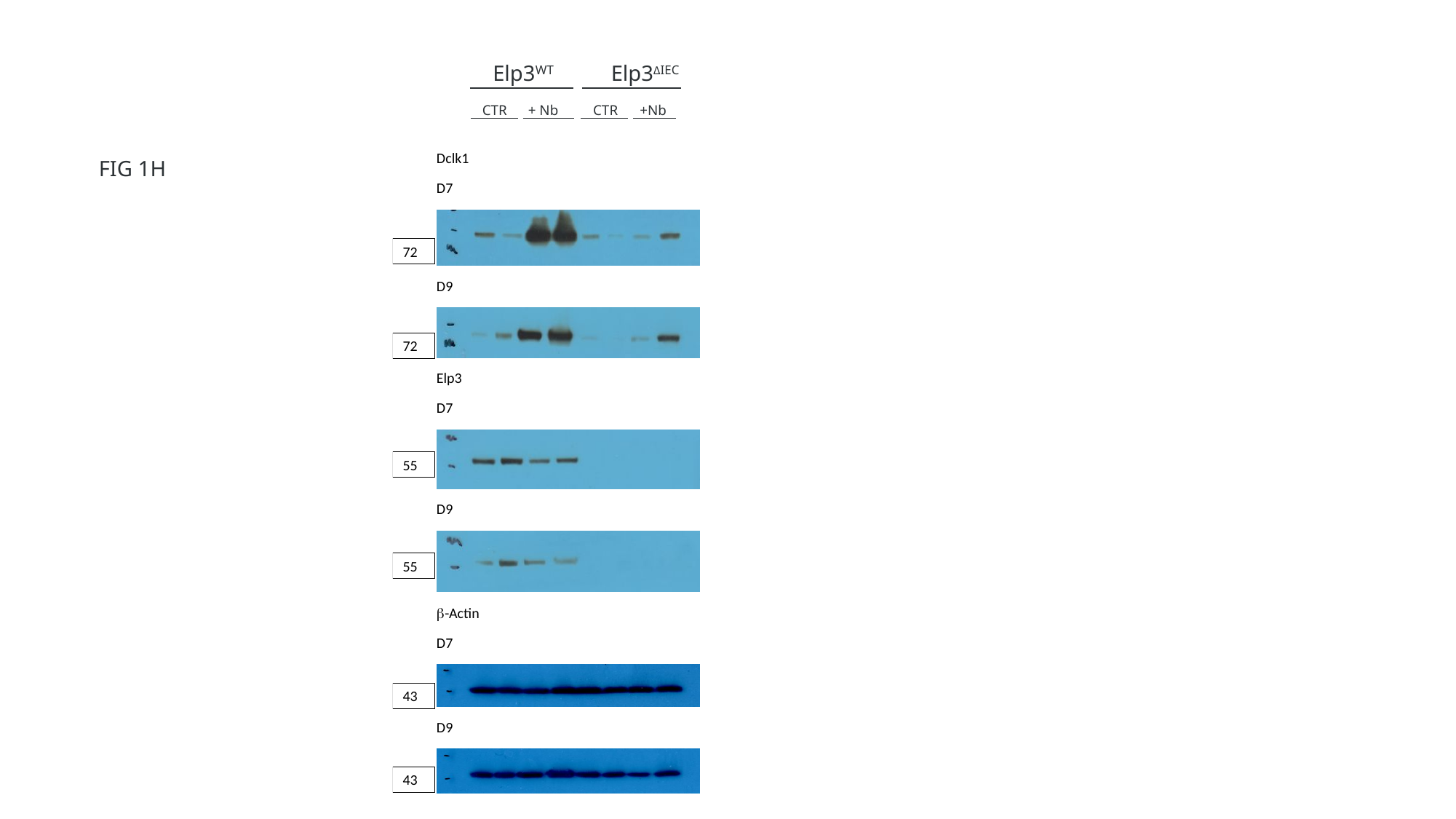

Elp3WT
Elp3ΔIEC
CTR
+ Nb
CTR
+Nb
FIG 1H

Supplement: Supplementary file 4 — Source data Fig. 1 [file 44318_2024_184_MOESM4_ESM.zip › Figure 1/1H/WB uncropped gels.pptx]

## Slide 1
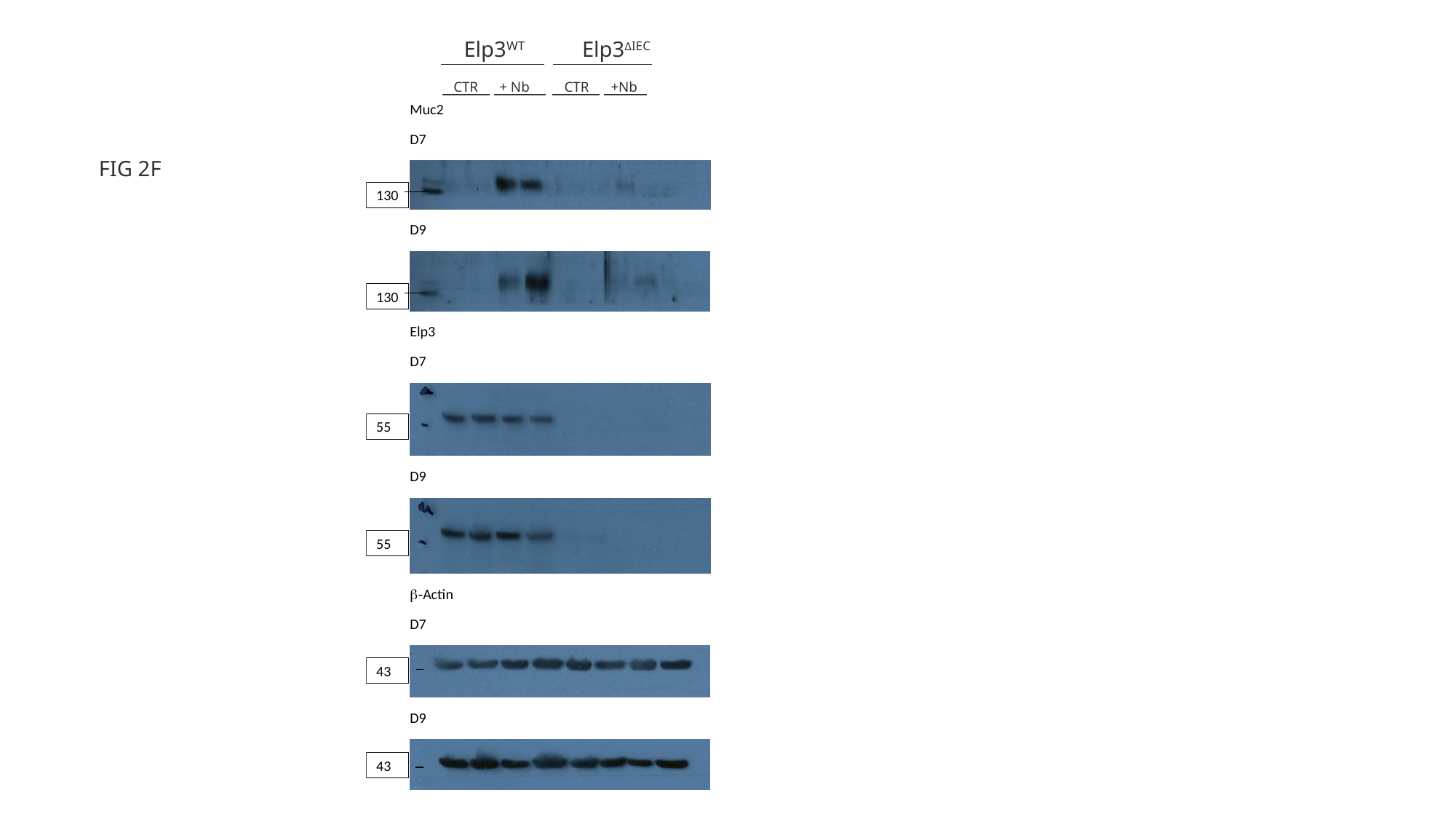

Elp3WT
Elp3ΔIEC
CTR
+ Nb
CTR
+Nb
FIG 2F

Supplement: Supplementary file 5 — Source data Fig. 2 [file 44318_2024_184_MOESM5_ESM.zip › Figure 2/2F/WB uncropped gels.pptx]

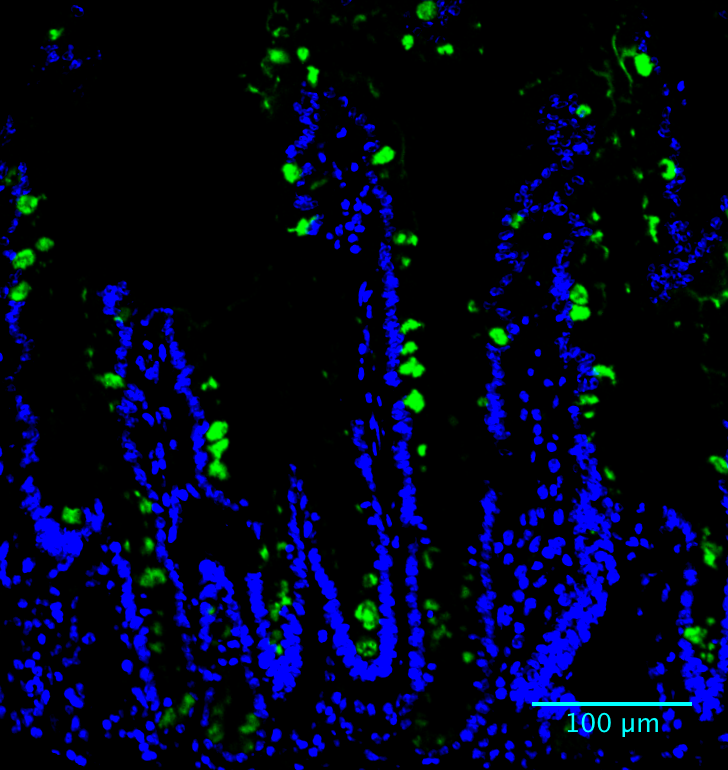

Supplement: Supplementary file 5 — Source data Fig. 2 [file 44318_2024_184_MOESM5_ESM.zip › Figure 2/2G/IF Muc2 KO D7 (RGB).tif]

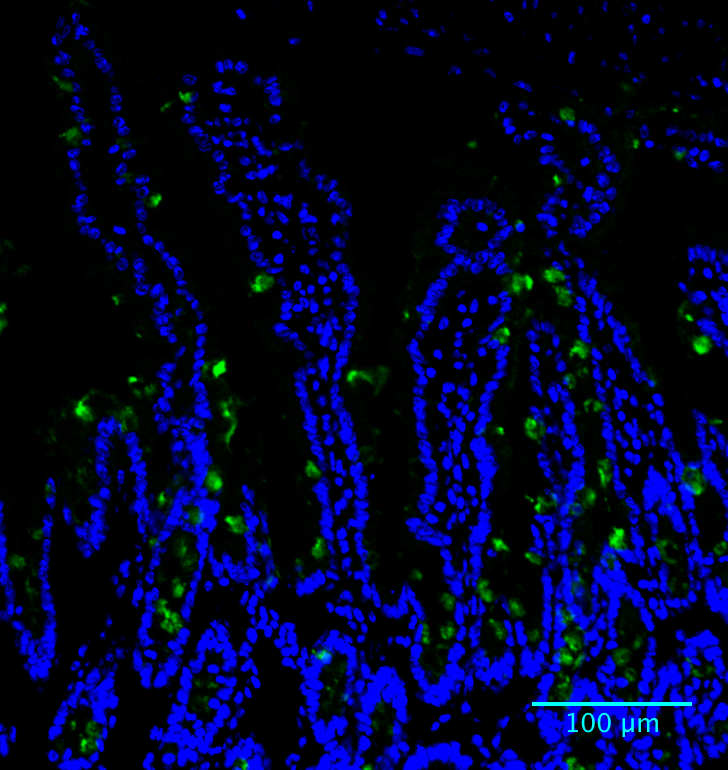

Supplement: Supplementary file 5 — Source data Fig. 2 [file 44318_2024_184_MOESM5_ESM.zip › Figure 2/2G/IF Muc2 KO D9 (RGB).tif]

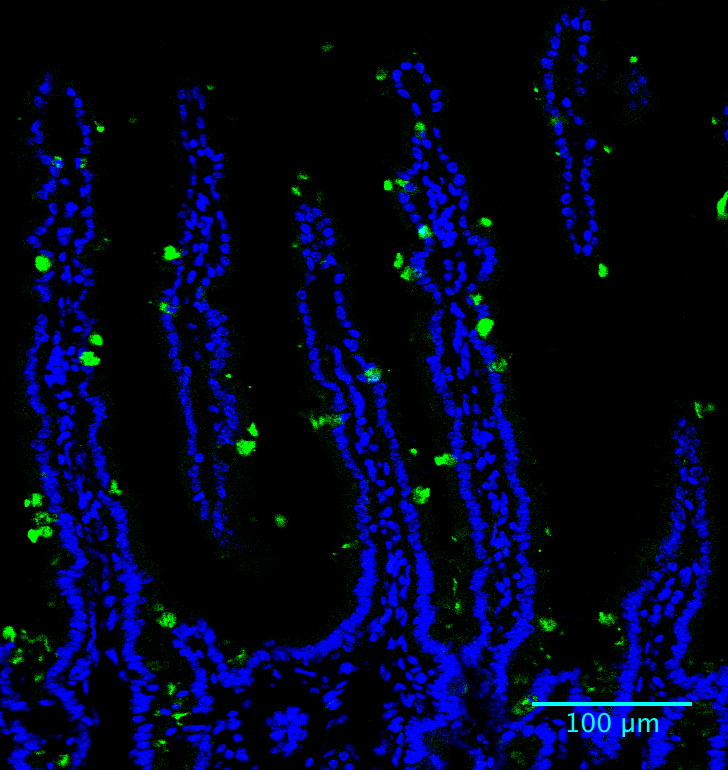

Supplement: Supplementary file 5 — Source data Fig. 2 [file 44318_2024_184_MOESM5_ESM.zip › Figure 2/2G/IF Muc2 KO NI (RGB).tif]

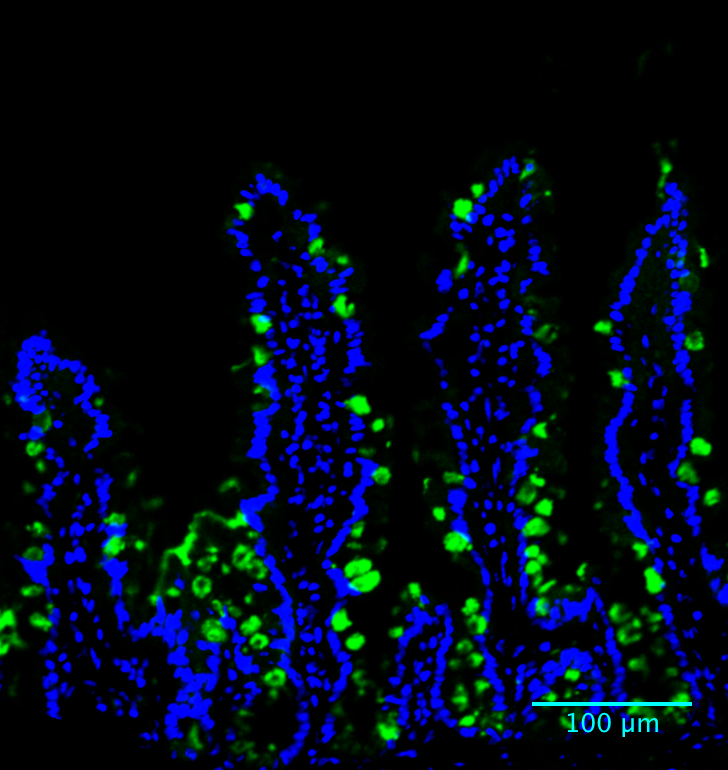

Supplement: Supplementary file 5 — Source data Fig. 2 [file 44318_2024_184_MOESM5_ESM.zip › Figure 2/2G/IF Muc2 WT D7 (RGB).tif]

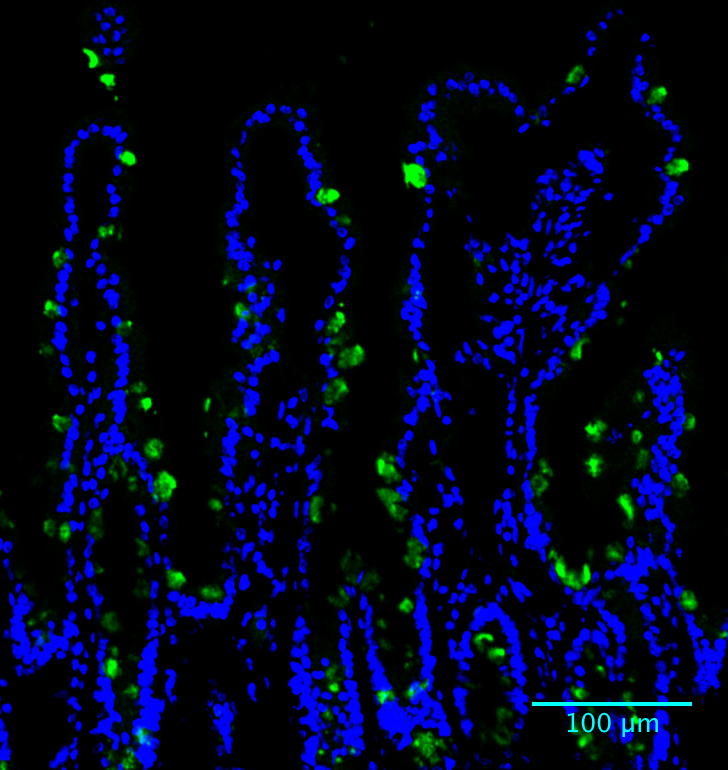

Supplement: Supplementary file 5 — Source data Fig. 2 [file 44318_2024_184_MOESM5_ESM.zip › Figure 2/2G/IF Muc2 WT D9 (RGB).tif]

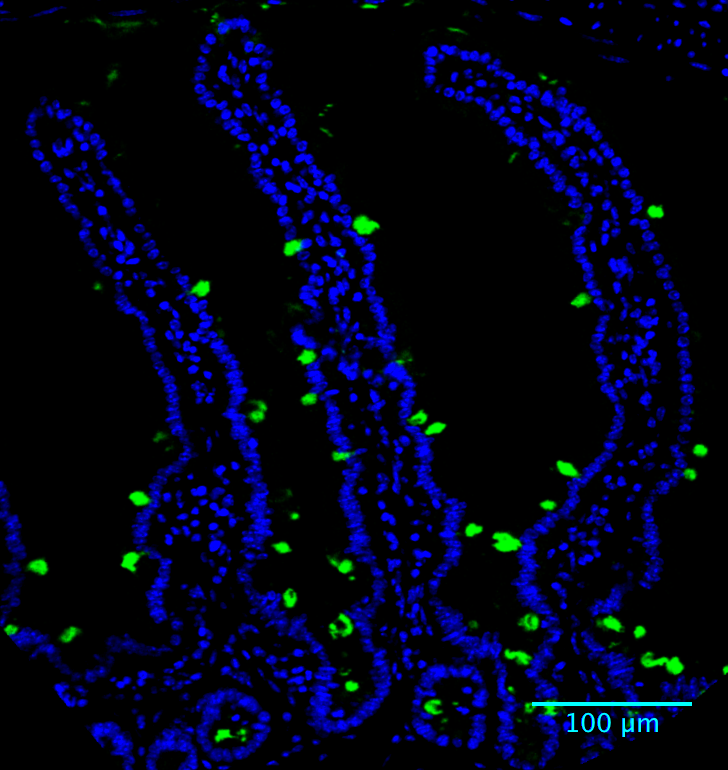

Supplement: Supplementary file 5 — Source data Fig. 2 [file 44318_2024_184_MOESM5_ESM.zip › Figure 2/2G/IF Muc2 WT NI (RGB).tif]

## Slide 1
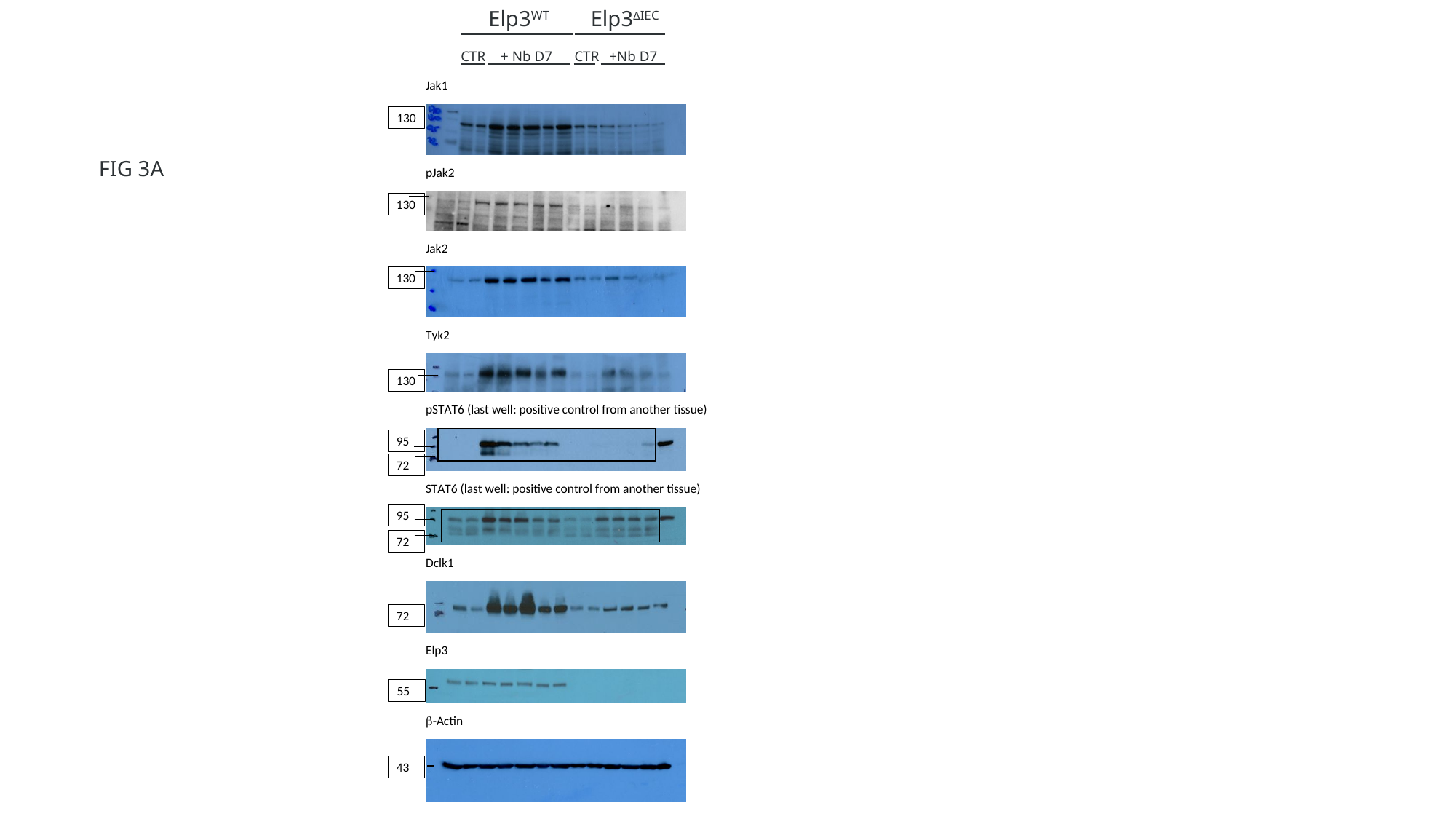

Elp3WT
Elp3ΔIEC
CTR
+ Nb D7
CTR
+Nb D7
FIG 3A

Supplement: Supplementary file 6 — Source data Fig. 3 [file 44318_2024_184_MOESM6_ESM.zip › Figure 3/3A/WB uncropped gels.pptx]

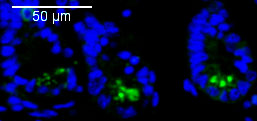

Supplement: Supplementary file 6 — Source data Fig. 3 [file 44318_2024_184_MOESM6_ESM.zip › Figure 3/3C/IF IL13Ra1 KO J7.tif]

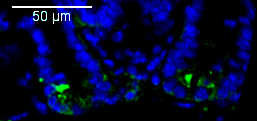

Supplement: Supplementary file 6 — Source data Fig. 3 [file 44318_2024_184_MOESM6_ESM.zip › Figure 3/3C/IF IL13Ra1 KO NI.tif]

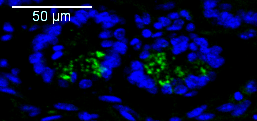

Supplement: Supplementary file 6 — Source data Fig. 3 [file 44318_2024_184_MOESM6_ESM.zip › Figure 3/3C/IF IL13Ra1 WT J7.tif]

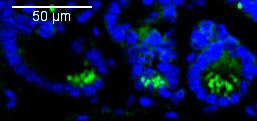

Supplement: Supplementary file 6 — Source data Fig. 3 [file 44318_2024_184_MOESM6_ESM.zip › Figure 3/3C/IF IL13Ra1 WT NI.tif]

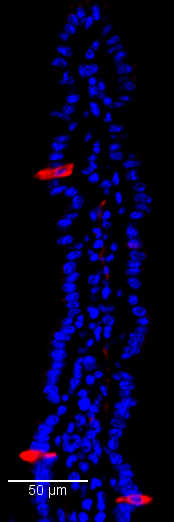

Supplement: Supplementary file 6 — Source data Fig. 3 [file 44318_2024_184_MOESM6_ESM.zip › Figure 3/3D/IF Dclk1 IL13Ra1 KO J7 crop-2.tif]

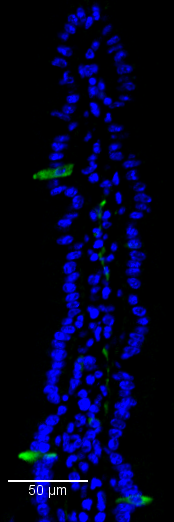

Supplement: Supplementary file 6 — Source data Fig. 3 [file 44318_2024_184_MOESM6_ESM.zip › Figure 3/3D/IF Dclk1 IL13Ra1 KO J7 crop-3.tif]

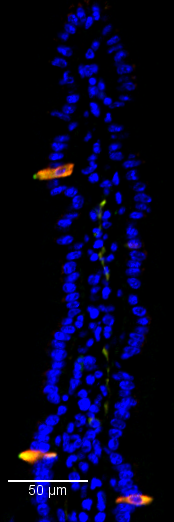

Supplement: Supplementary file 6 — Source data Fig. 3 [file 44318_2024_184_MOESM6_ESM.zip › Figure 3/3D/IF Dclk1 IL13Ra1 KO J7 crop-4.tif]

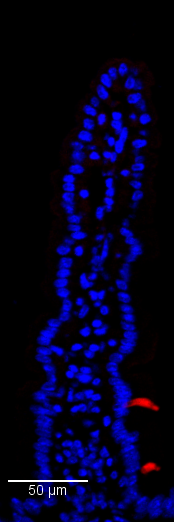

Supplement: Supplementary file 6 — Source data Fig. 3 [file 44318_2024_184_MOESM6_ESM.zip › Figure 3/3D/IF Dclk1 IL13Ra1 KO NI crop-2.tif]

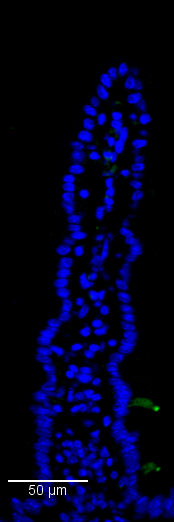

Supplement: Supplementary file 6 — Source data Fig. 3 [file 44318_2024_184_MOESM6_ESM.zip › Figure 3/3D/IF Dclk1 IL13Ra1 KO NI crop-3.tif]

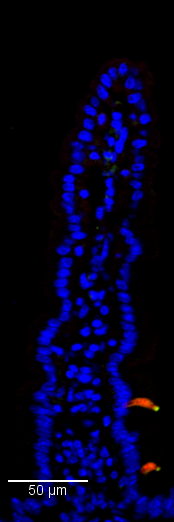

Supplement: Supplementary file 6 — Source data Fig. 3 [file 44318_2024_184_MOESM6_ESM.zip › Figure 3/3D/IF Dclk1 IL13Ra1 KO NI crop-4.tif]

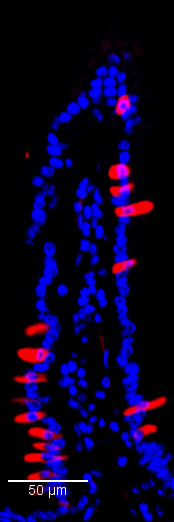

Supplement: Supplementary file 6 — Source data Fig. 3 [file 44318_2024_184_MOESM6_ESM.zip › Figure 3/3D/IF Dclk1 IL13Ra1 WT J7 crop-2.tif]

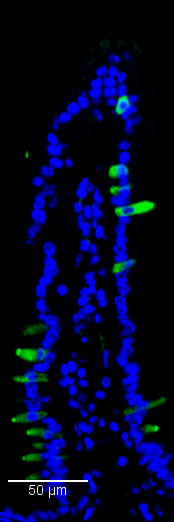

Supplement: Supplementary file 6 — Source data Fig. 3 [file 44318_2024_184_MOESM6_ESM.zip › Figure 3/3D/IF Dclk1 IL13Ra1 WT J7 crop-3.tif]

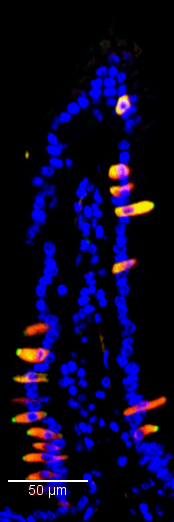

Supplement: Supplementary file 6 — Source data Fig. 3 [file 44318_2024_184_MOESM6_ESM.zip › Figure 3/3D/IF Dclk1 IL13Ra1 WT J7 crop-4.tif]

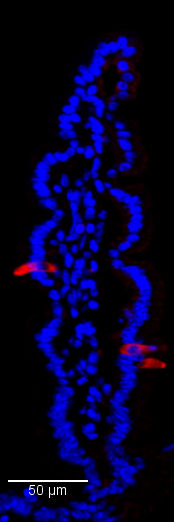

Supplement: Supplementary file 6 — Source data Fig. 3 [file 44318_2024_184_MOESM6_ESM.zip › Figure 3/3D/IF Dclk1 IL13Ra1 WT NI crop-2.tif]

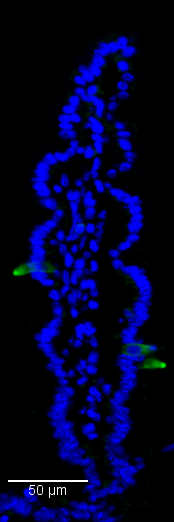

Supplement: Supplementary file 6 — Source data Fig. 3 [file 44318_2024_184_MOESM6_ESM.zip › Figure 3/3D/IF Dclk1 IL13Ra1 WT NI crop-3.tif]

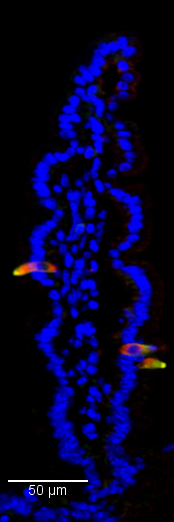

Supplement: Supplementary file 6 — Source data Fig. 3 [file 44318_2024_184_MOESM6_ESM.zip › Figure 3/3D/IF Dclk1 IL13Ra1 WT NI crop-4.tif]

## Slide 1
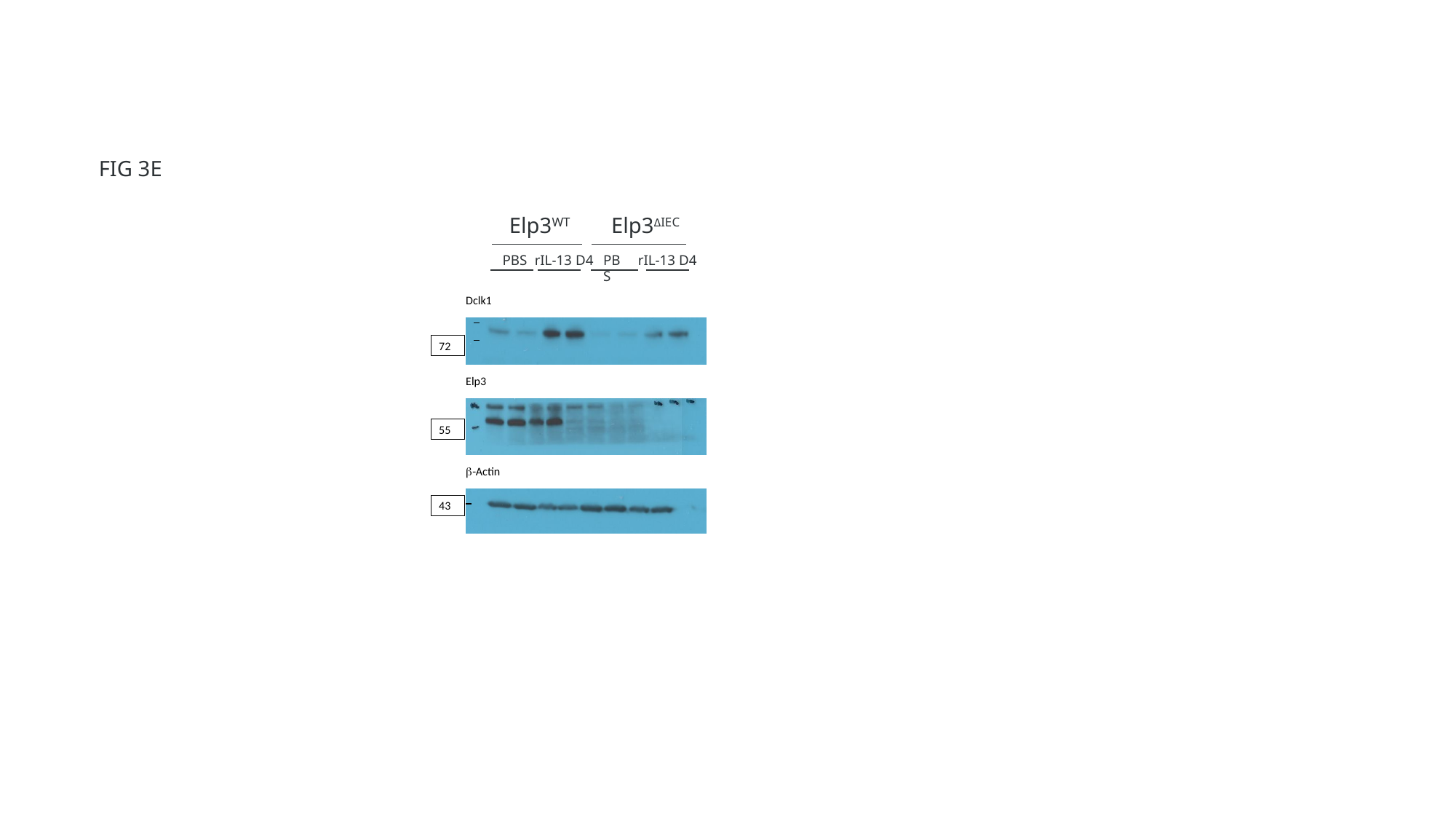

FIG 3E
Elp3WT
Elp3ΔIEC
PBS
rIL-13 D4
PBS
rIL-13 D4

Supplement: Supplementary file 6 — Source data Fig. 3 [file 44318_2024_184_MOESM6_ESM.zip › Figure 3/3E/WB uncropped gels.pptx]

## Slide 1
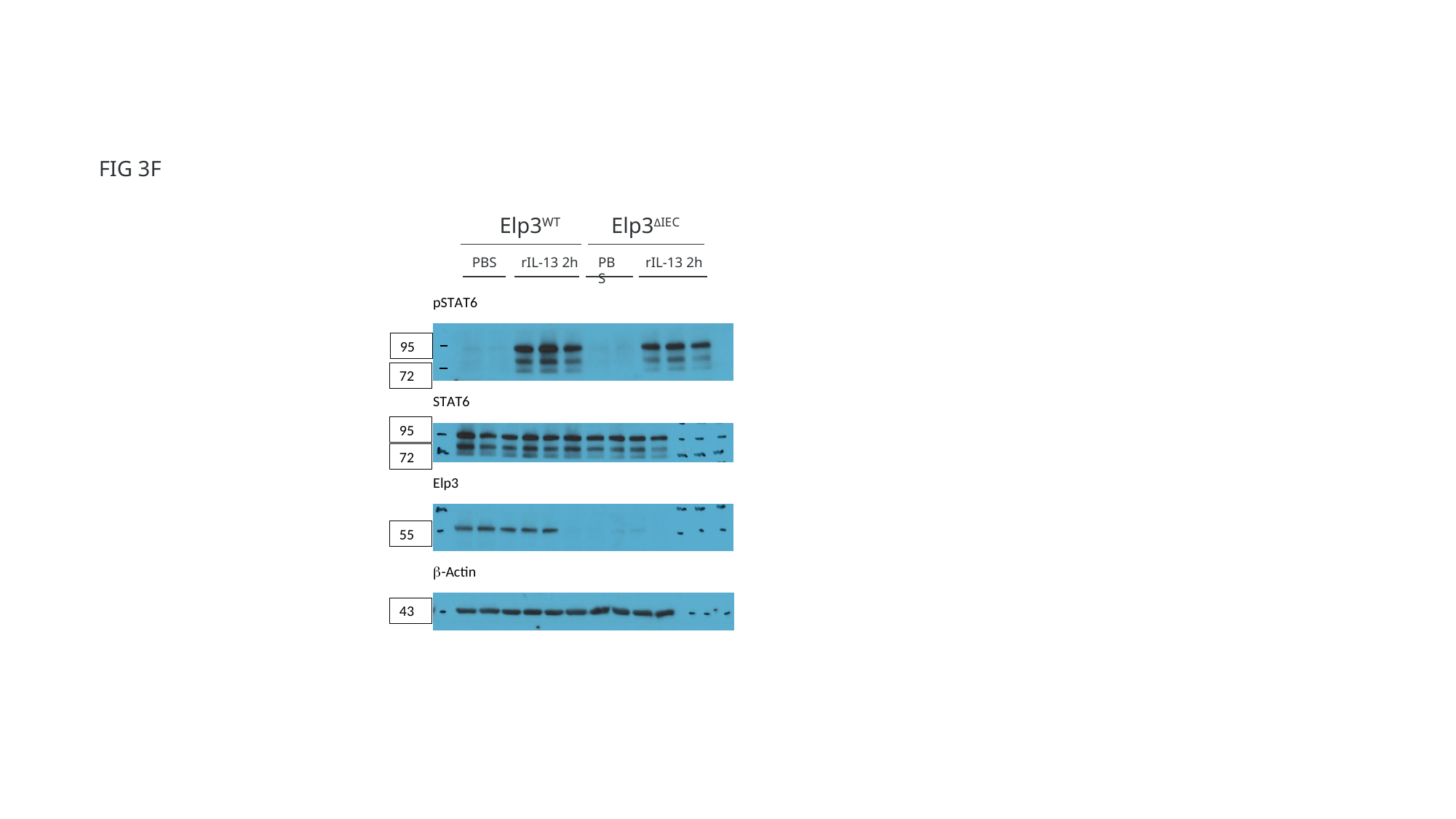

FIG 3F
Elp3WT
Elp3ΔIEC
PBS
rIL-13 2h
PBS
rIL-13 2h

Supplement: Supplementary file 6 — Source data Fig. 3 [file 44318_2024_184_MOESM6_ESM.zip › Figure 3/3F/WB uncropped gels.pptx]

## Slide 1
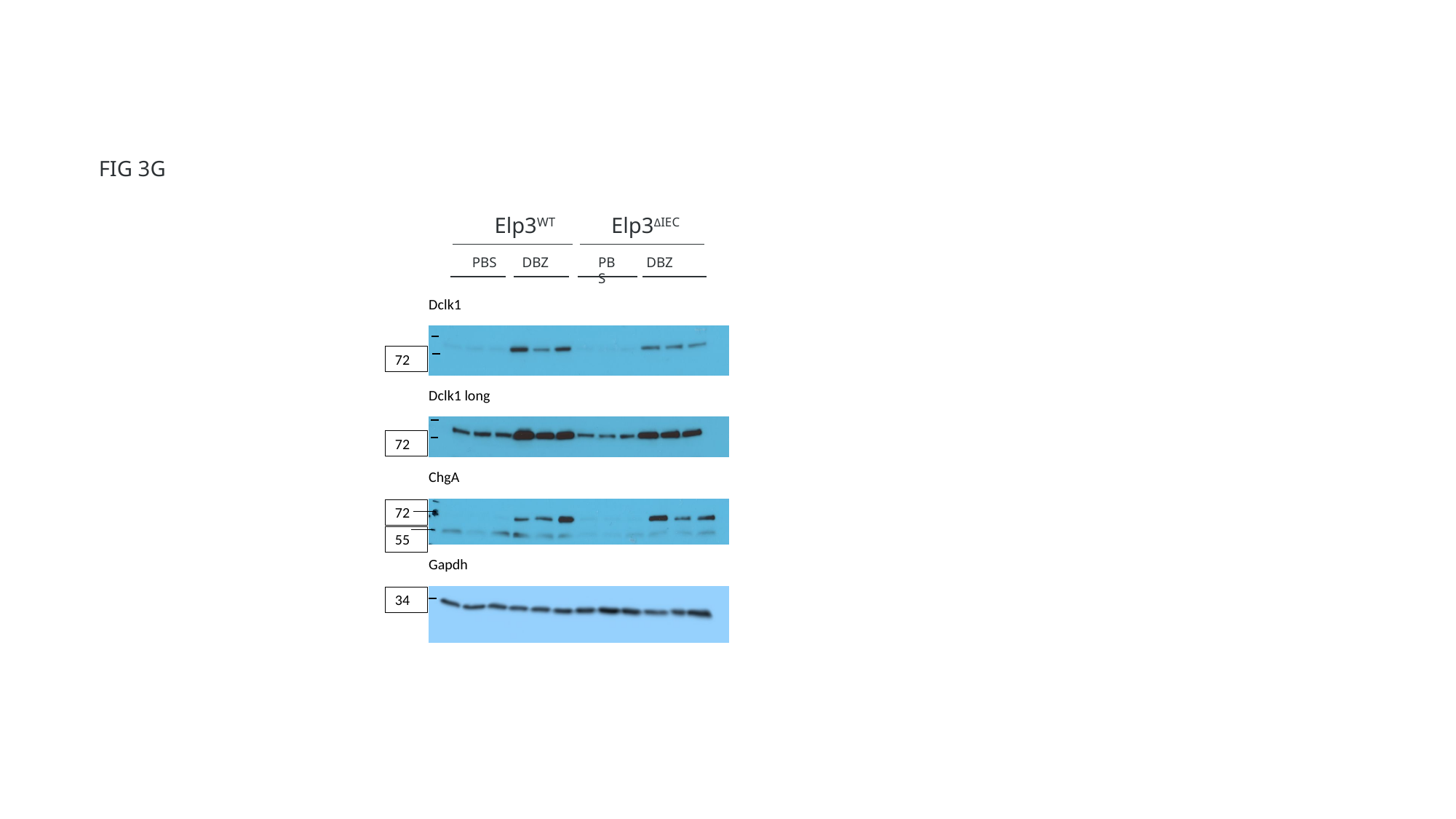

FIG 3G
Elp3WT
Elp3ΔIEC
PBS
DBZ
PBS
DBZ

Supplement: Supplementary file 6 — Source data Fig. 3 [file 44318_2024_184_MOESM6_ESM.zip › Figure 3/3G/WB uncropped gels.pptx]

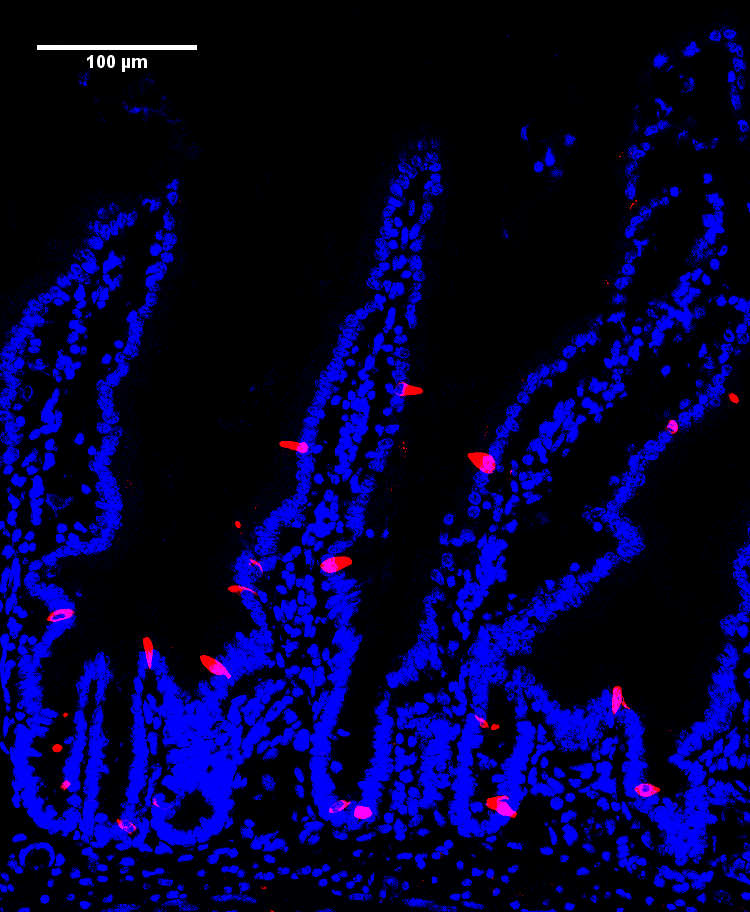

Supplement: Supplementary file 6 — Source data Fig. 3 [file 44318_2024_184_MOESM6_ESM.zip › Figure 3/3H/IF Dclk1 KO DBZ (RGB).tif]

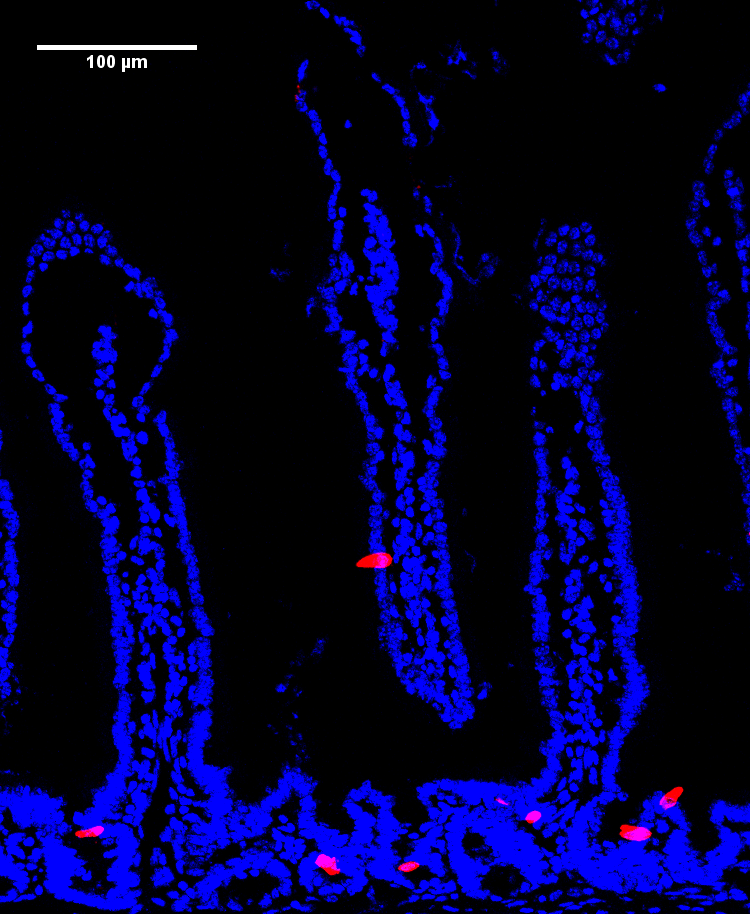

Supplement: Supplementary file 6 — Source data Fig. 3 [file 44318_2024_184_MOESM6_ESM.zip › Figure 3/3H/IF Dclk1 KO VEH (RGB).tif]

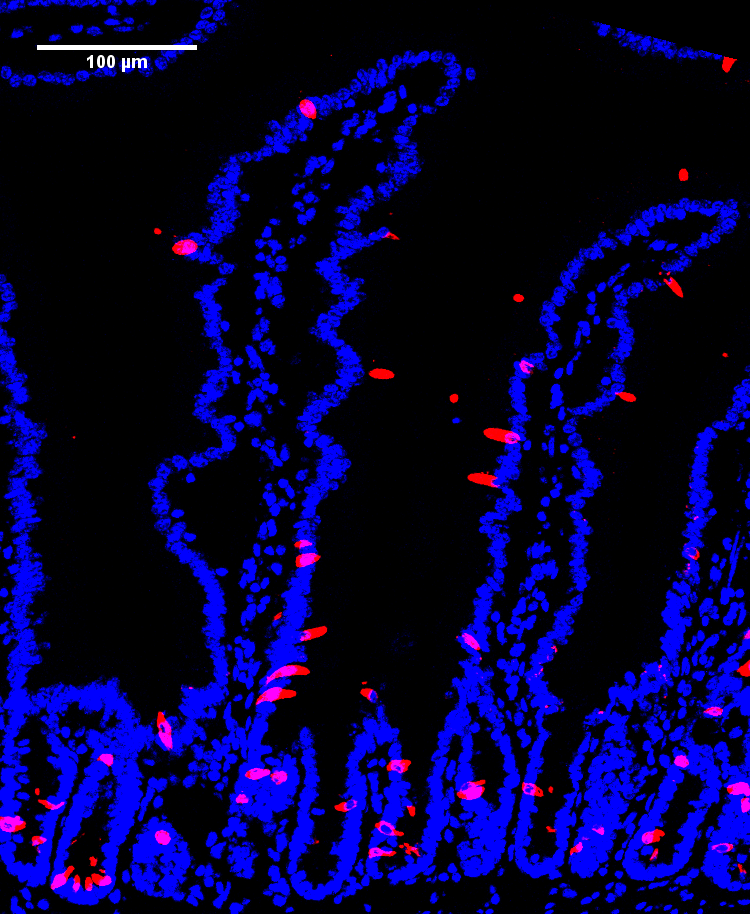

Supplement: Supplementary file 6 — Source data Fig. 3 [file 44318_2024_184_MOESM6_ESM.zip › Figure 3/3H/IF Dclk1 WT DBZ (RGB).tif]

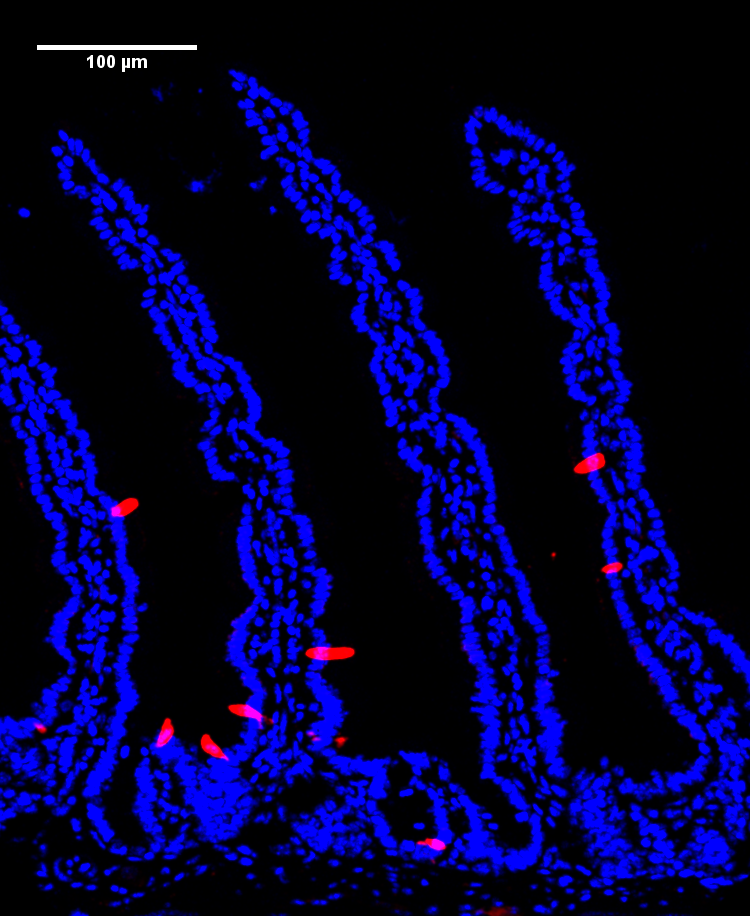

Supplement: Supplementary file 6 — Source data Fig. 3 [file 44318_2024_184_MOESM6_ESM.zip › Figure 3/3H/IF Dclk1 WT VEH (RGB).tif]

## Slide 1
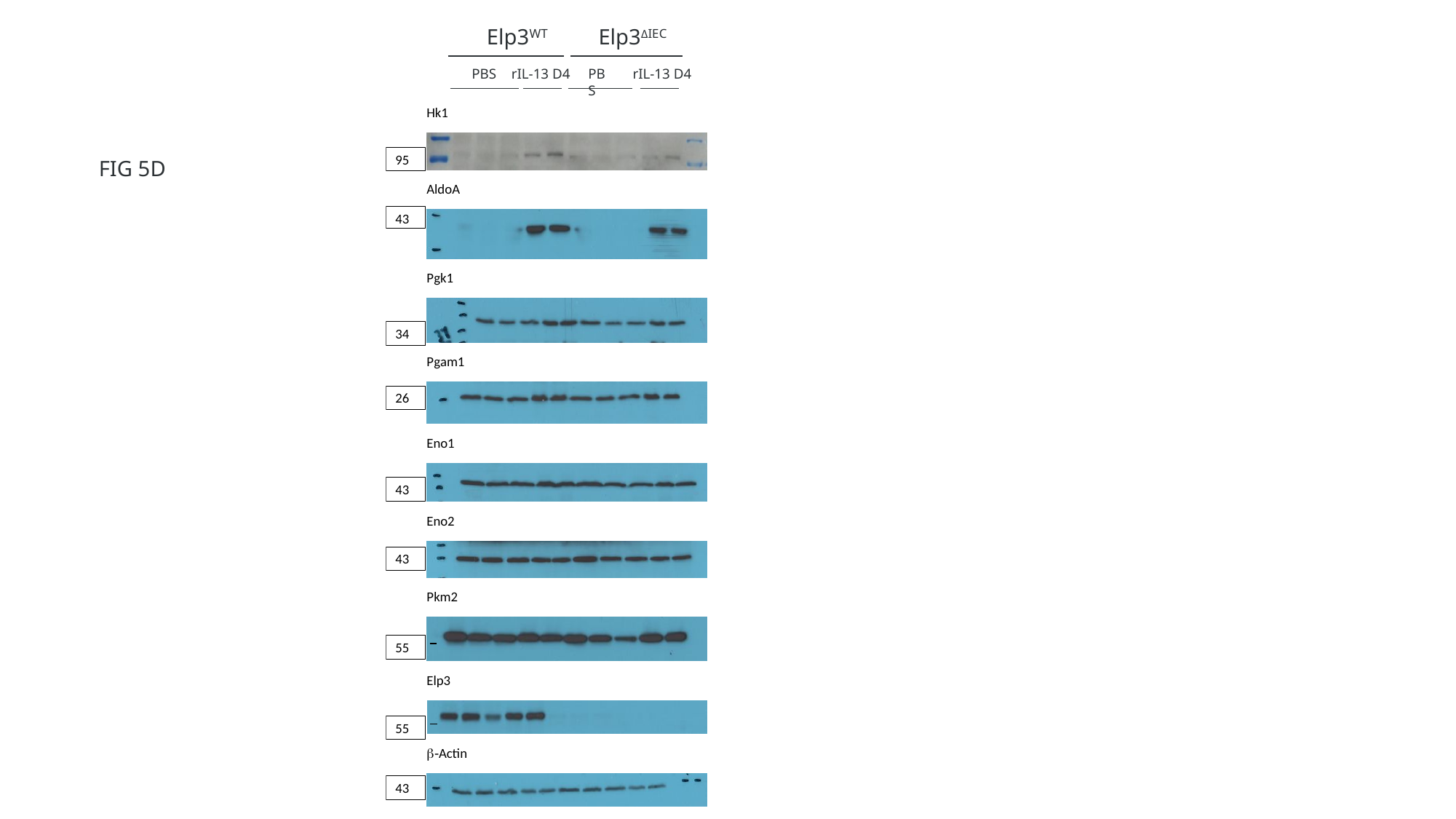

Elp3WT
Elp3ΔIEC
PBS
rIL-13 D4
PBS
rIL-13 D4
FIG 5D

Supplement: Supplementary file 8 — Source data Fig. 5 [file 44318_2024_184_MOESM8_ESM.zip › Figure 5/5D/WB uncropped gels.pptx]

## Slide 1
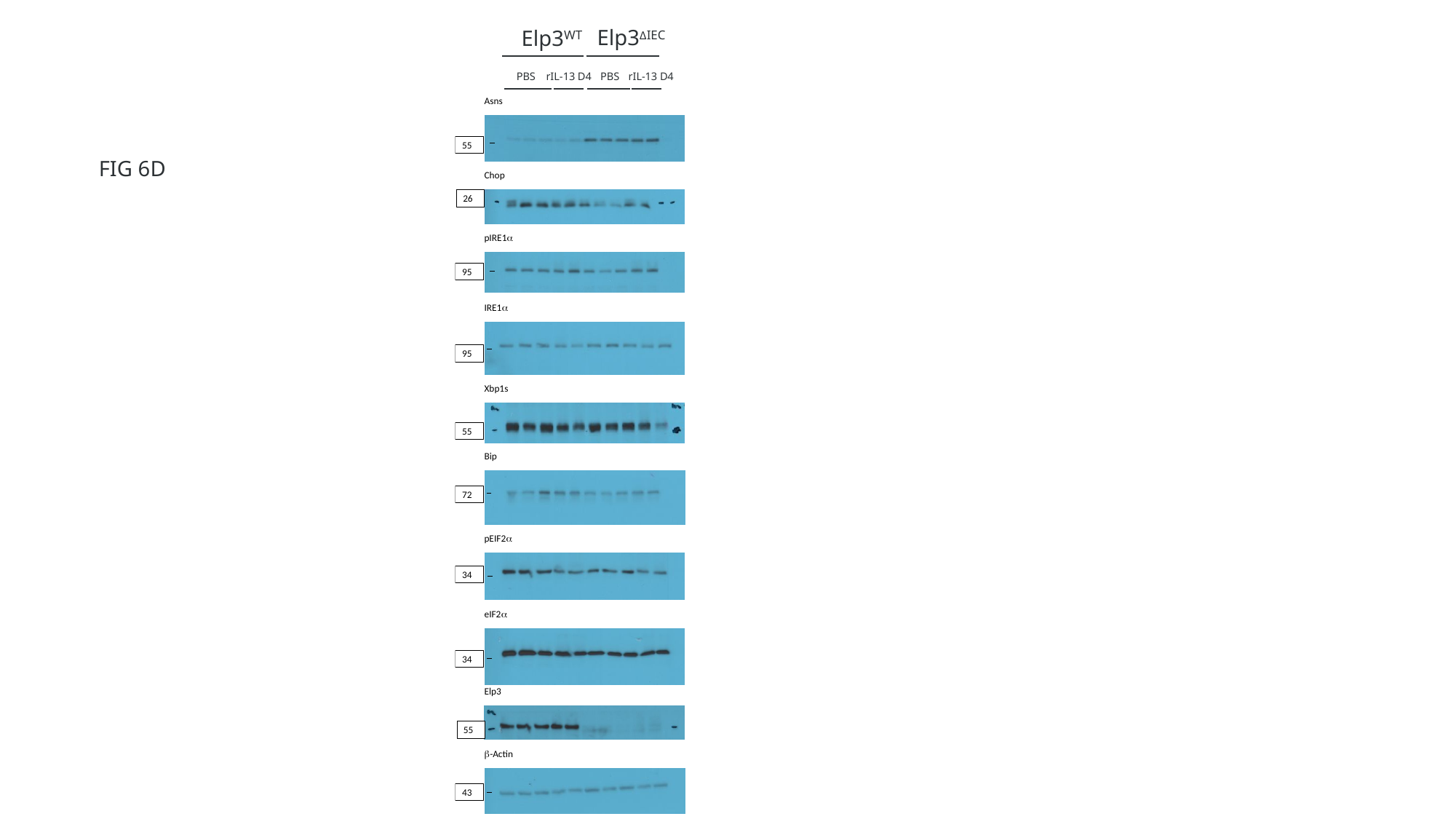

Elp3ΔIEC
Elp3WT
PBS
rIL-13 D4
PBS
rIL-13 D4
FIG 6D

Supplement: Supplementary file 9 — Source data Fig. 6 [file 44318_2024_184_MOESM9_ESM.zip › Figure 6/6D/WB uncropped gels.pptx]

## Slide 1
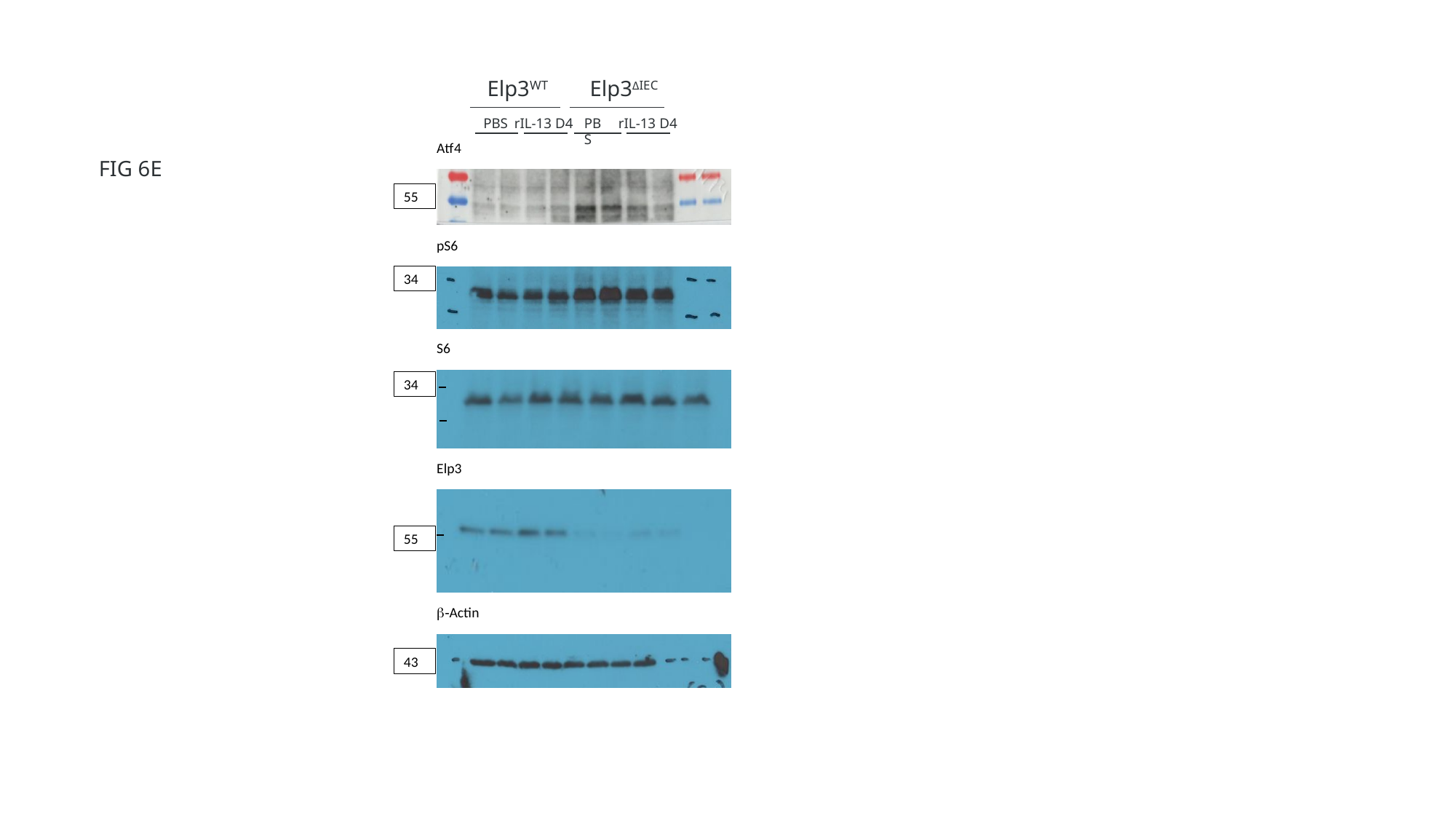

Elp3WT
Elp3ΔIEC
PBS
rIL-13 D4
PBS
rIL-13 D4
FIG 6E

Supplement: Supplementary file 9 — Source data Fig. 6 [file 44318_2024_184_MOESM9_ESM.zip › Figure 6/6E/WB uncropped gels.pptx]

## Slide 1
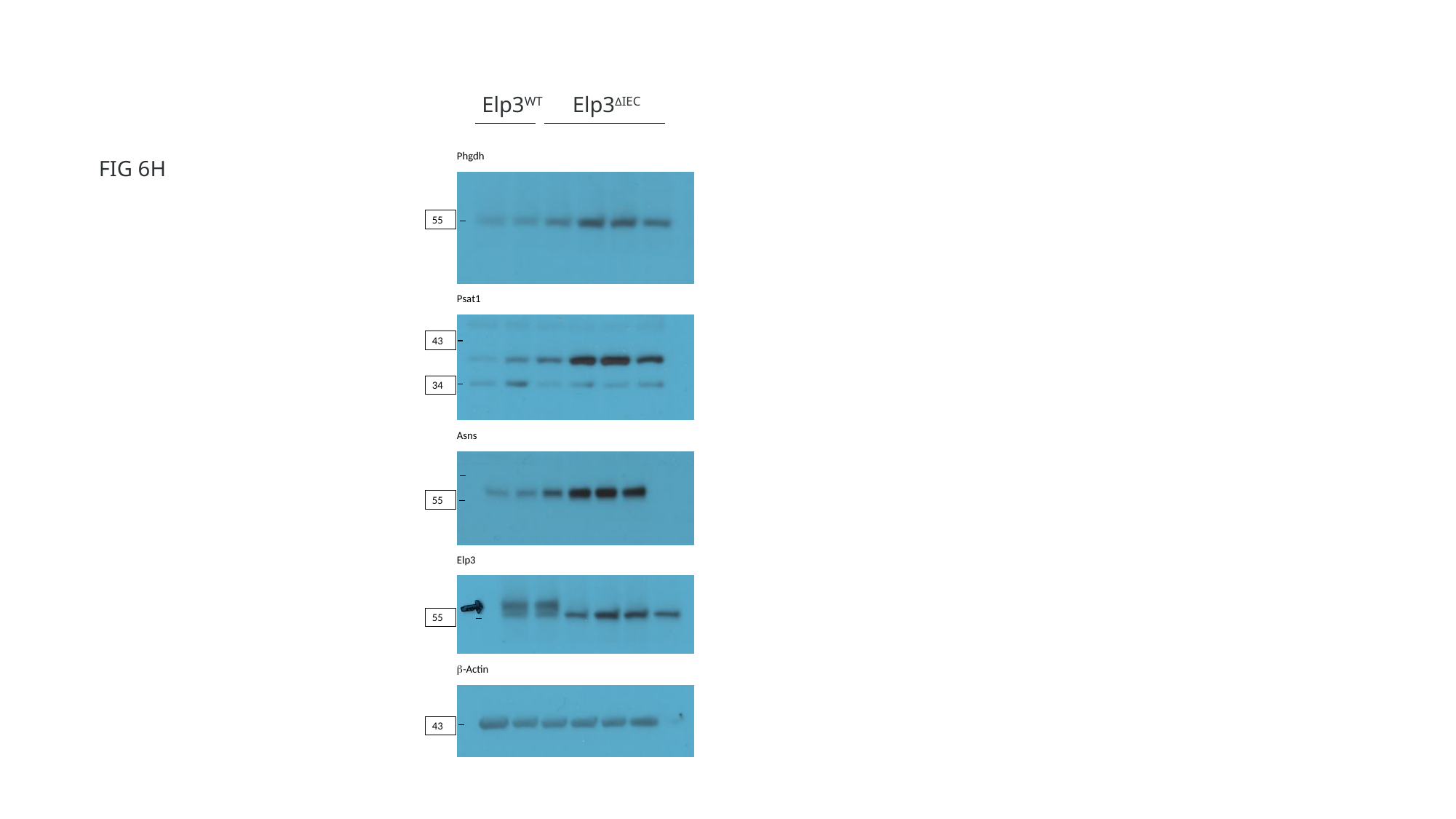

Elp3WT
Elp3ΔIEC
FIG 6H

Supplement: Supplementary file 9 — Source data Fig. 6 [file 44318_2024_184_MOESM9_ESM.zip › Figure 6/6H/WB uncropped gels.pptx]

## Slide 1
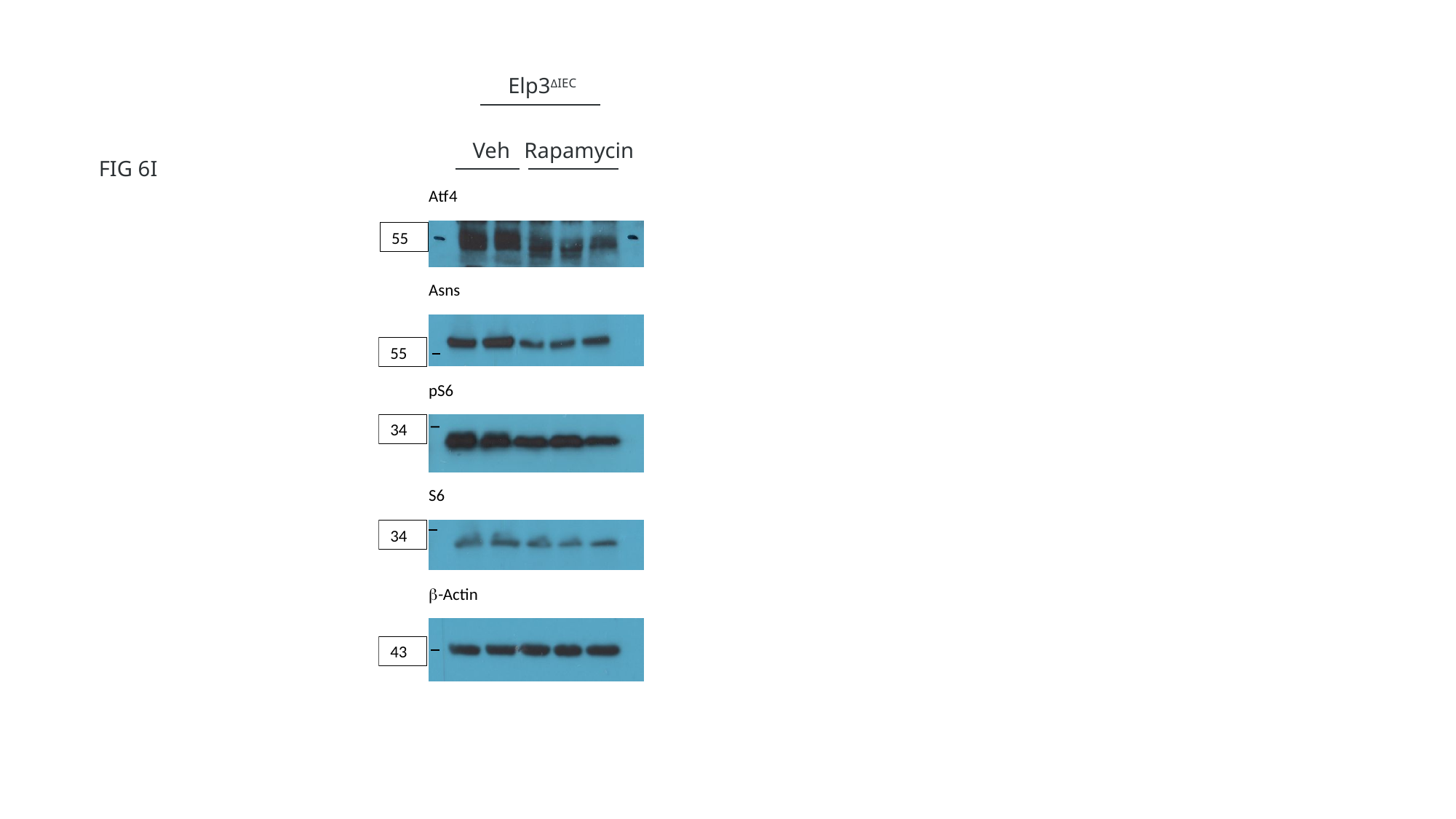

Elp3ΔIEC
Veh
Rapamycin
FIG 6I

Supplement: Supplementary file 9 — Source data Fig. 6 [file 44318_2024_184_MOESM9_ESM.zip › Figure 6/6I/WB uncropped gels.pptx]

## Slide 1
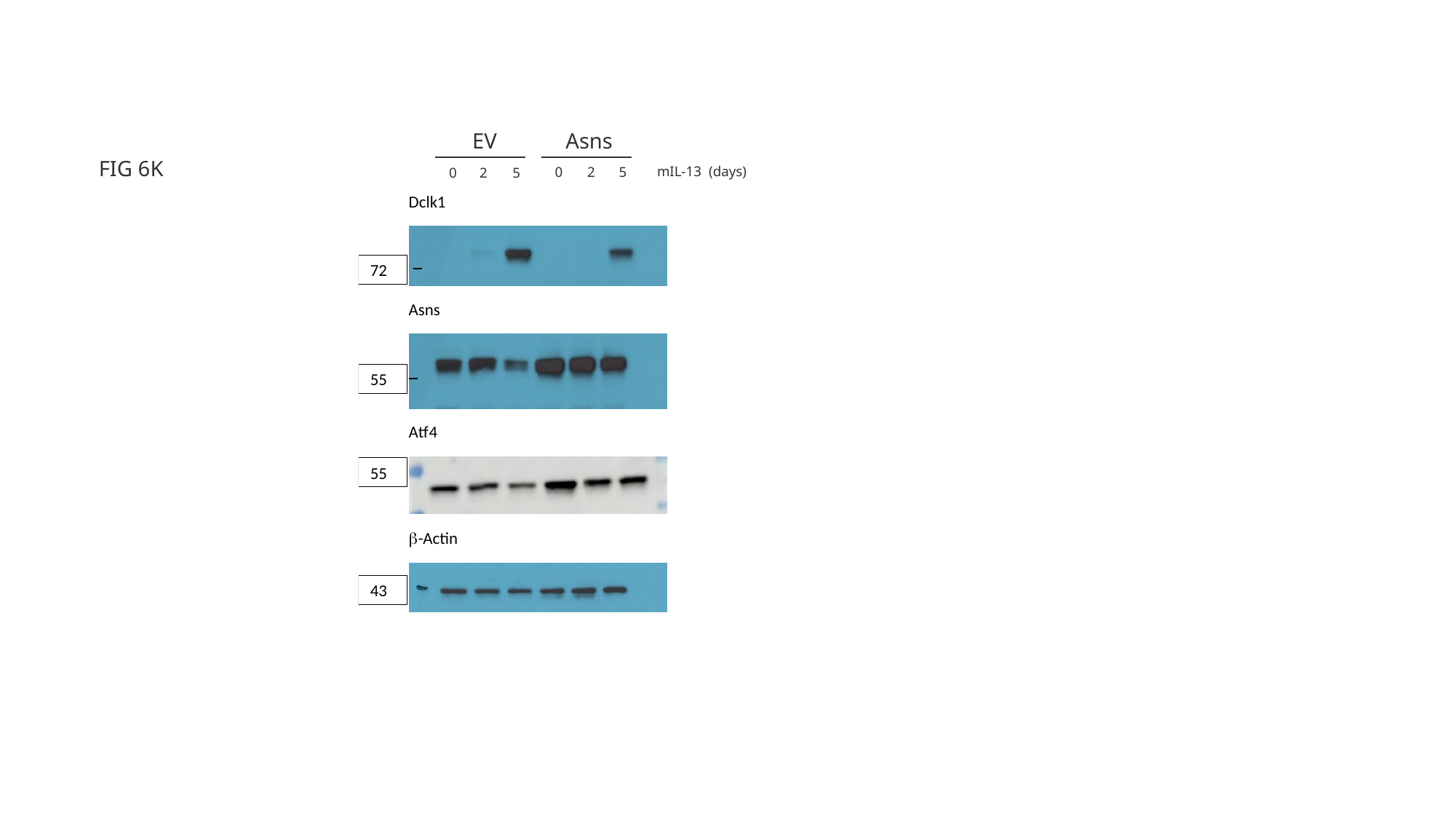

EV
Asns
FIG 6K
mIL-13 (days)
0
2
5
0
2
5

Supplement: Supplementary file 9 — Source data Fig. 6 [file 44318_2024_184_MOESM9_ESM.zip › Figure 6/6J/WB uncropped gels.pptx]

## Slide 1
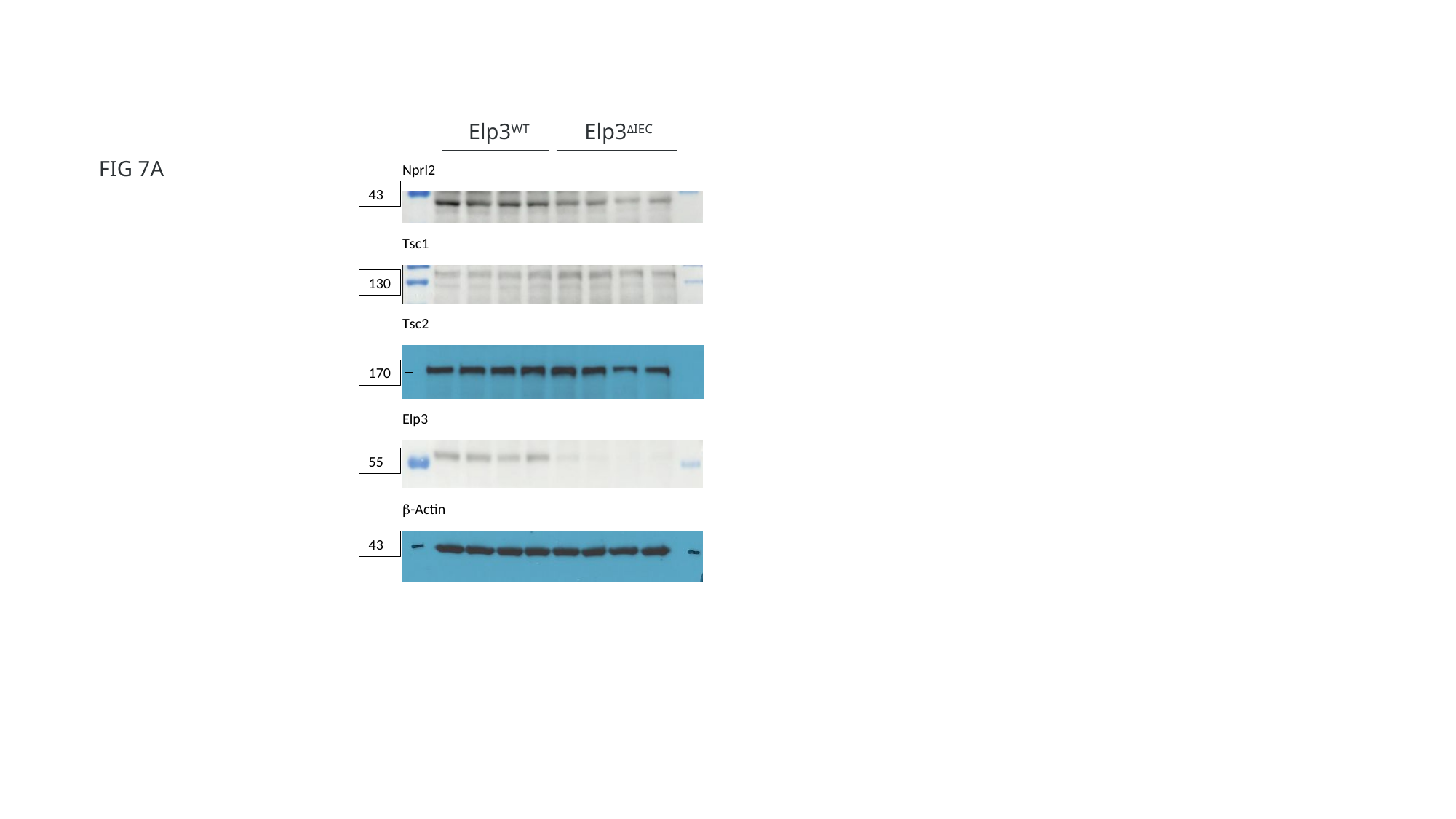

Elp3WT
Elp3ΔIEC
FIG 7A

Supplement: Supplementary file 10 — Source data Fig. 7 [file 44318_2024_184_MOESM10_ESM.zip › Figure 7/7A/WB uncropped gels.pptx]

## Slide 1
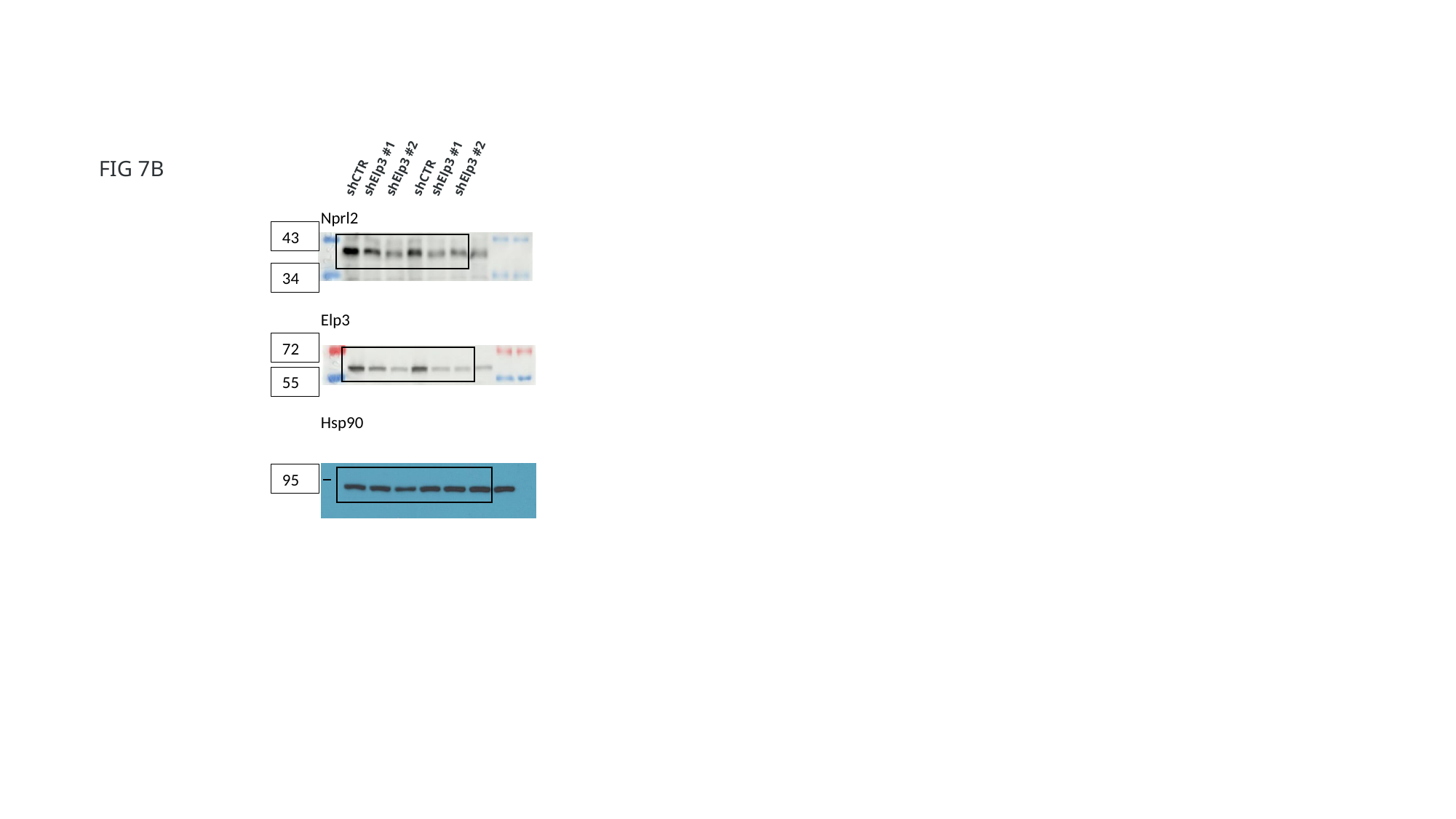

FIG 7B
shElp3 #1
shElp3 #2
shElp3 #1
shElp3 #2
shCTR
shCTR

Supplement: Supplementary file 10 — Source data Fig. 7 [file 44318_2024_184_MOESM10_ESM.zip › Figure 7/7B/WB uncropped gels.pptx]

## Slide 1
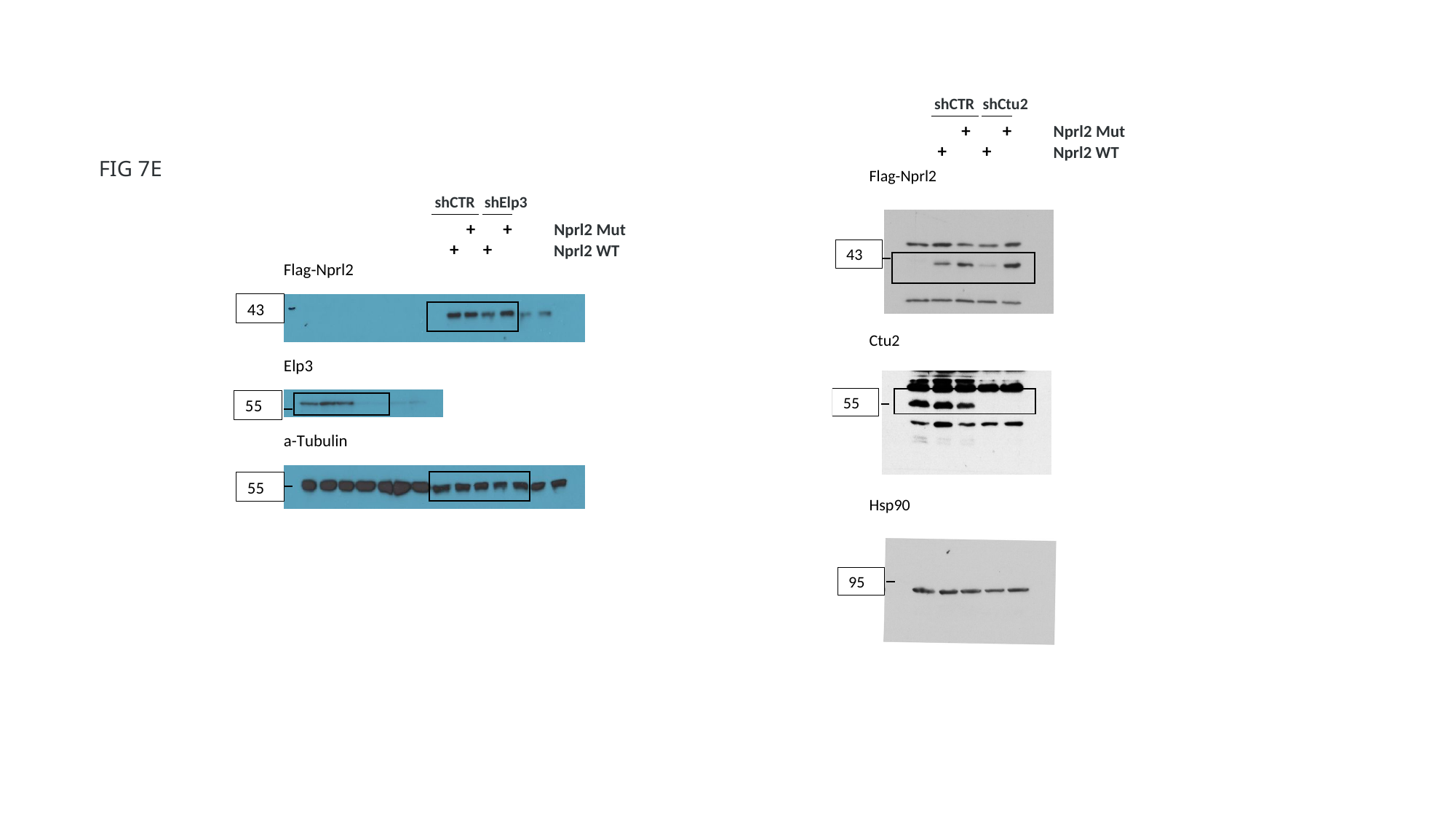

shCTR
shCtu2
+
+
Nprl2 Mut
+
+
Nprl2 WT
FIG 7E
shCTR
shElp3
+
+
Nprl2 Mut
+
+
Nprl2 WT

Supplement: Supplementary file 10 — Source data Fig. 7 [file 44318_2024_184_MOESM10_ESM.zip › Figure 7/7E/WB uncropped gels.pptx]

## Slide 1
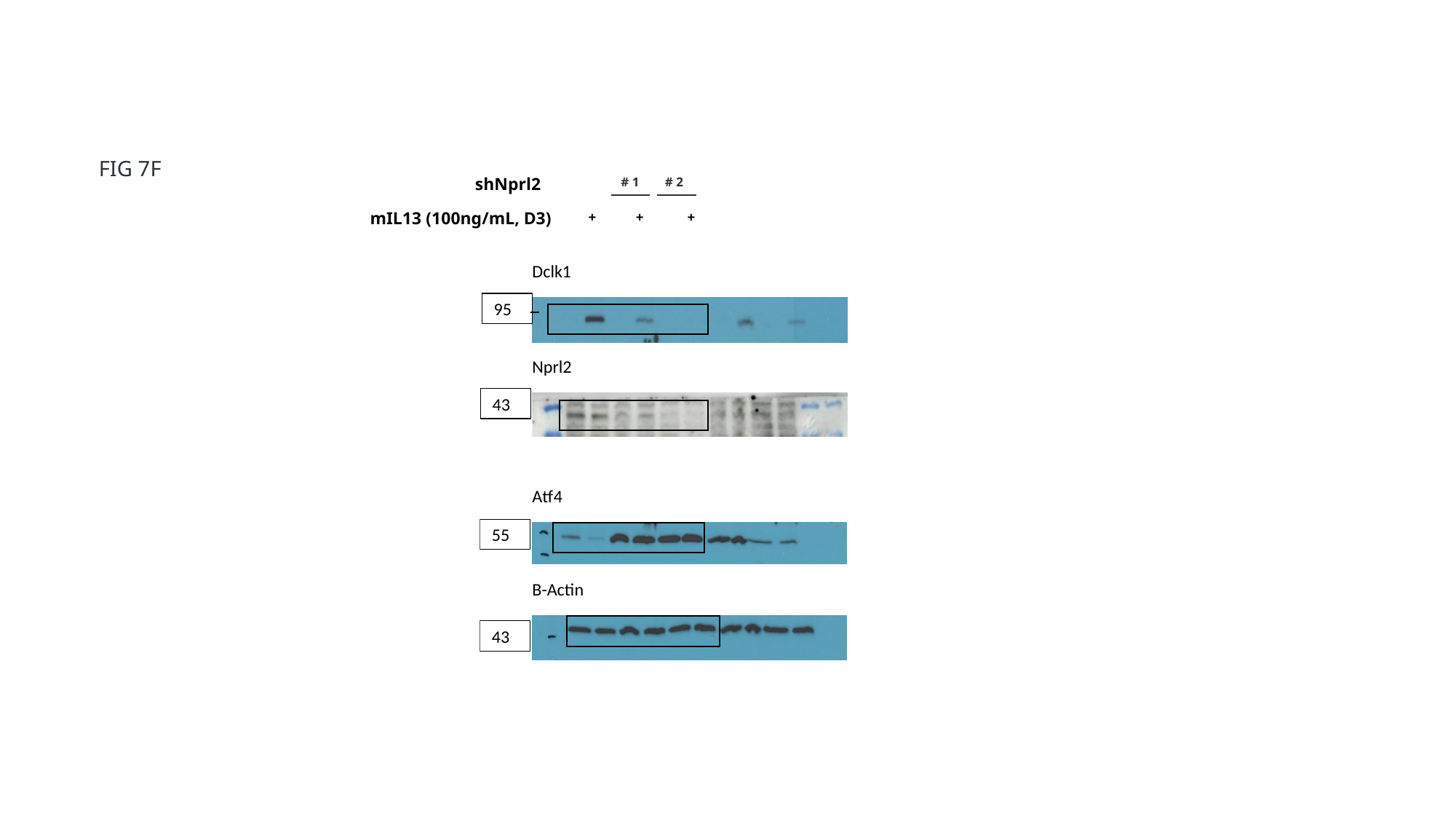

FIG 7F
shNprl2
# 1
# 2
mIL13 (100ng/mL, D3)
+
+
+

Supplement: Supplementary file 10 — Source data Fig. 7 [file 44318_2024_184_MOESM10_ESM.zip › Figure 7/7F/WB uncropped gels.pptx]

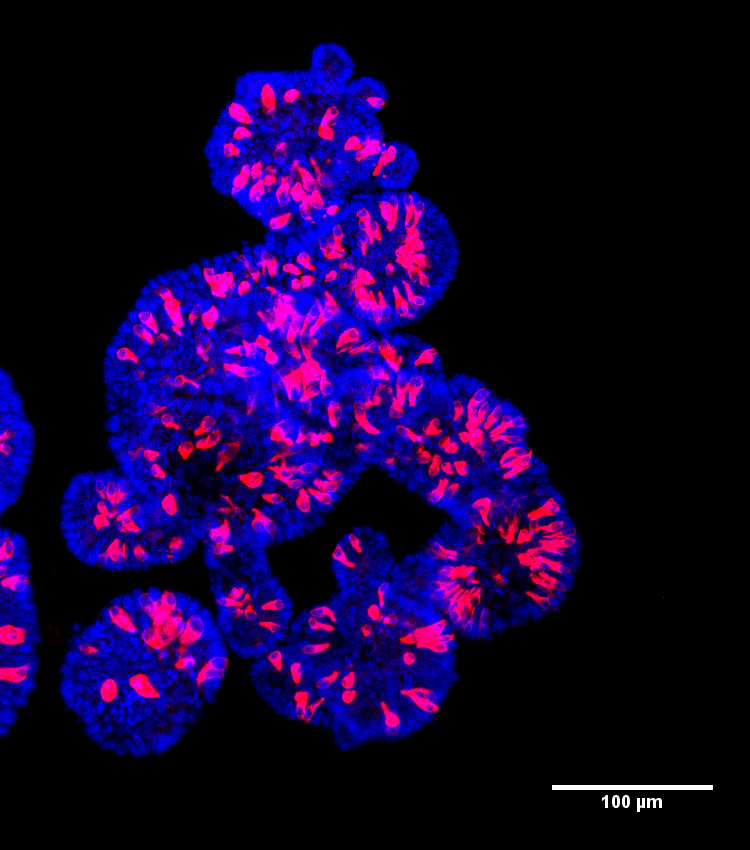

Supplement: Supplementary file 10 — Source data Fig. 7 [file 44318_2024_184_MOESM10_ESM.zip › Figure 7/7G/IF Dclk1 org shCTR IL13.tif]

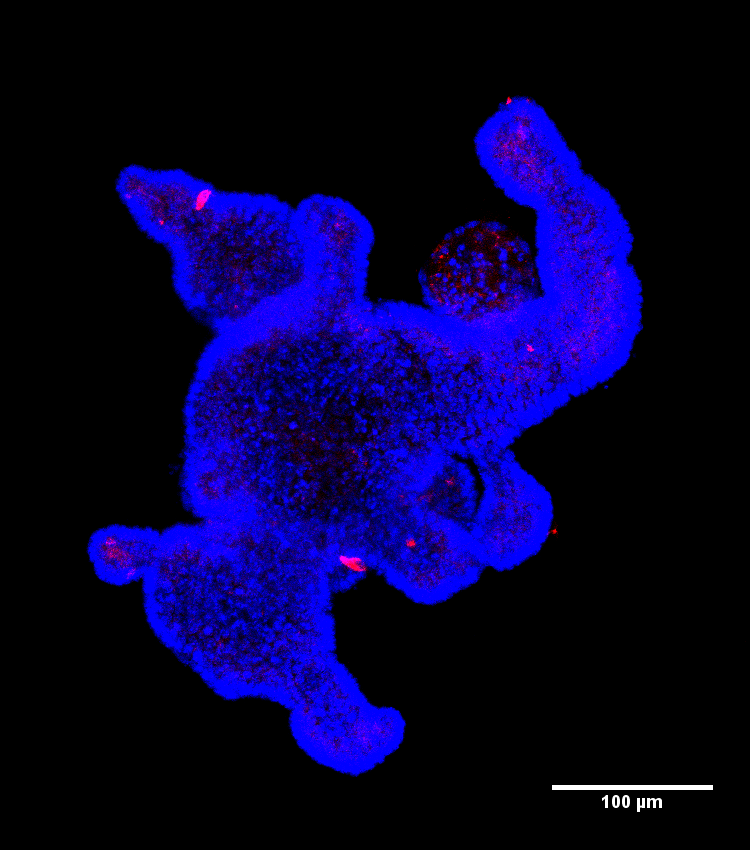

Supplement: Supplementary file 10 — Source data Fig. 7 [file 44318_2024_184_MOESM10_ESM.zip › Figure 7/7G/IF Dclk1 org shCTR PBS.tif]

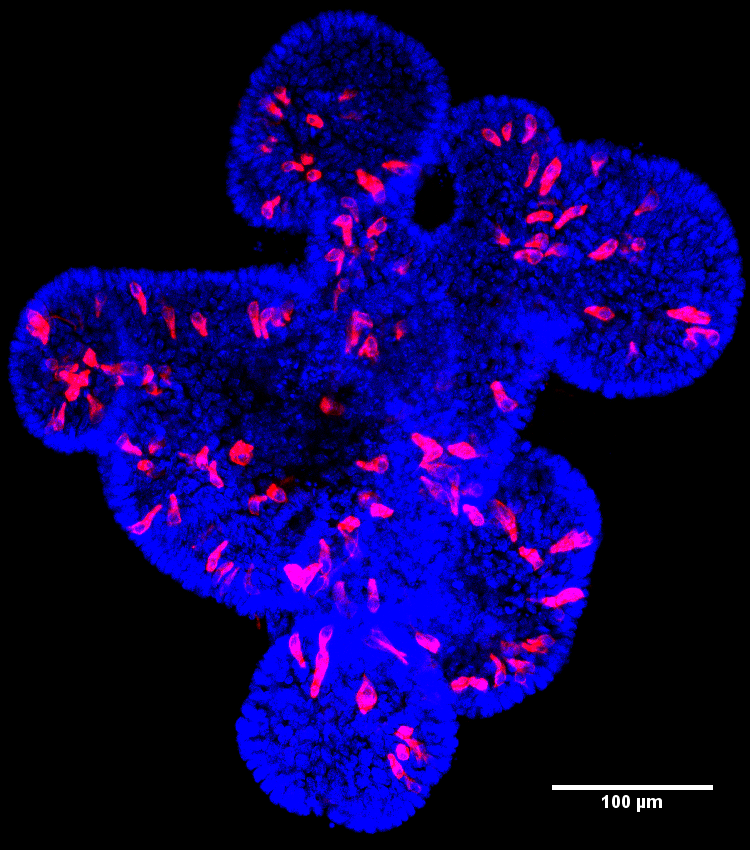

Supplement: Supplementary file 10 — Source data Fig. 7 [file 44318_2024_184_MOESM10_ESM.zip › Figure 7/7G/IF Dclk1 org shNprl2 #1 IL13.tif]

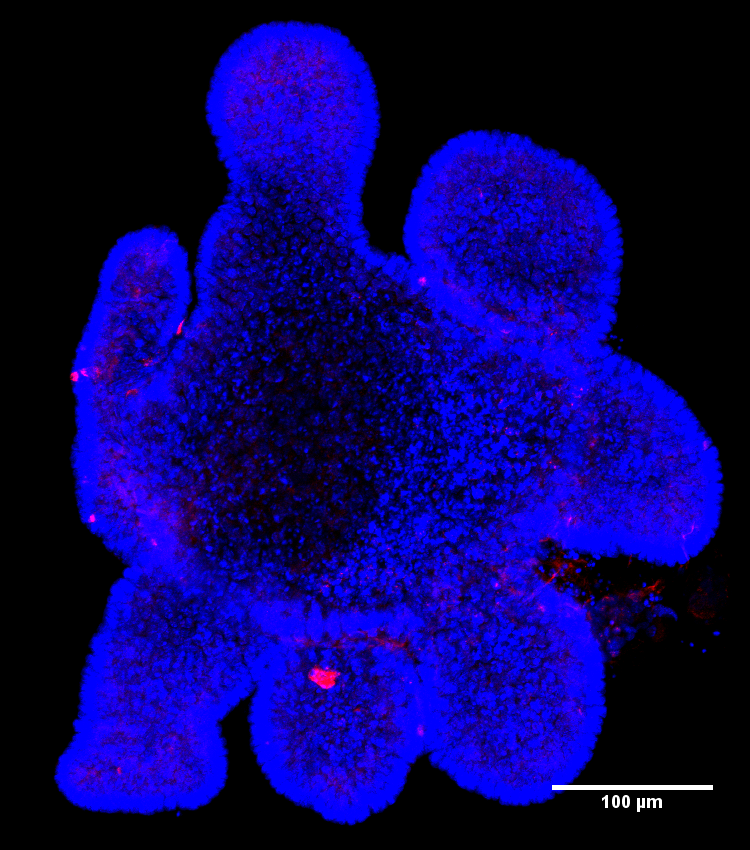

Supplement: Supplementary file 10 — Source data Fig. 7 [file 44318_2024_184_MOESM10_ESM.zip › Figure 7/7G/IF Dclk1 org shNprl2 #1 PBS.tif]

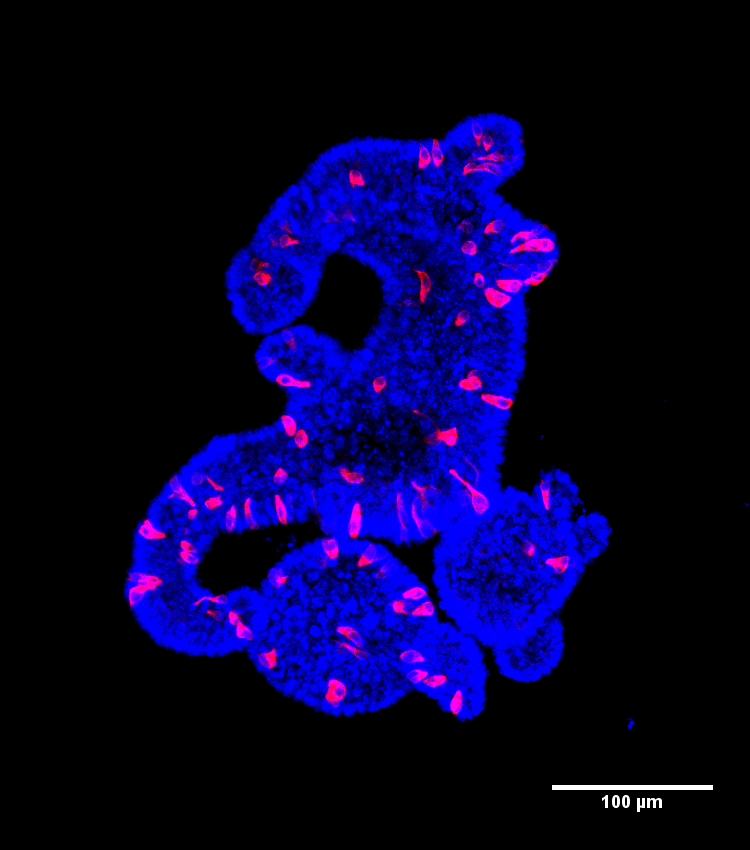

Supplement: Supplementary file 10 — Source data Fig. 7 [file 44318_2024_184_MOESM10_ESM.zip › Figure 7/7G/IF Dclk1 org shNprl2 #2 IL13.tif]

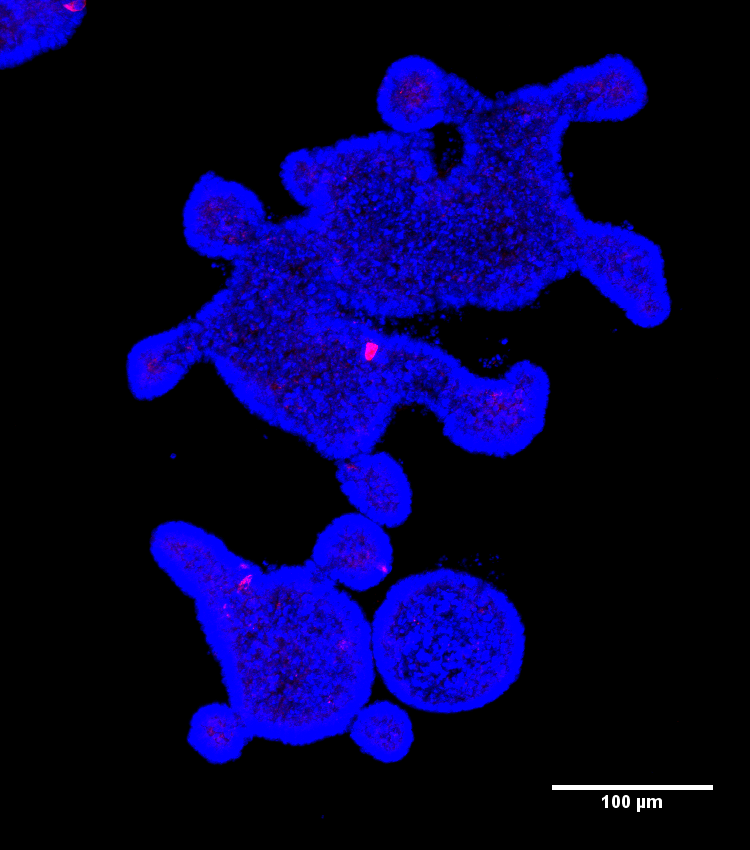

Supplement: Supplementary file 10 — Source data Fig. 7 [file 44318_2024_184_MOESM10_ESM.zip › Figure 7/7G/IF Dclk1 Org shNprl2 #2 PBS.tif]

## Slide 1
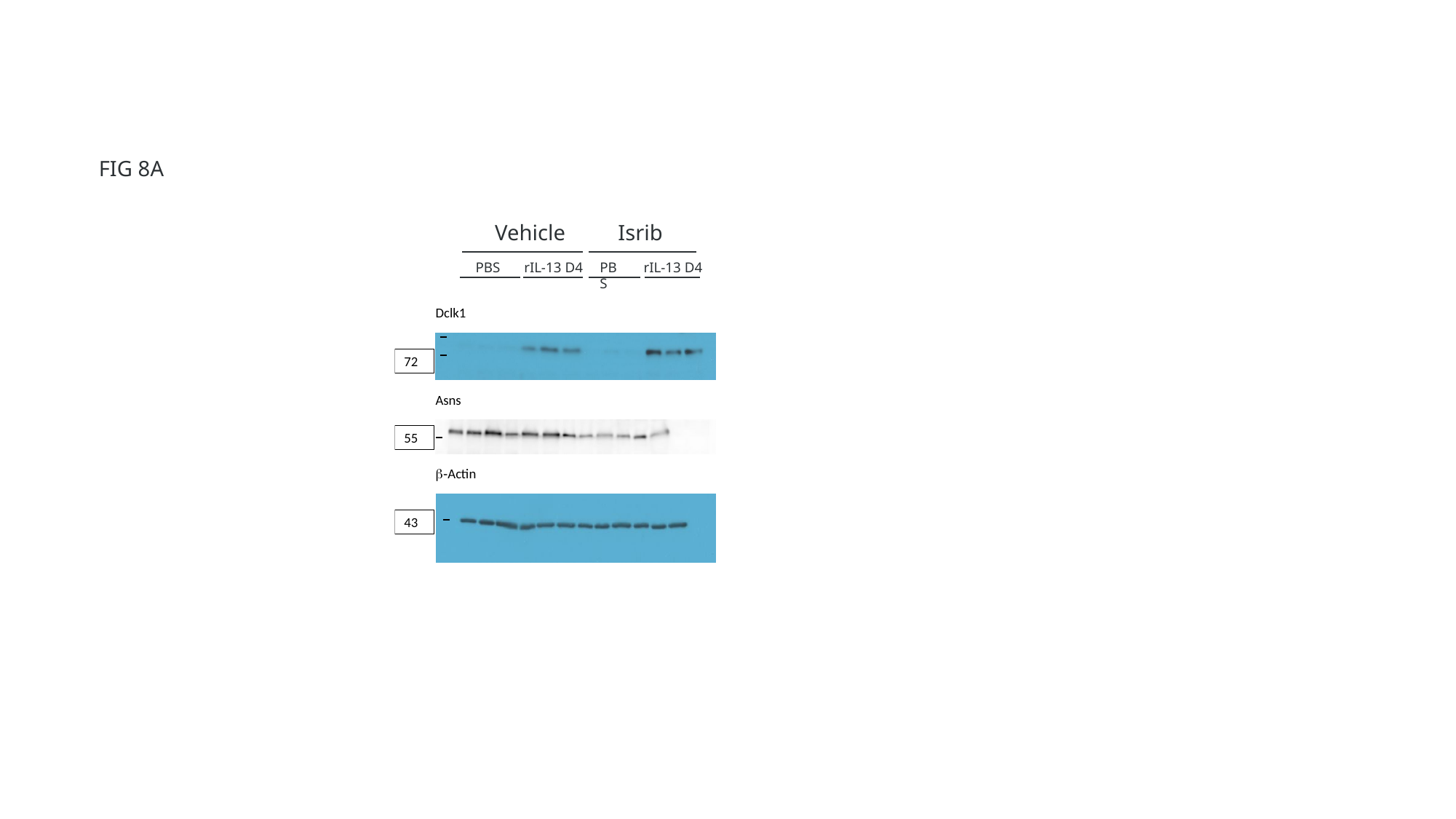

FIG 8A
Vehicle
Isrib
PBS
rIL-13 D4
PBS
rIL-13 D4

Supplement: Supplementary file 11 — Source data Fig. 8 [file 44318_2024_184_MOESM11_ESM.zip › Figure 8/8A/WB uncropped gels.pptx]

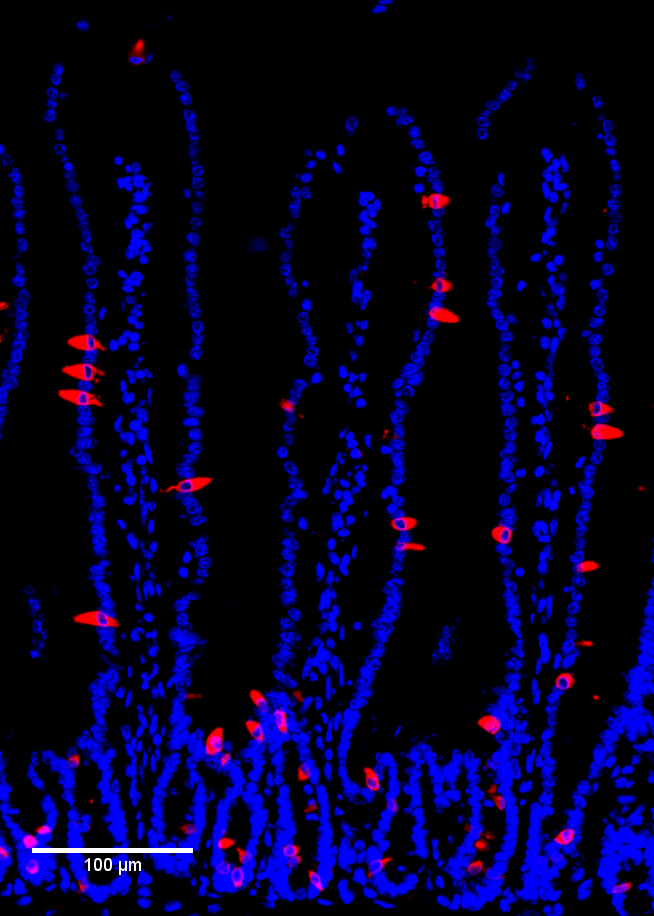

Supplement: Supplementary file 11 — Source data Fig. 8 [file 44318_2024_184_MOESM11_ESM.zip › Figure 8/8B/IF Dclk1 ISRIB Il13.tif]

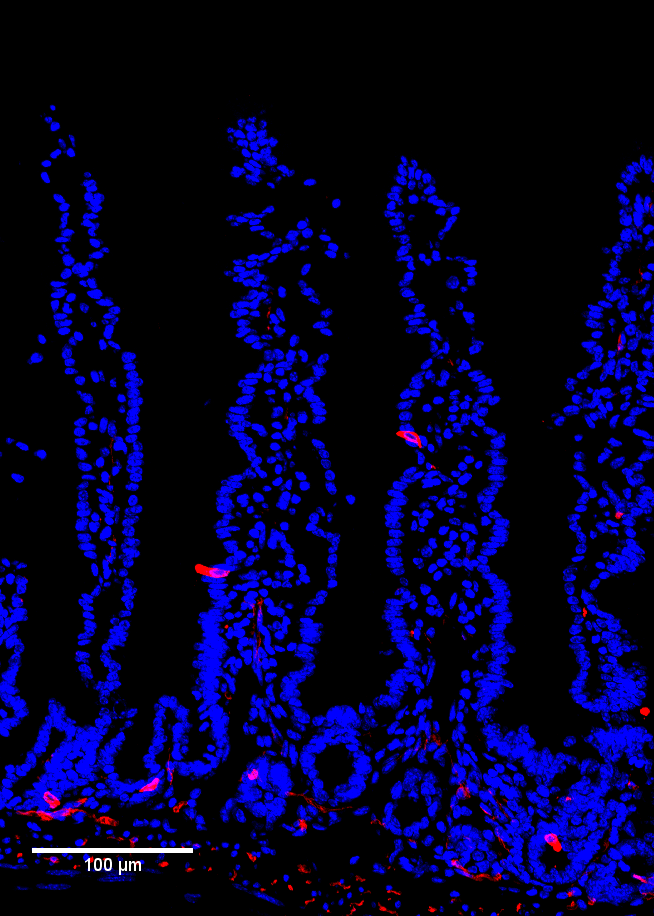

Supplement: Supplementary file 11 — Source data Fig. 8 [file 44318_2024_184_MOESM11_ESM.zip › Figure 8/8B/IF Dclk1 ISRIB PBS.tif]

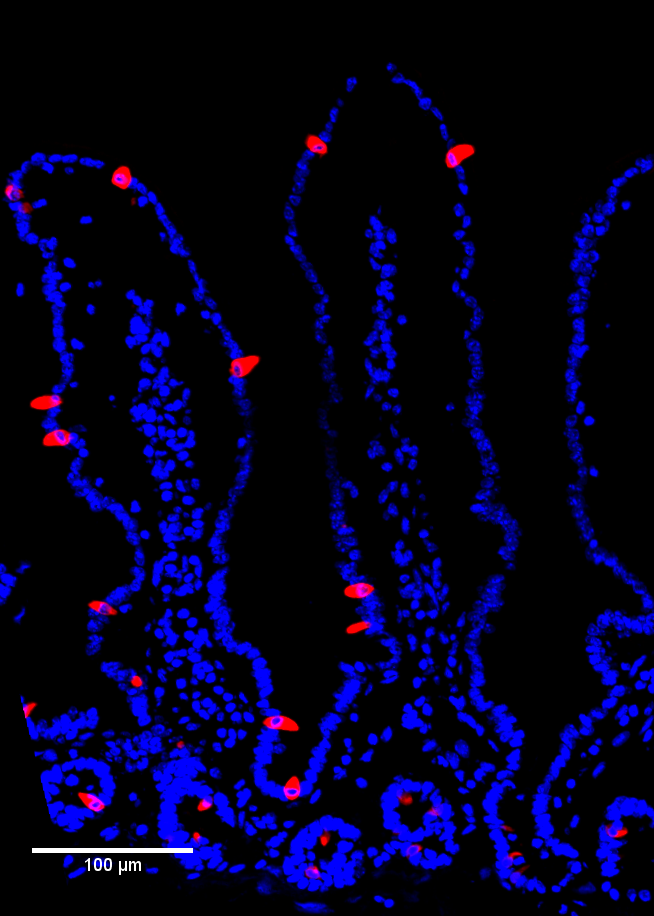

Supplement: Supplementary file 11 — Source data Fig. 8 [file 44318_2024_184_MOESM11_ESM.zip › Figure 8/8B/IF Dclk1 VEH IL13.tif]

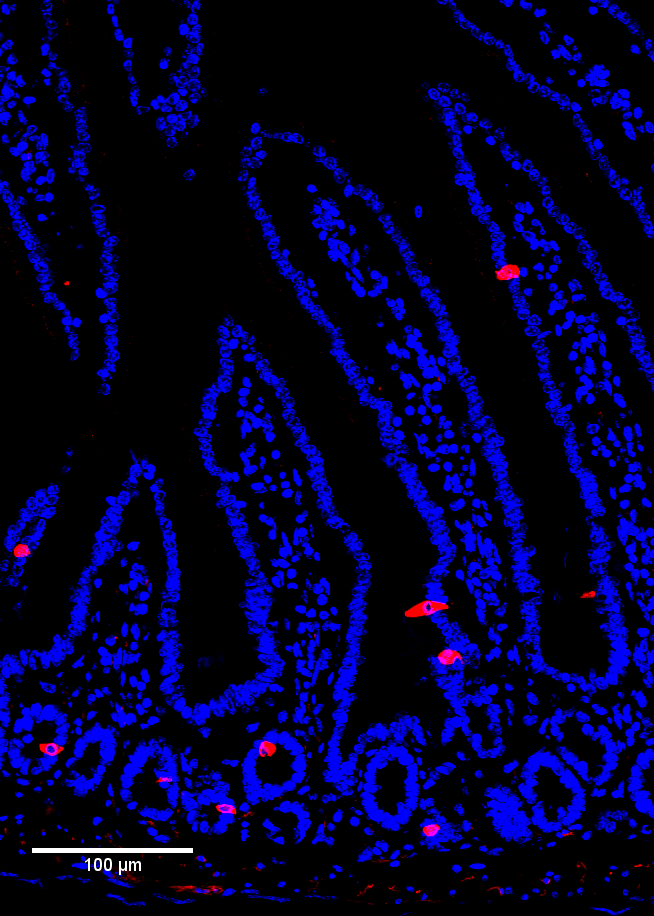

Supplement: Supplementary file 11 — Source data Fig. 8 [file 44318_2024_184_MOESM11_ESM.zip › Figure 8/8B/IF Dclk1 VEH PBS.tif]

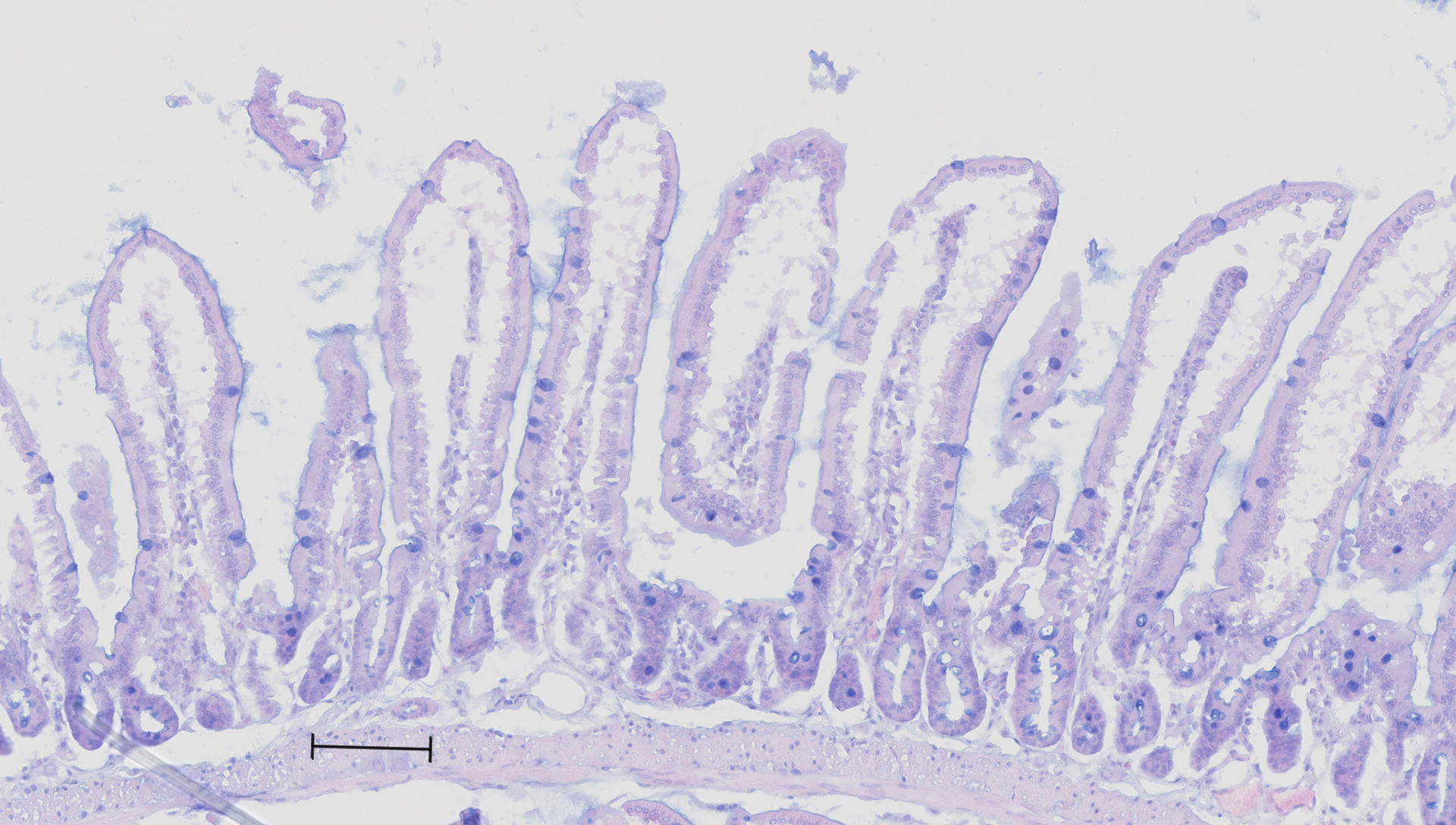

Supplement: Supplementary file 11 — Source data Fig. 8 [file 44318_2024_184_MOESM11_ESM.zip › Figure 8/8C/IHC Alcian Blue Isrib IL13.jpg]

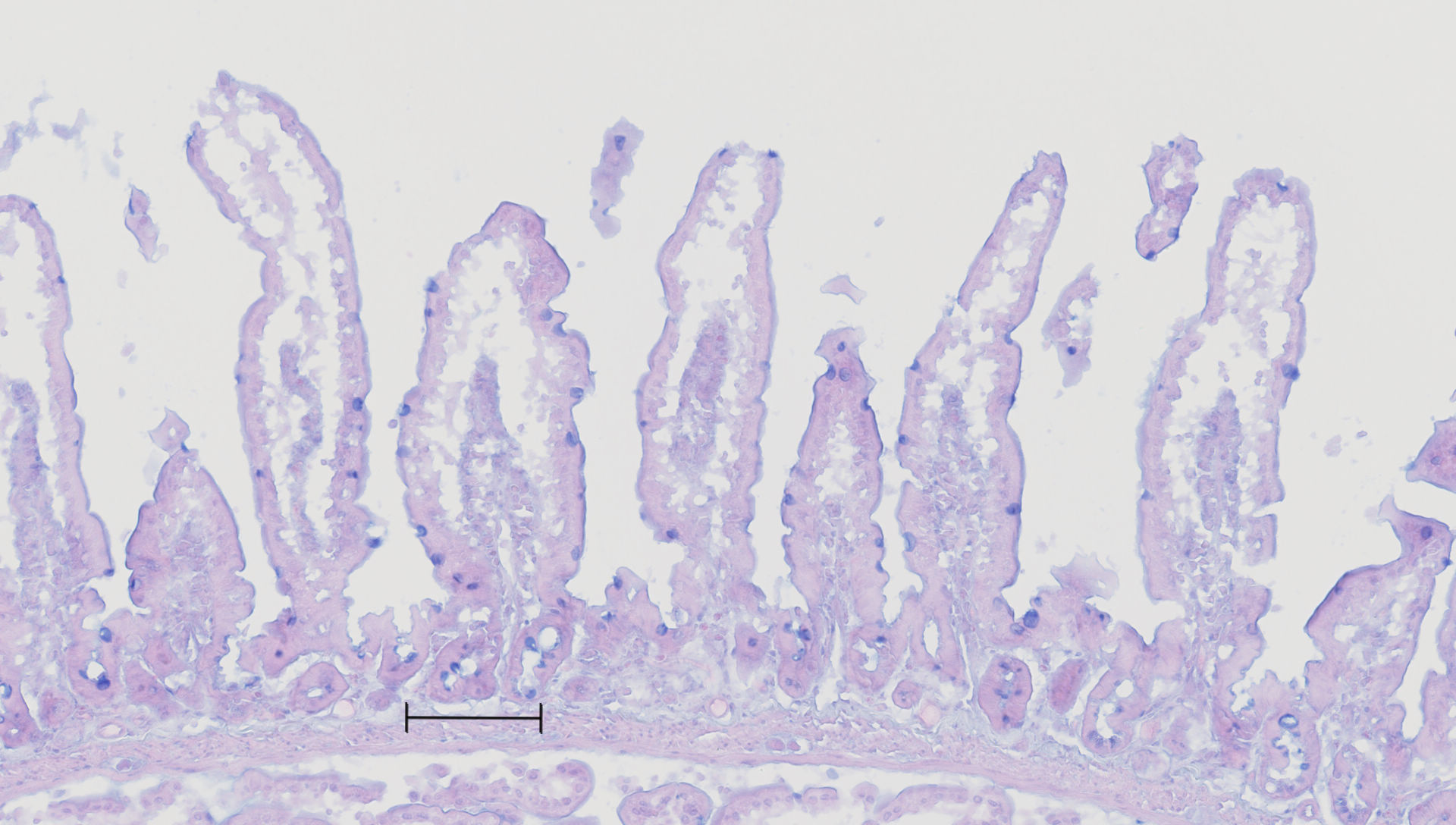

Supplement: Supplementary file 11 — Source data Fig. 8 [file 44318_2024_184_MOESM11_ESM.zip › Figure 8/8C/IHC Alcian Blue Isrib PBS.jpg]

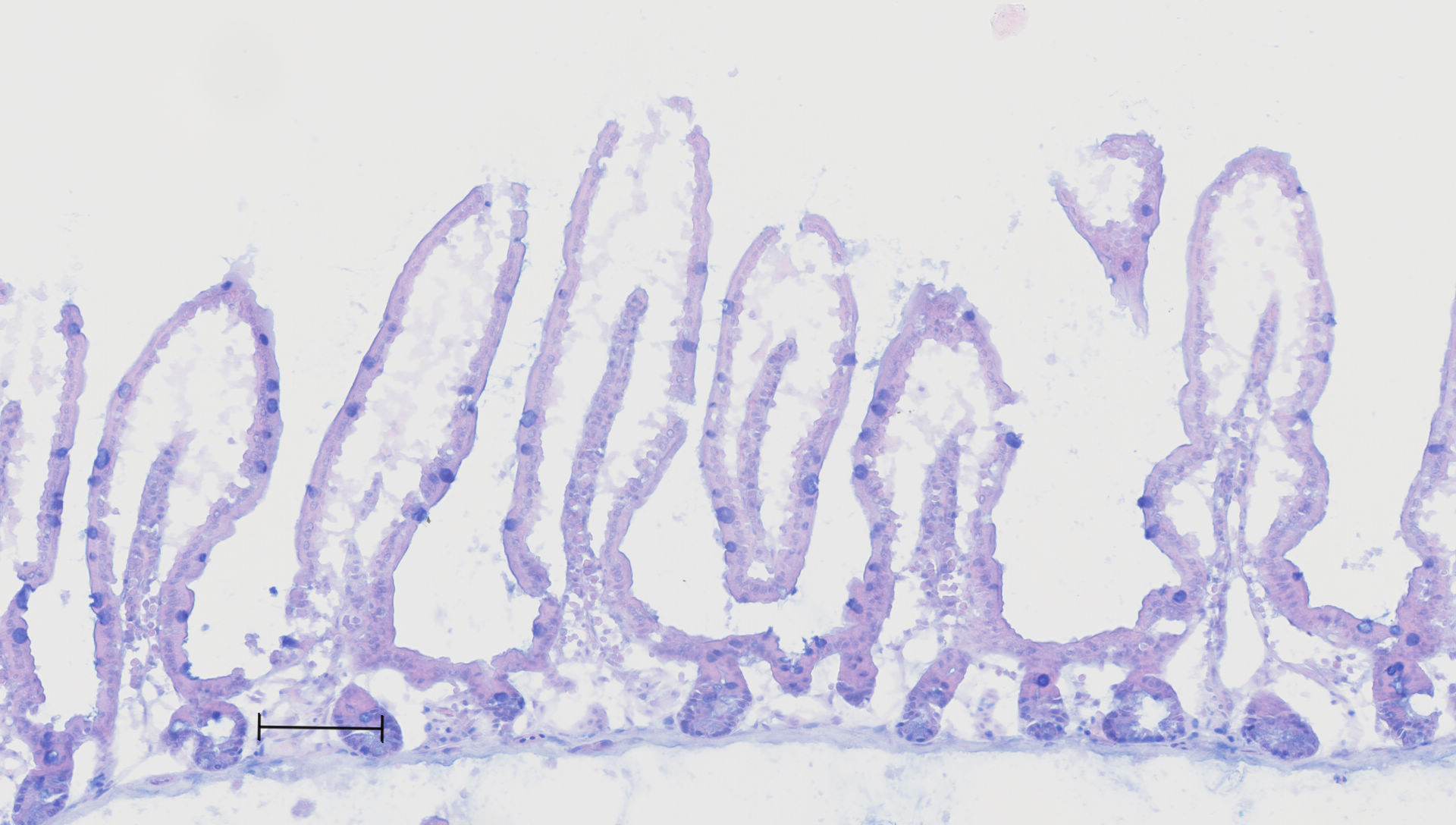

Supplement: Supplementary file 11 — Source data Fig. 8 [file 44318_2024_184_MOESM11_ESM.zip › Figure 8/8C/IHC Alcian Blue Veh IL13.jpg]

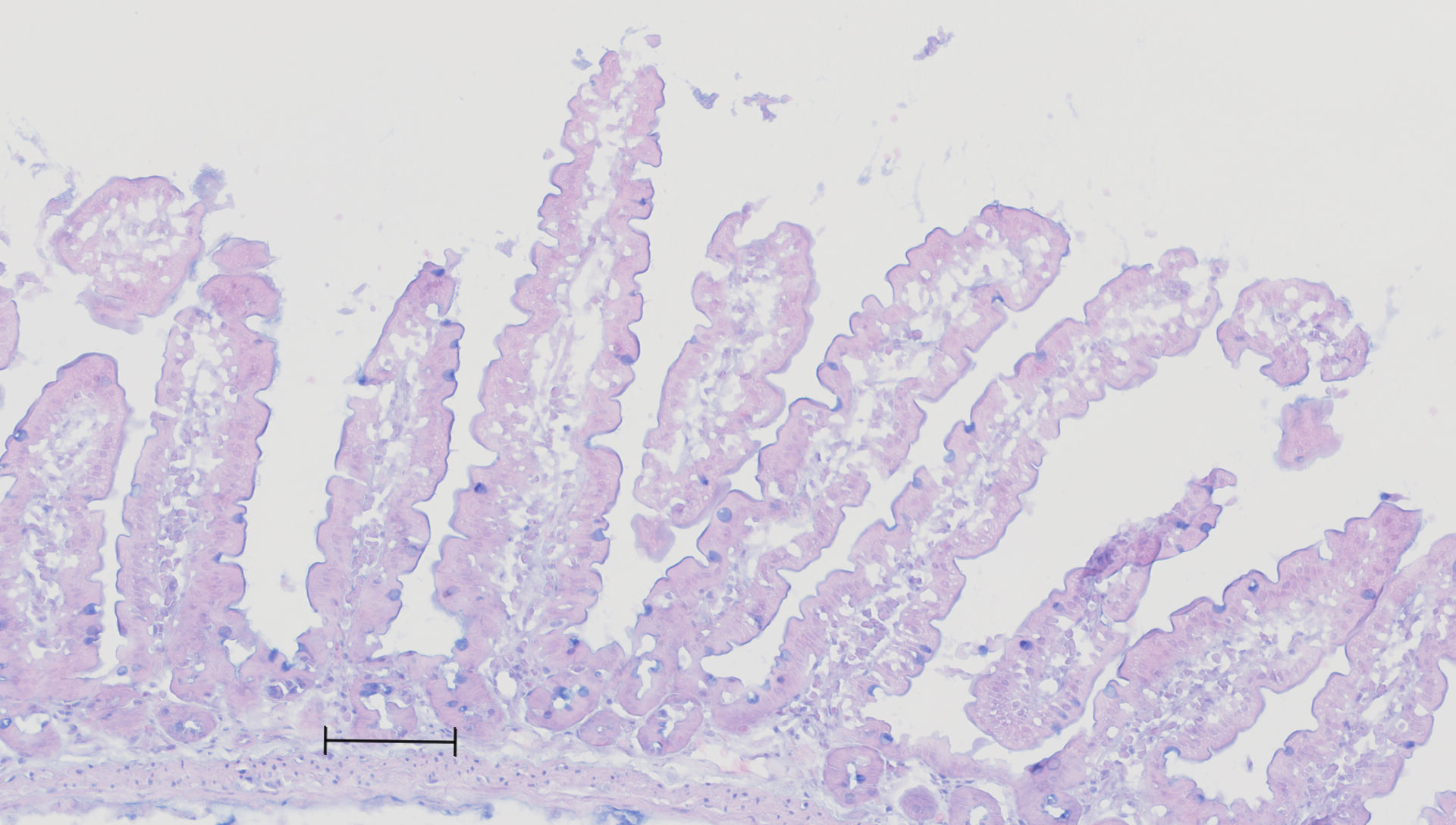

Supplement: Supplementary file 11 — Source data Fig. 8 [file 44318_2024_184_MOESM11_ESM.zip › Figure 8/8C/IHC Alcian Blue Veh PBS.jpg]

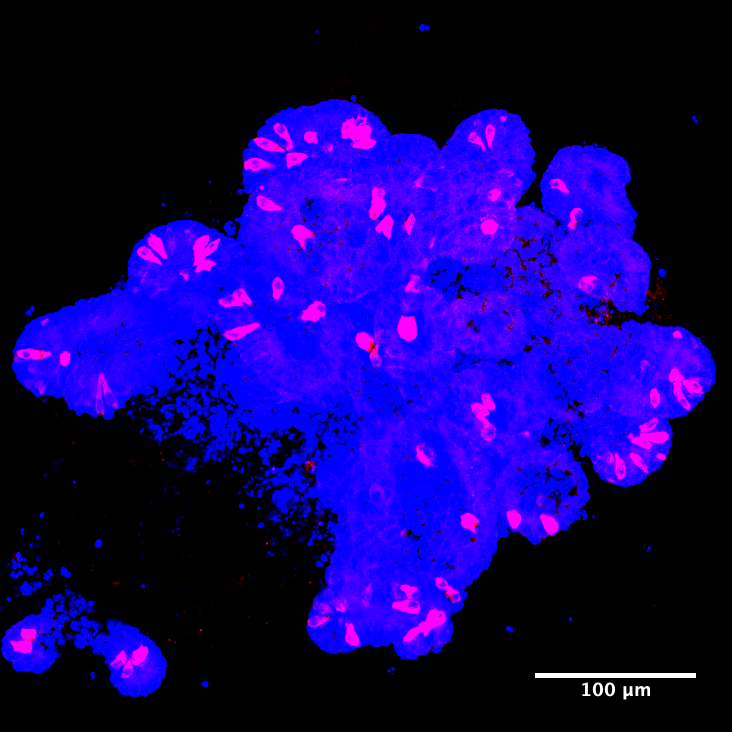

Supplement: Supplementary file 11 — Source data Fig. 8 [file 44318_2024_184_MOESM11_ESM.zip › Figure 8/8D/IF Dclk1 Organoids IL13.tif]

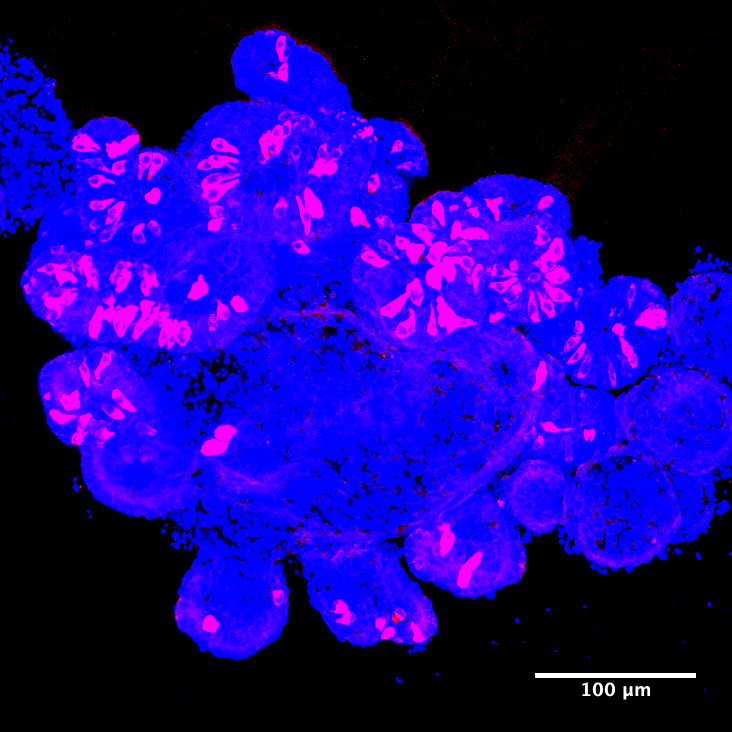

Supplement: Supplementary file 11 — Source data Fig. 8 [file 44318_2024_184_MOESM11_ESM.zip › Figure 8/8D/IF Dclk1 Organoids ISRIB + IL13.tif]

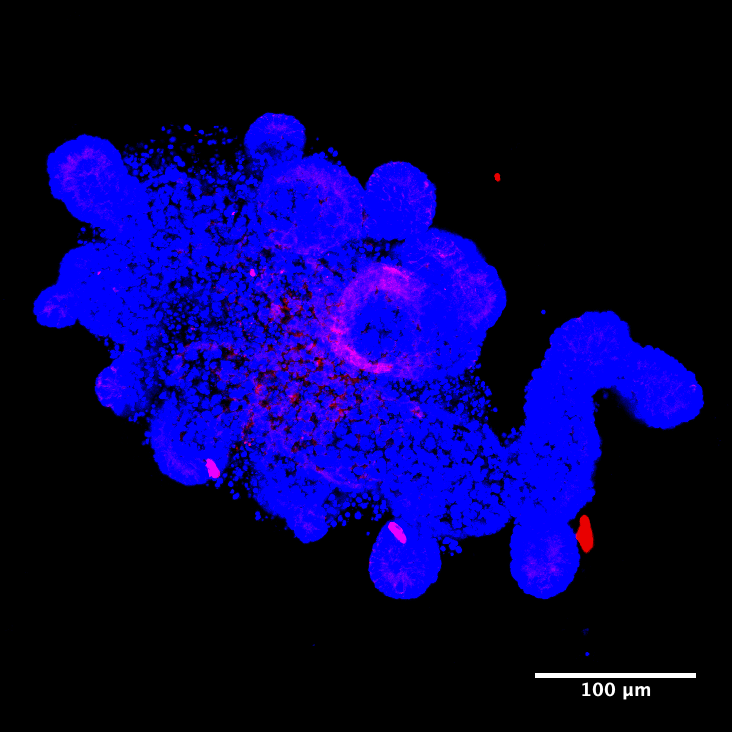

Supplement: Supplementary file 11 — Source data Fig. 8 [file 44318_2024_184_MOESM11_ESM.zip › Figure 8/8D/IF Dclk1 Organoids PBS.tif]

## Slide 1
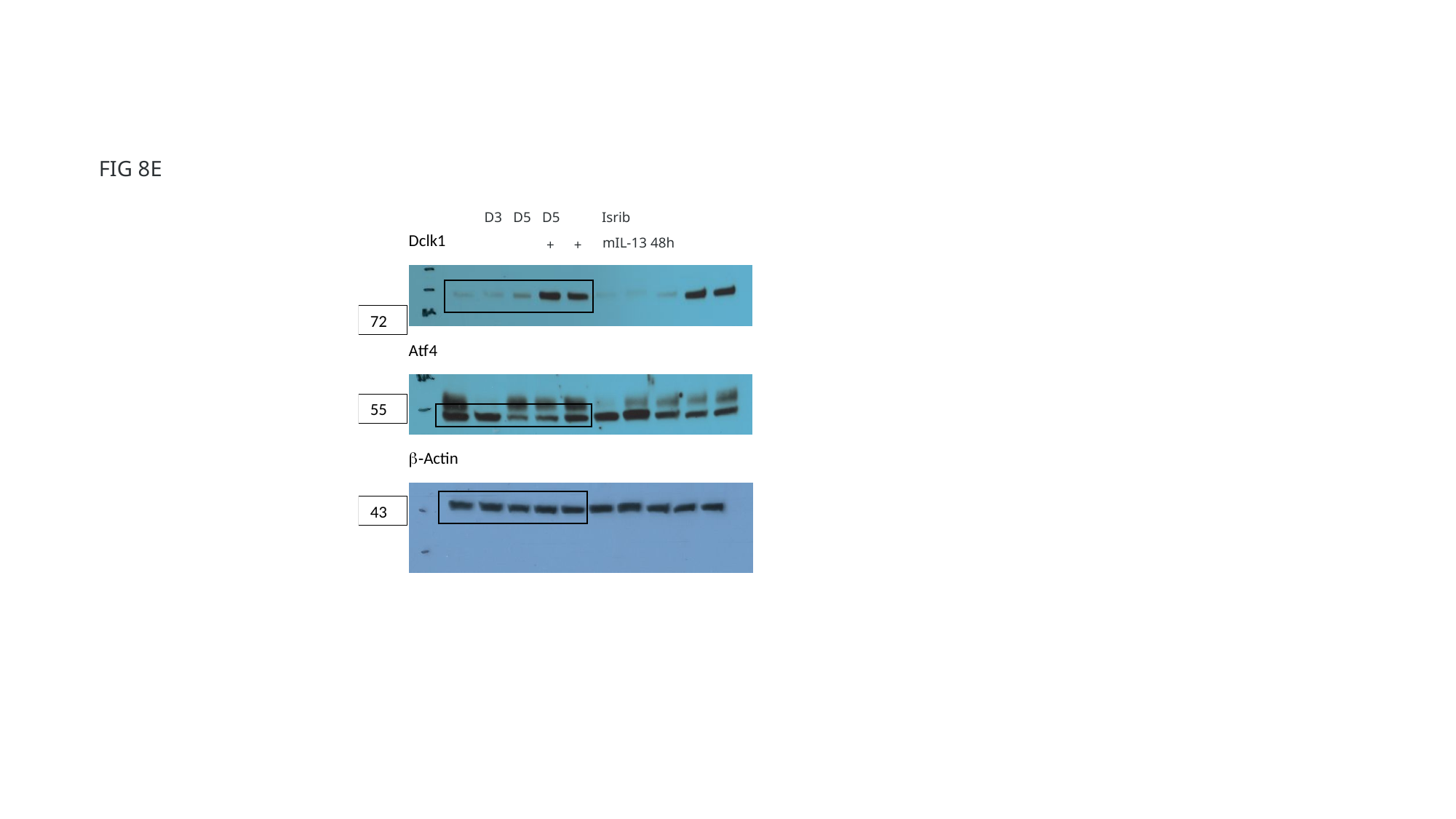

FIG 8E
D3
D5
D5
Isrib
mIL-13 48h
+
+

Supplement: Supplementary file 11 — Source data Fig. 8 [file 44318_2024_184_MOESM11_ESM.zip › Figure 8/8E/WB uncropped gels.pptx]

## Slide 1
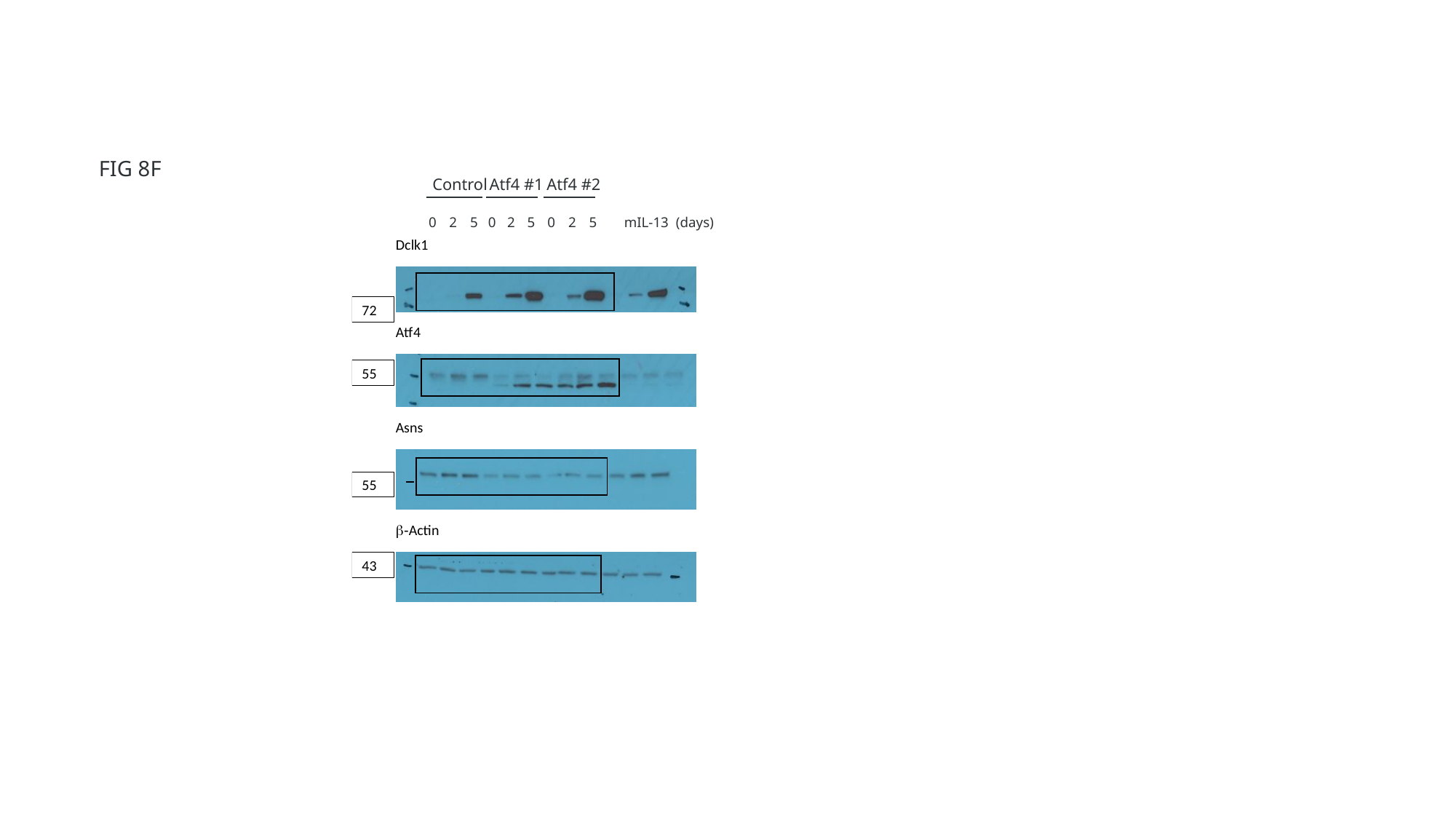

FIG 8F
Control
Atf4 #1
Atf4 #2
mIL-13 (days)
0
2
5
0
2
5
0
2
5

Supplement: Supplementary file 11 — Source data Fig. 8 [file 44318_2024_184_MOESM11_ESM.zip › Figure 8/8F/WB uncropped gels.pptx]

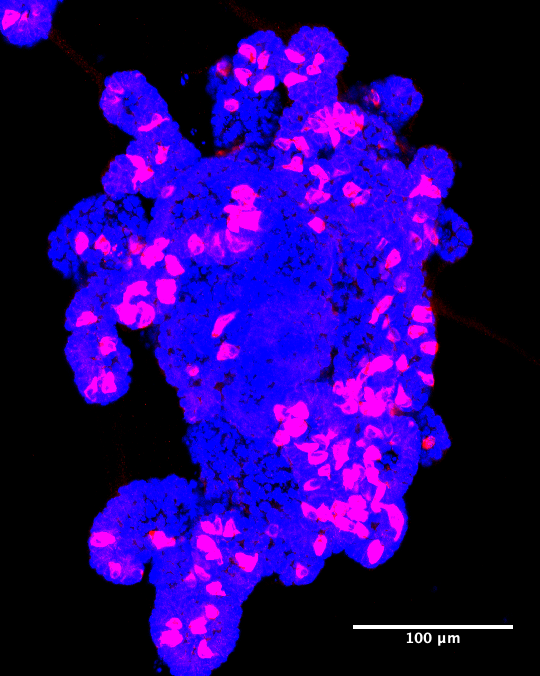

Supplement: Supplementary file 11 — Source data Fig. 8 [file 44318_2024_184_MOESM11_ESM.zip › Figure 8/8G/Dclk1 IF shAtf4_1 IL13.tif]

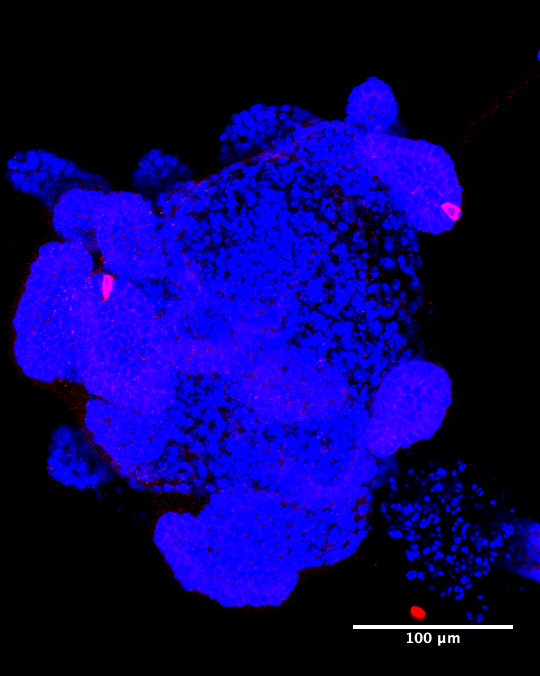

Supplement: Supplementary file 11 — Source data Fig. 8 [file 44318_2024_184_MOESM11_ESM.zip › Figure 8/8G/Dclk1 IF shAtf4_1 PBS.tif]

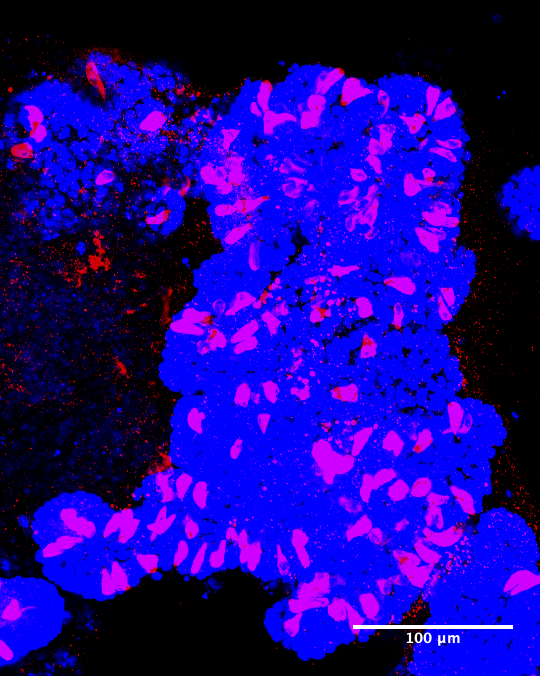

Supplement: Supplementary file 11 — Source data Fig. 8 [file 44318_2024_184_MOESM11_ESM.zip › Figure 8/8G/Dclk1 IF shATF4_2 IL13.tif]

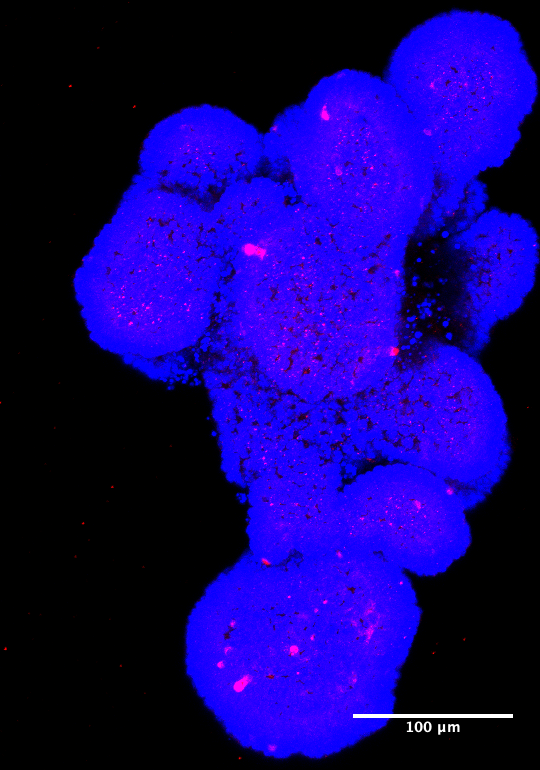

Supplement: Supplementary file 11 — Source data Fig. 8 [file 44318_2024_184_MOESM11_ESM.zip › Figure 8/8G/Dclk1 IF shAtf4_2 PBS.tif]

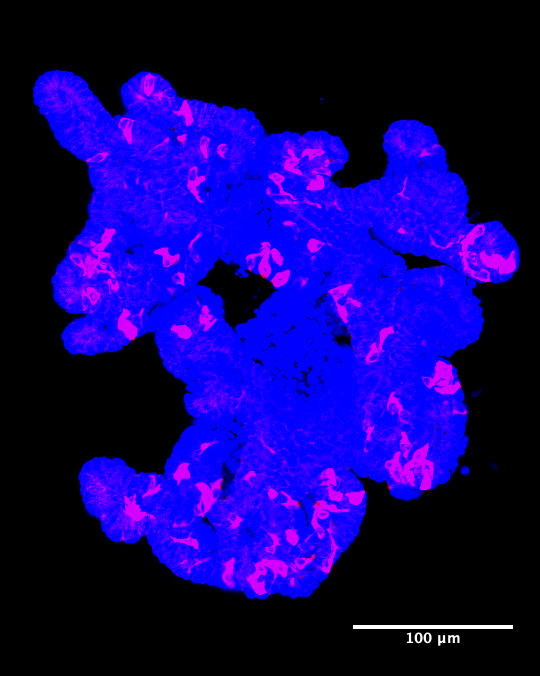

Supplement: Supplementary file 11 — Source data Fig. 8 [file 44318_2024_184_MOESM11_ESM.zip › Figure 8/8G/Dclk1 IF shCTR IL13.tif]

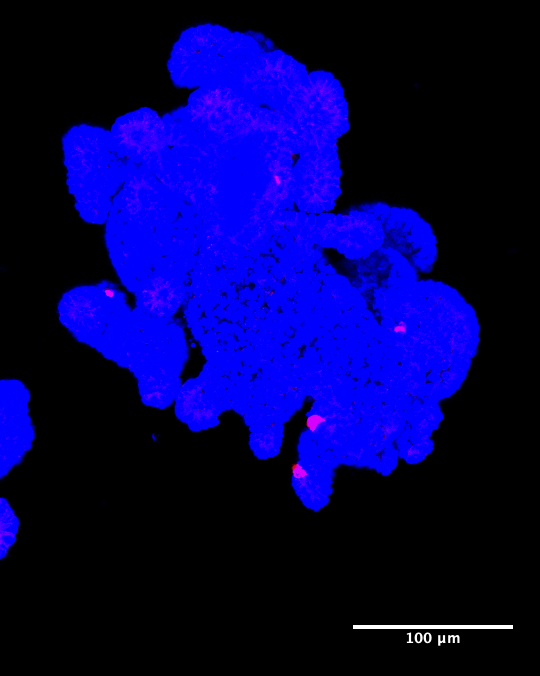

Supplement: Supplementary file 11 — Source data Fig. 8 [file 44318_2024_184_MOESM11_ESM.zip › Figure 8/8G/Dclk1 IF shCTR PBS.tif]

## Slide 1
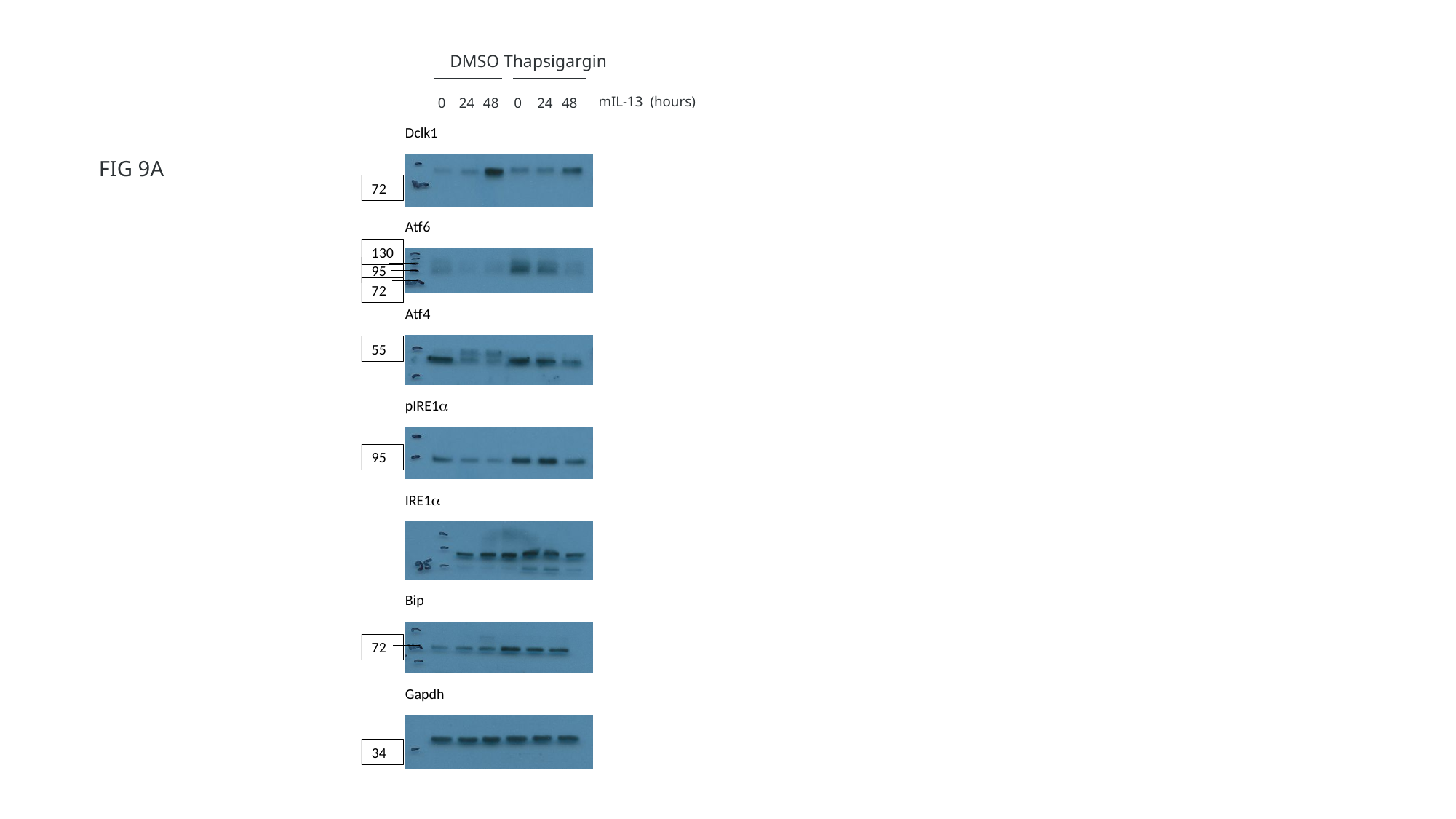

DMSO
Thapsigargin
mIL-13 (hours)
0
24
48
0
24
48
FIG 9A

Supplement: Supplementary file 12 — Source data Fig. 9 [file 44318_2024_184_MOESM12_ESM.zip › Figure 9/9A/WB uncropped gels.pptx]

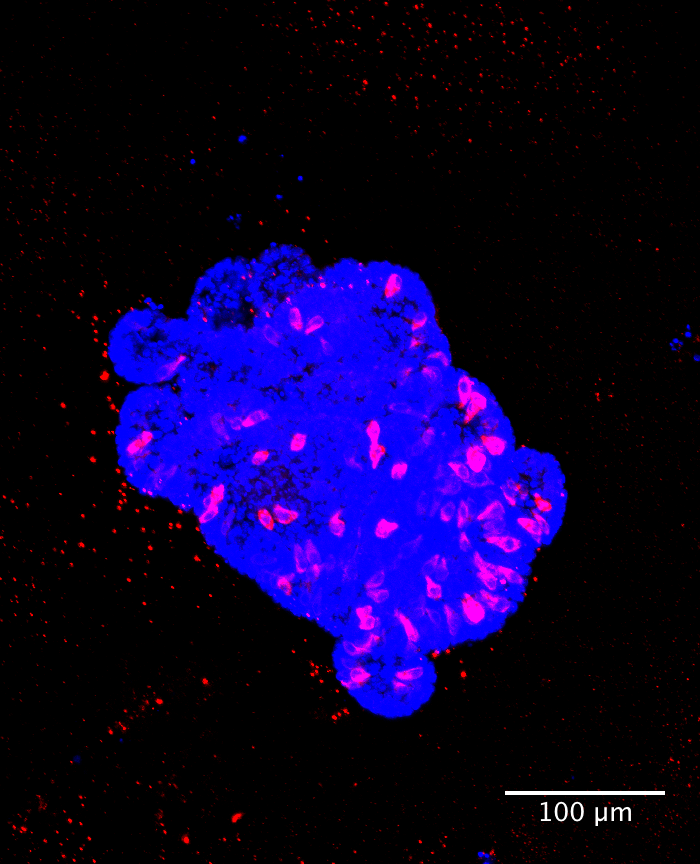

Supplement: Supplementary file 12 — Source data Fig. 9 [file 44318_2024_184_MOESM12_ESM.zip › Figure 9/9B/Dclk1 IF organoid + DMSO + IL13.tif]

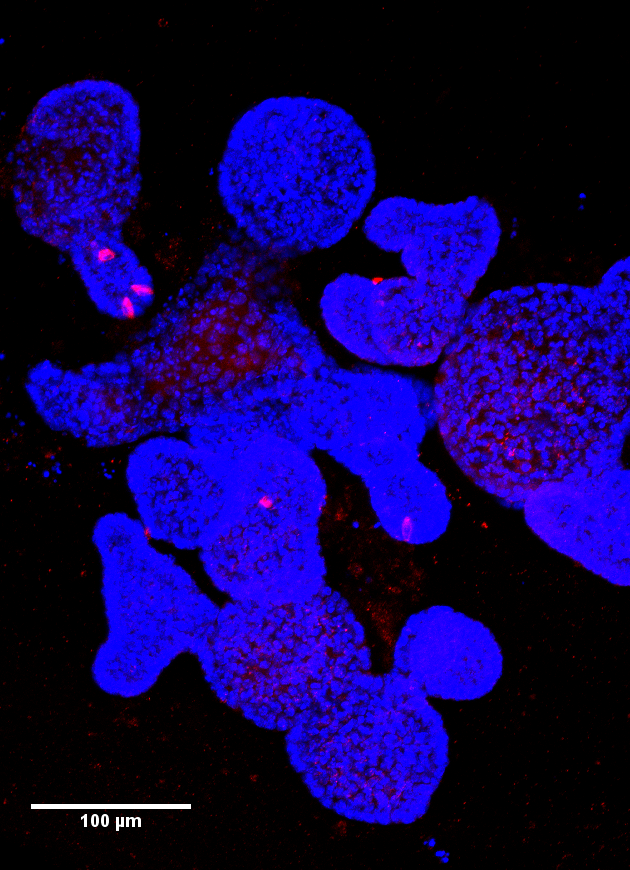

Supplement: Supplementary file 12 — Source data Fig. 9 [file 44318_2024_184_MOESM12_ESM.zip › Figure 9/9B/Dclk1 IF organoid + DMSO.tif]

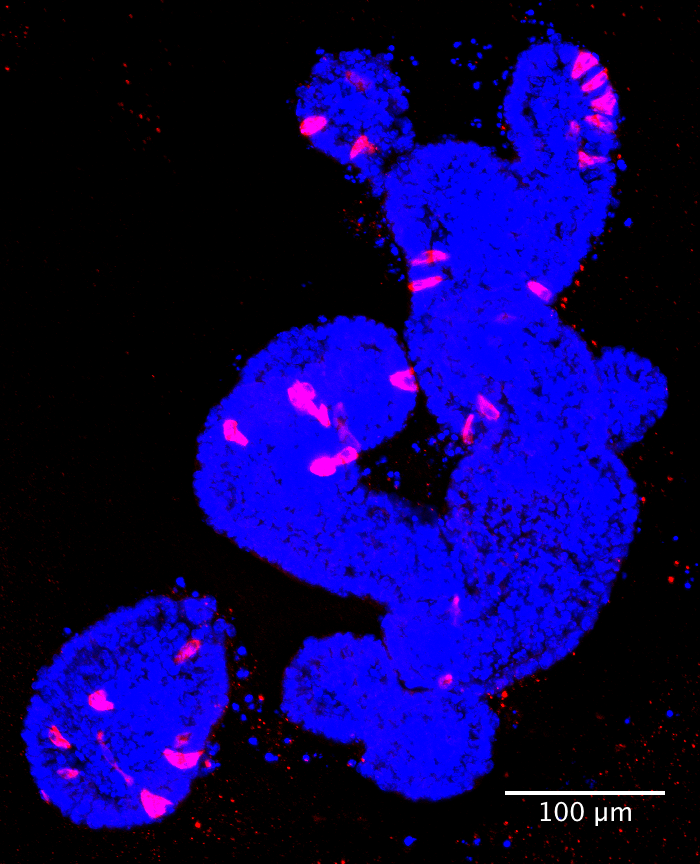

Supplement: Supplementary file 12 — Source data Fig. 9 [file 44318_2024_184_MOESM12_ESM.zip › Figure 9/9B/Dclk1 IF organoid + Thapsigargin + IL13.tif]

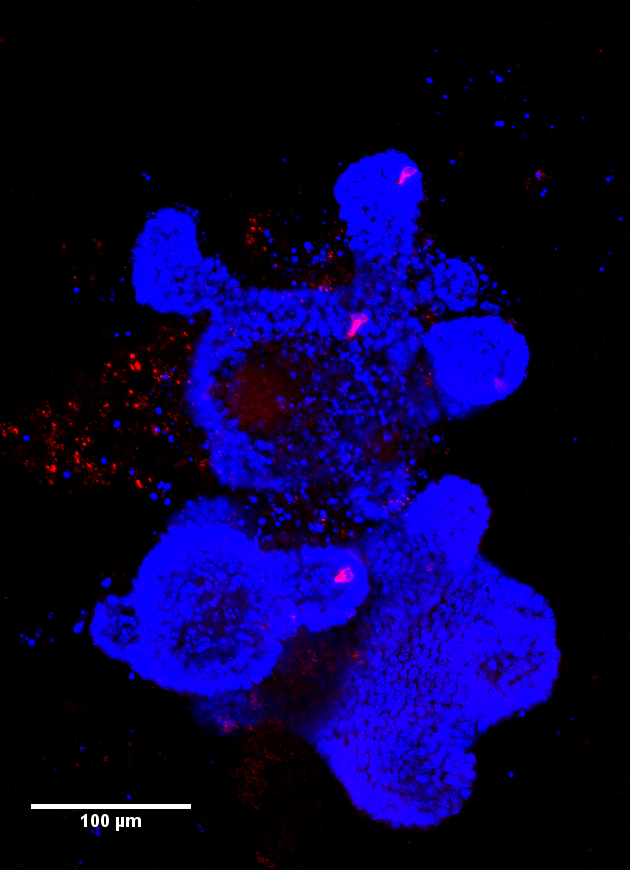

Supplement: Supplementary file 12 — Source data Fig. 9 [file 44318_2024_184_MOESM12_ESM.zip › Figure 9/9B/Dclk1 IF organoid + Thapsigargin.tif]

## Slide 1
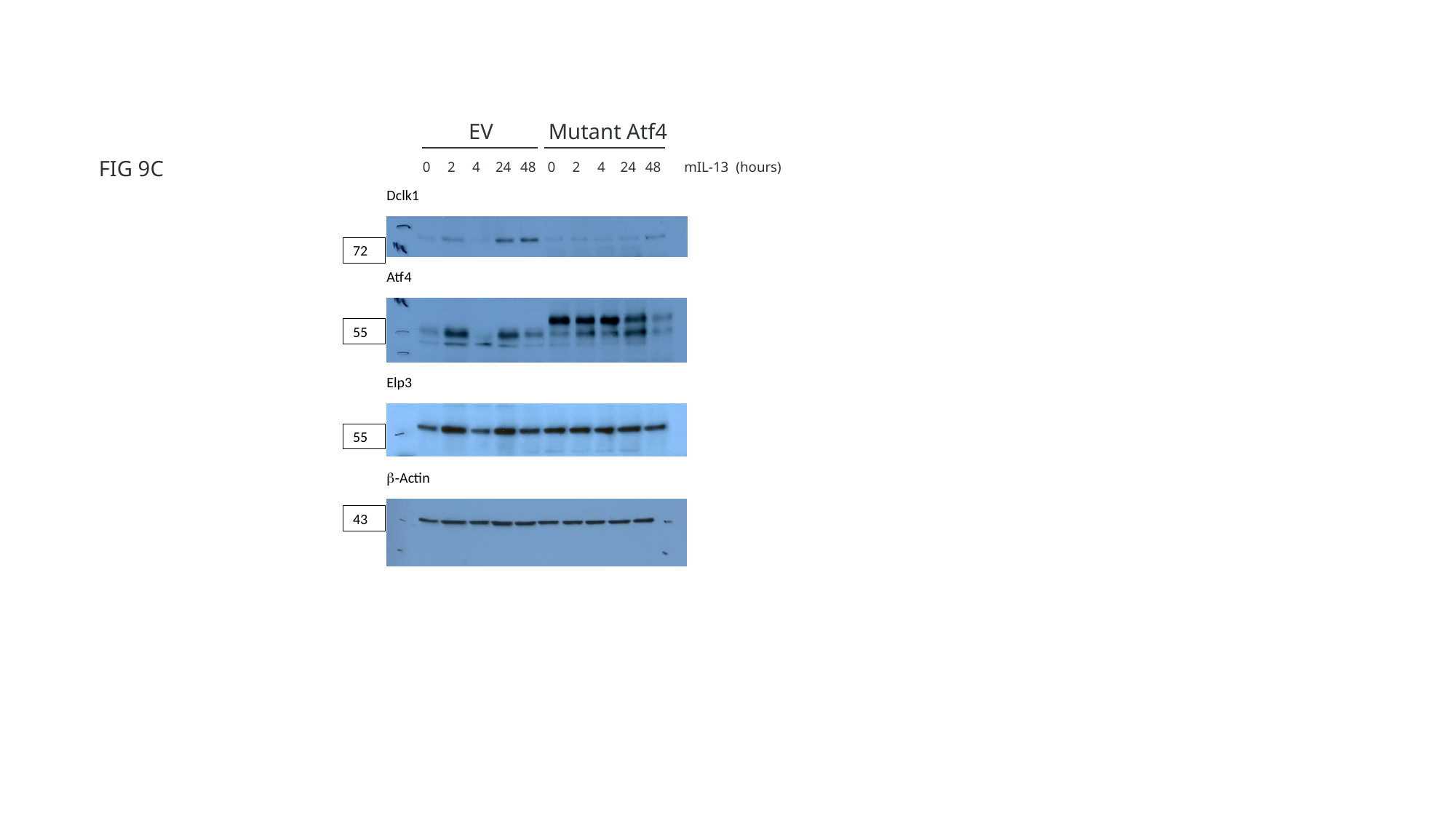

EV
Mutant Atf4
FIG 9C
0
2
4
24
48
0
2
4
24
48
mIL-13 (hours)

Supplement: Supplementary file 12 — Source data Fig. 9 [file 44318_2024_184_MOESM12_ESM.zip › Figure 9/9C/WB uncropped gels.pptx]

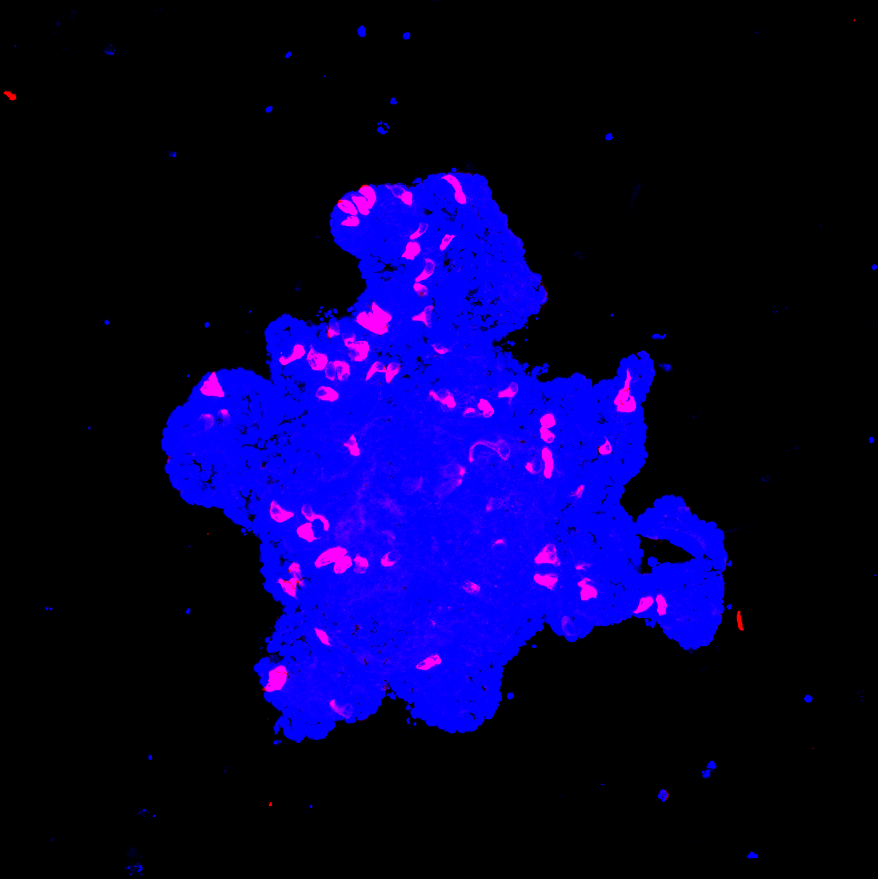

Supplement: Supplementary file 12 — Source data Fig. 9 [file 44318_2024_184_MOESM12_ESM.zip › Figure 9/9D/Dclk1 IF EV organoid + IL13.tif]

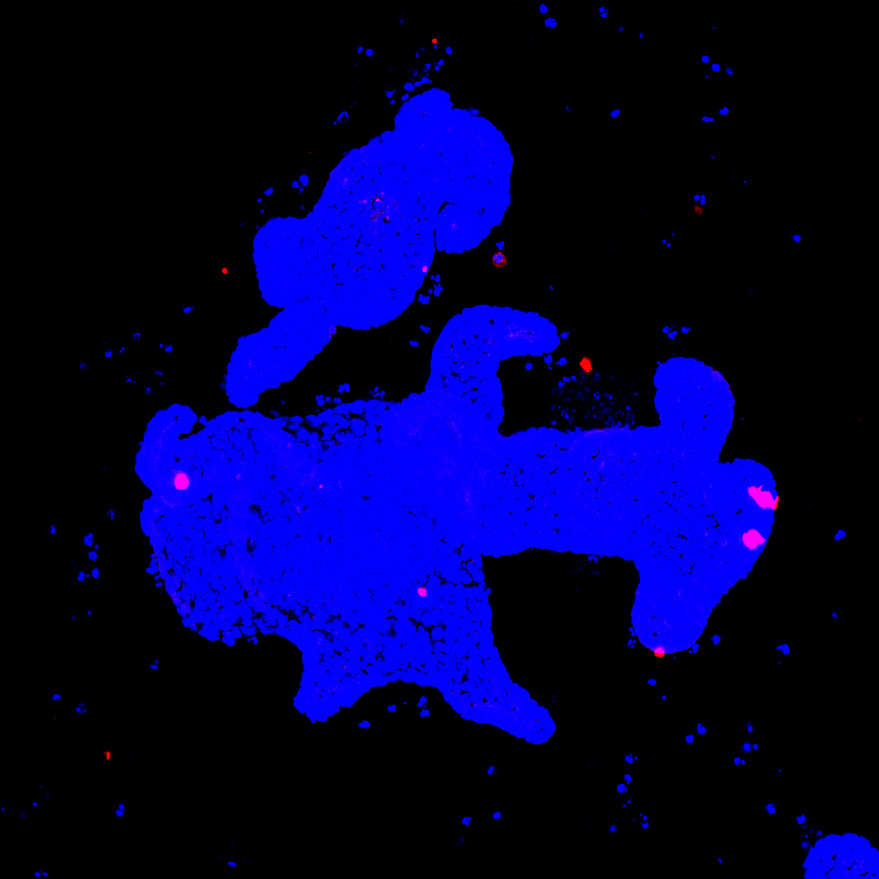

Supplement: Supplementary file 12 — Source data Fig. 9 [file 44318_2024_184_MOESM12_ESM.zip › Figure 9/9D/Dclk1 IF EV organoid + PBS.tif]

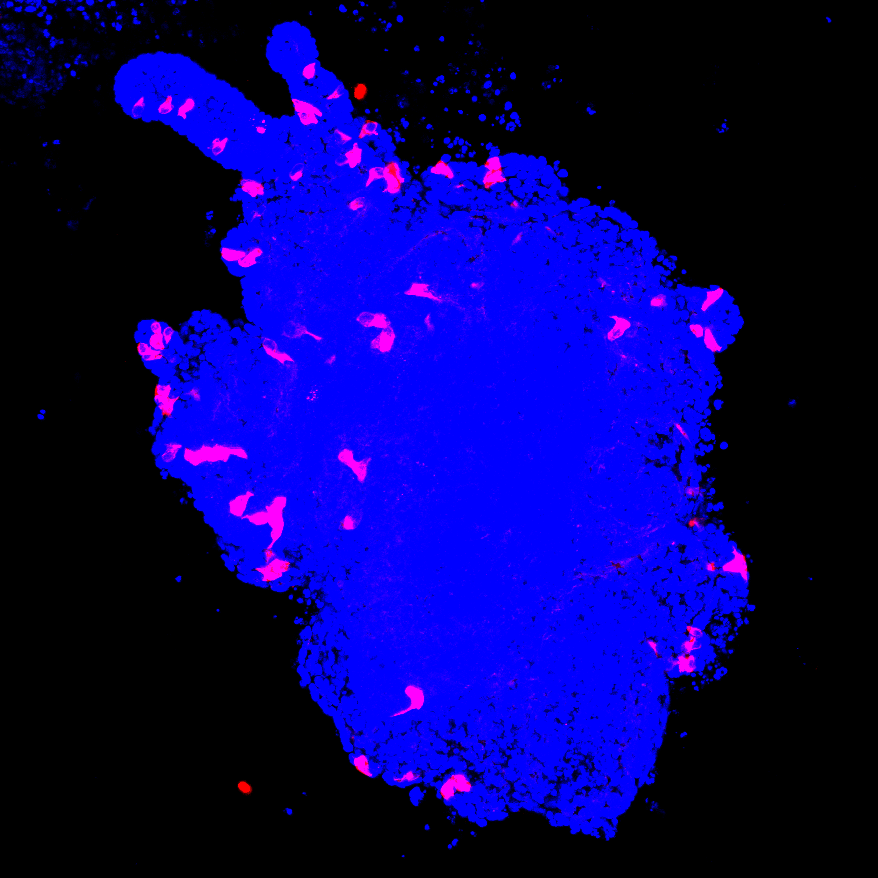

Supplement: Supplementary file 12 — Source data Fig. 9 [file 44318_2024_184_MOESM12_ESM.zip › Figure 9/9D/Dclk1 IF Mutant Atf4 OE organoid + IL13.tif]

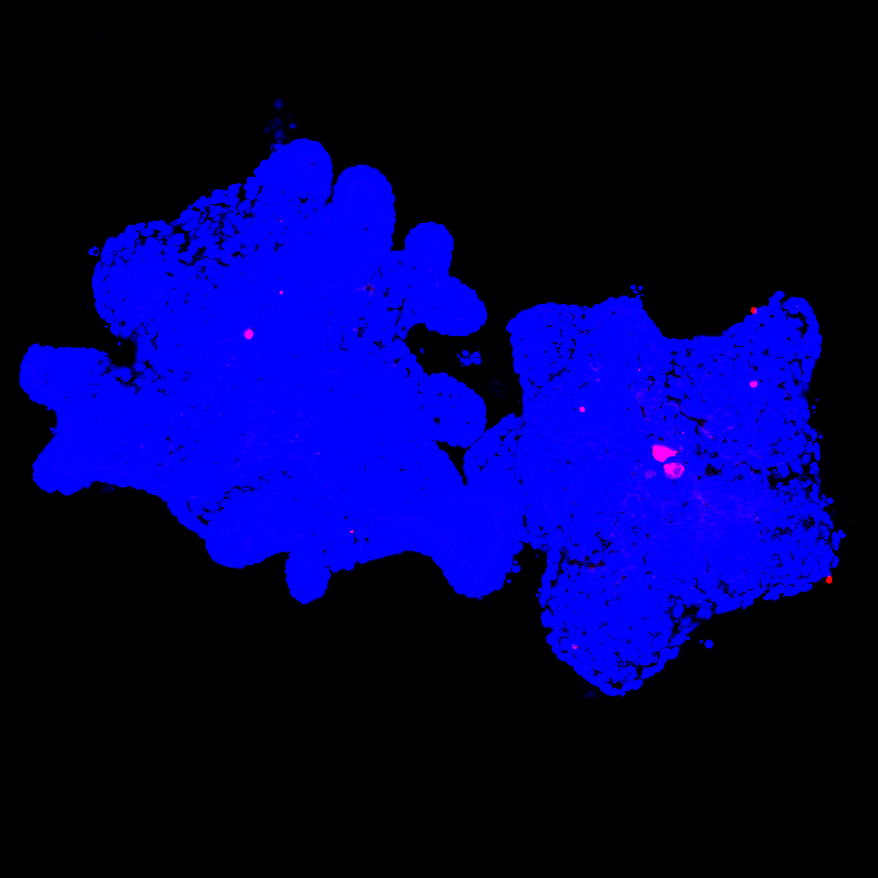

Supplement: Supplementary file 12 — Source data Fig. 9 [file 44318_2024_184_MOESM12_ESM.zip › Figure 9/9D/Dclk1 IF Mutant Atf4 OE organoid + PBS.tif]

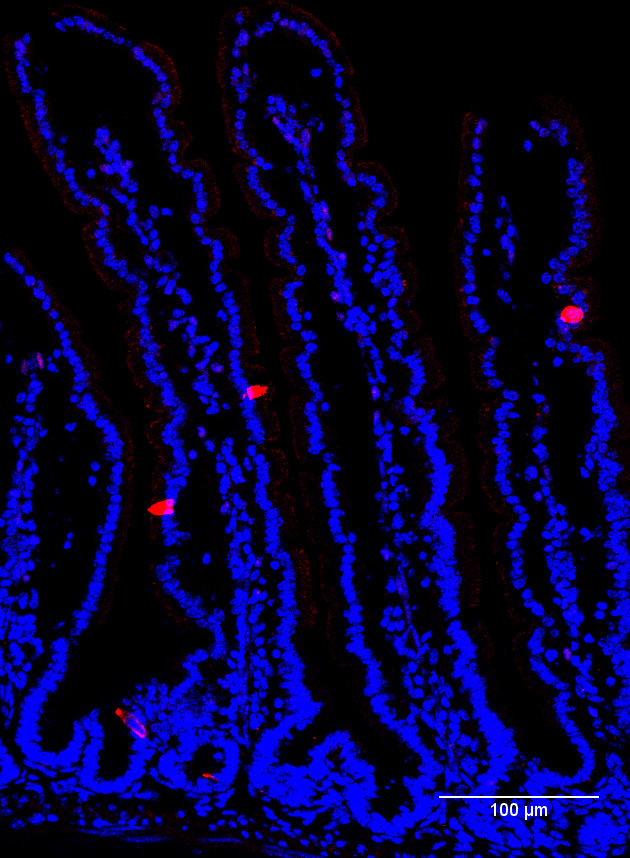

Supplement: Supplementary file 12 — Source data Fig. 9 [file 44318_2024_184_MOESM12_ESM.zip › Figure 9/9H/IF Dclk1 CTR diet + PBS.tif]

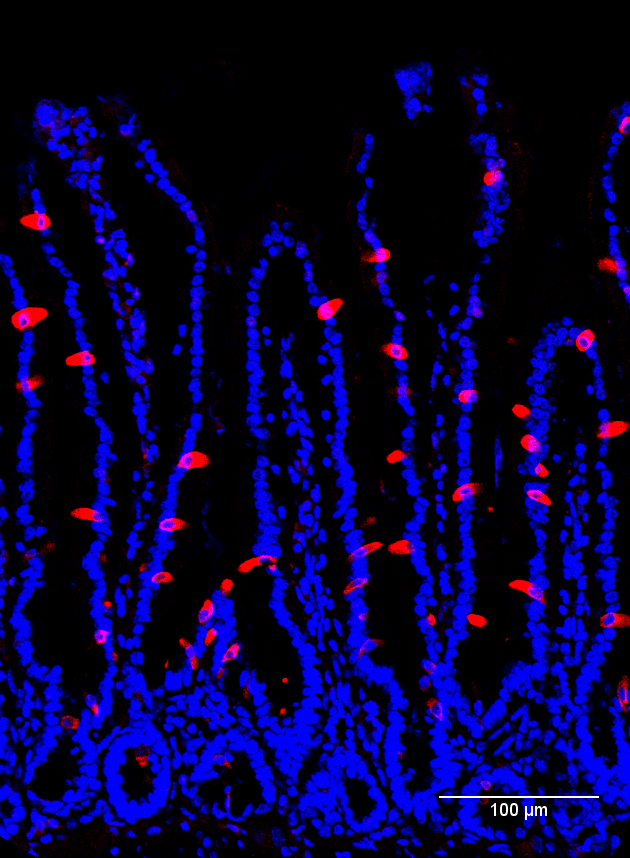

Supplement: Supplementary file 12 — Source data Fig. 9 [file 44318_2024_184_MOESM12_ESM.zip › Figure 9/9H/IF Dclk1 CTR diet + rIL13 D4.tif]

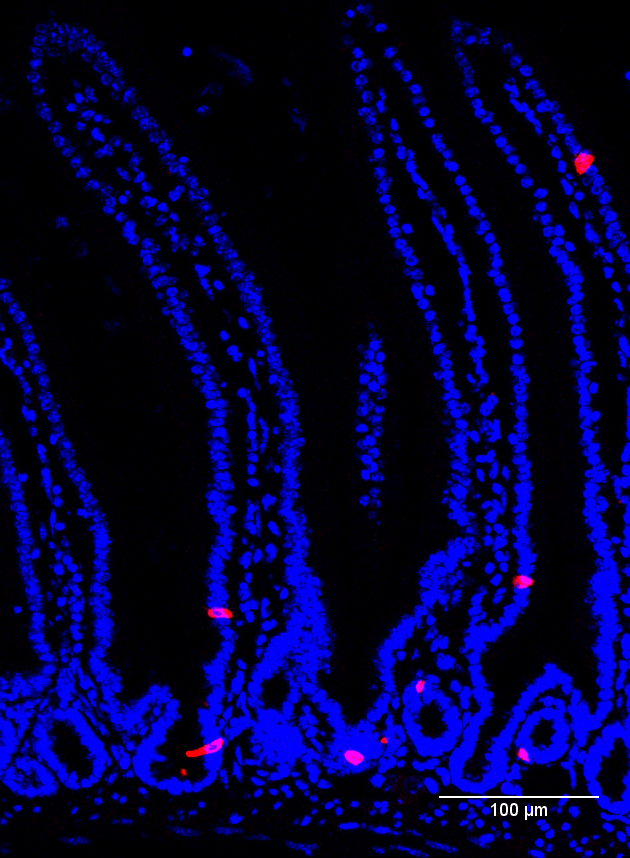

Supplement: Supplementary file 12 — Source data Fig. 9 [file 44318_2024_184_MOESM12_ESM.zip › Figure 9/9H/IF Dclk1 RKV deprived diet + PBS.tif]

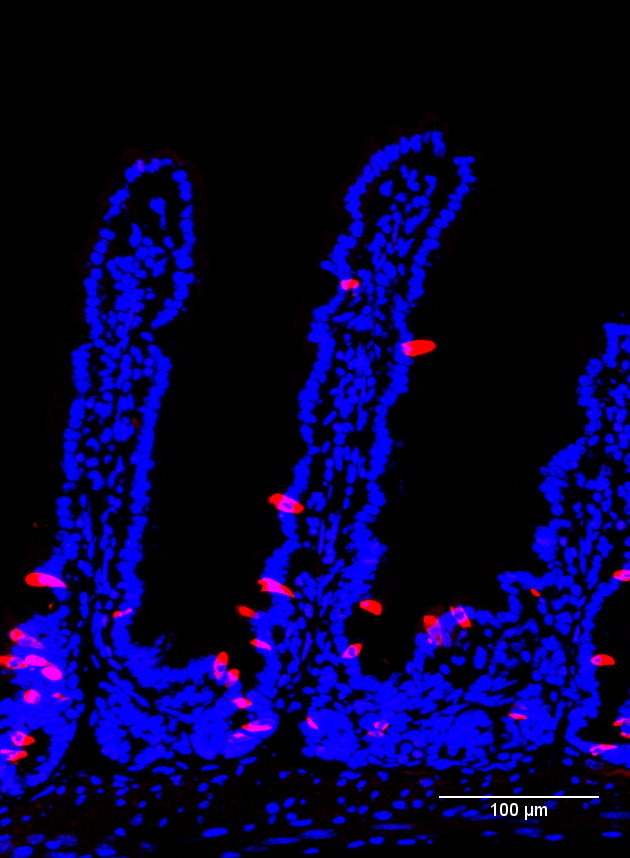

Supplement: Supplementary file 12 — Source data Fig. 9 [file 44318_2024_184_MOESM12_ESM.zip › Figure 9/9H/IF Dclk1 RKV deprived diet + rIL13 D4.tif]

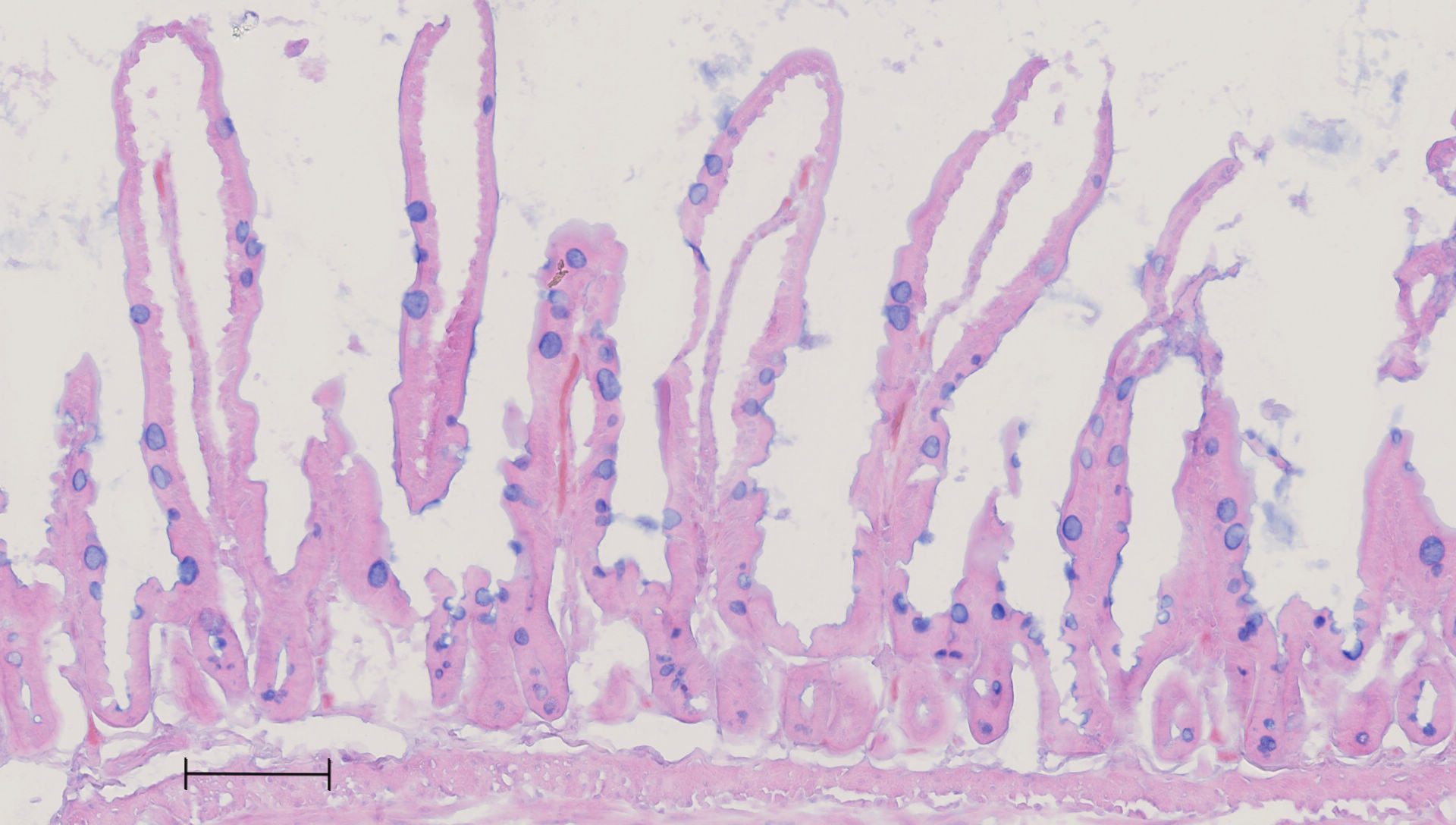

Supplement: Supplementary file 12 — Source data Fig. 9 [file 44318_2024_184_MOESM12_ESM.zip › Figure 9/9I/IHC Alcian Blue Control Diet + IL13.jpg]

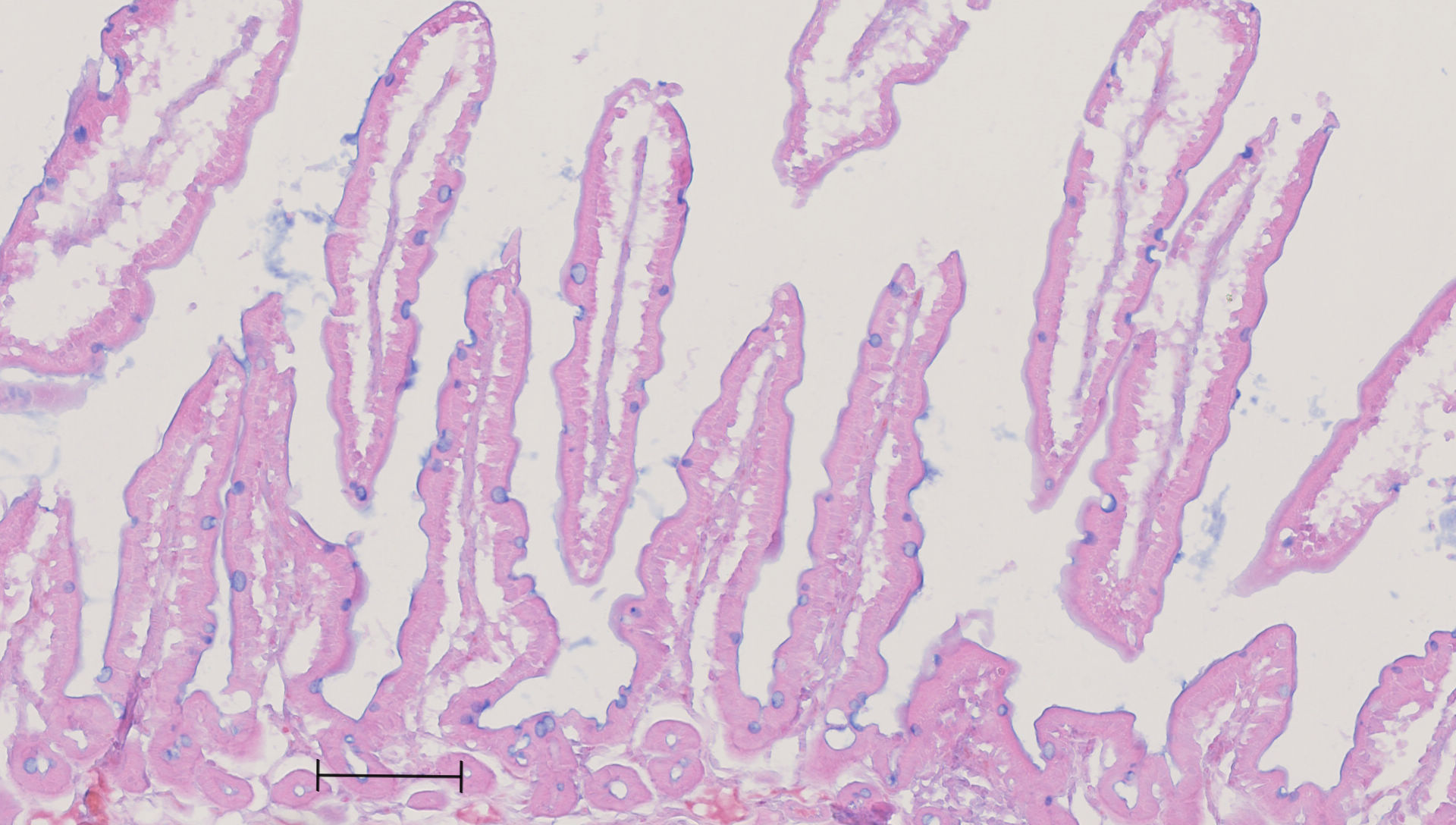

Supplement: Supplementary file 12 — Source data Fig. 9 [file 44318_2024_184_MOESM12_ESM.zip › Figure 9/9I/IHC Alcian Blue Control Diet + PBS.jpg]

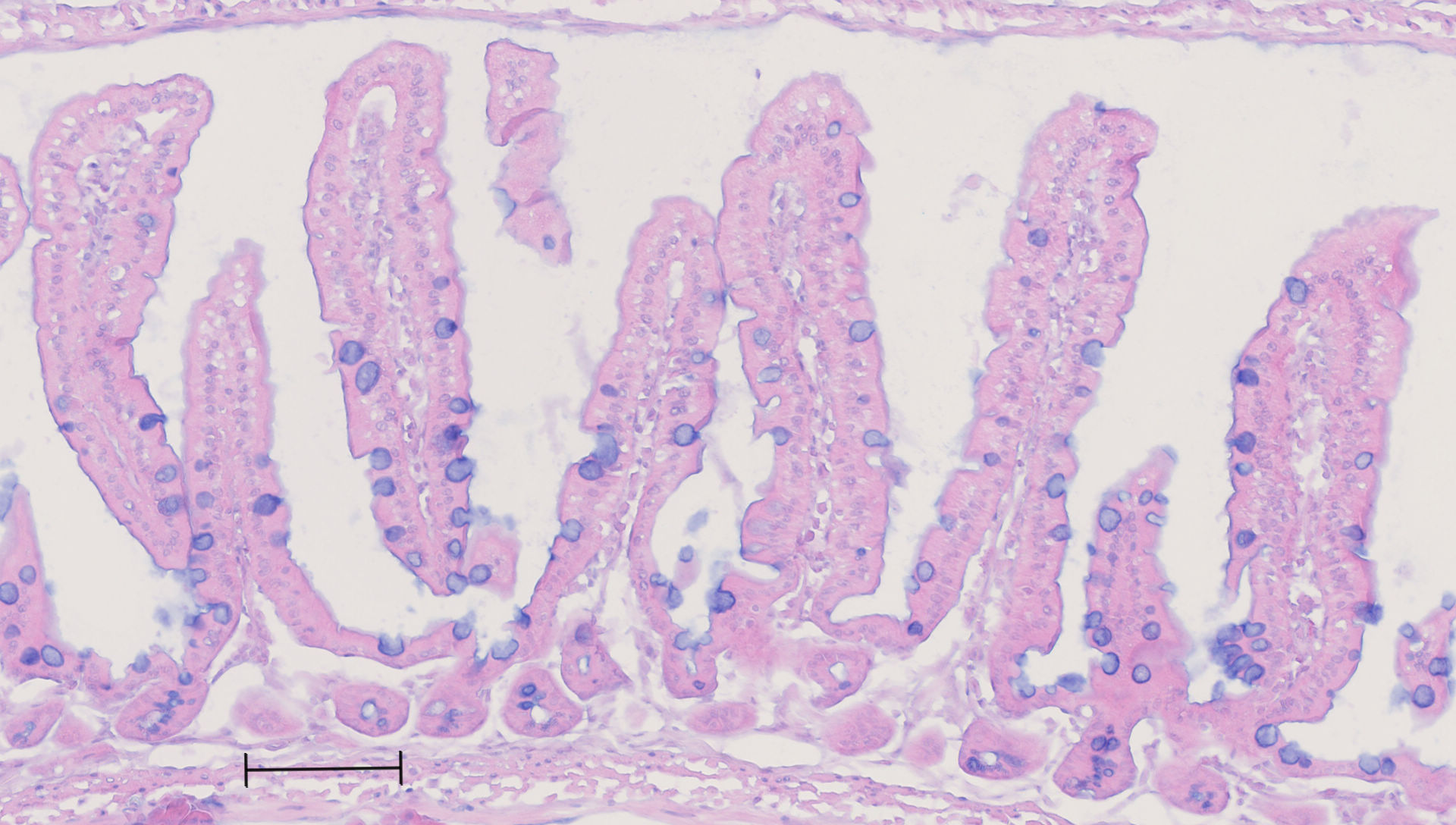

Supplement: Supplementary file 12 — Source data Fig. 9 [file 44318_2024_184_MOESM12_ESM.zip › Figure 9/9I/IHC Alcian Blue RKV deprived Diet + IL13.jpg]

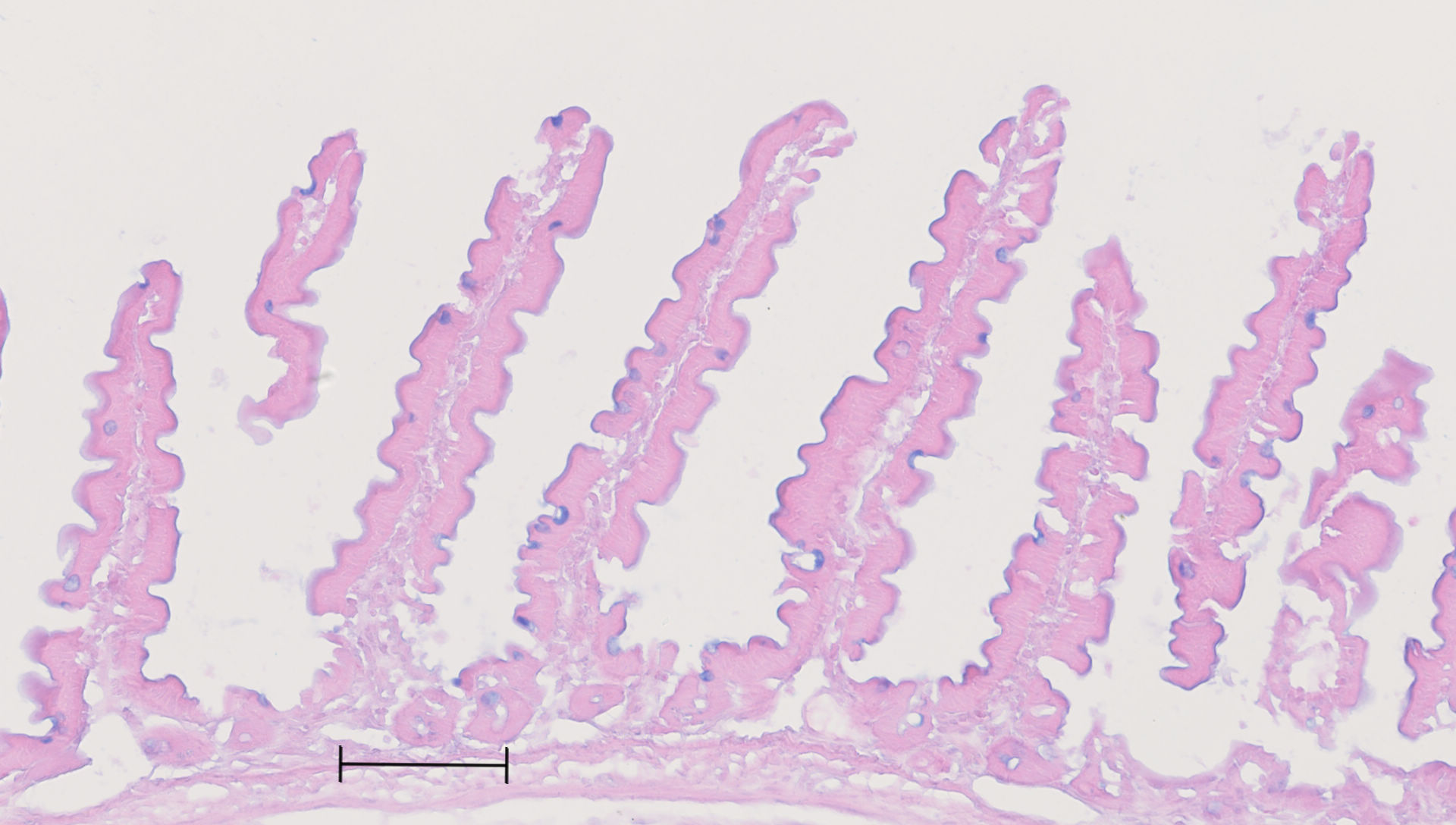

Supplement: Supplementary file 12 — Source data Fig. 9 [file 44318_2024_184_MOESM12_ESM.zip › Figure 9/9I/IHC Alcian Blue RKV deprived Diet + PBS.jpg]

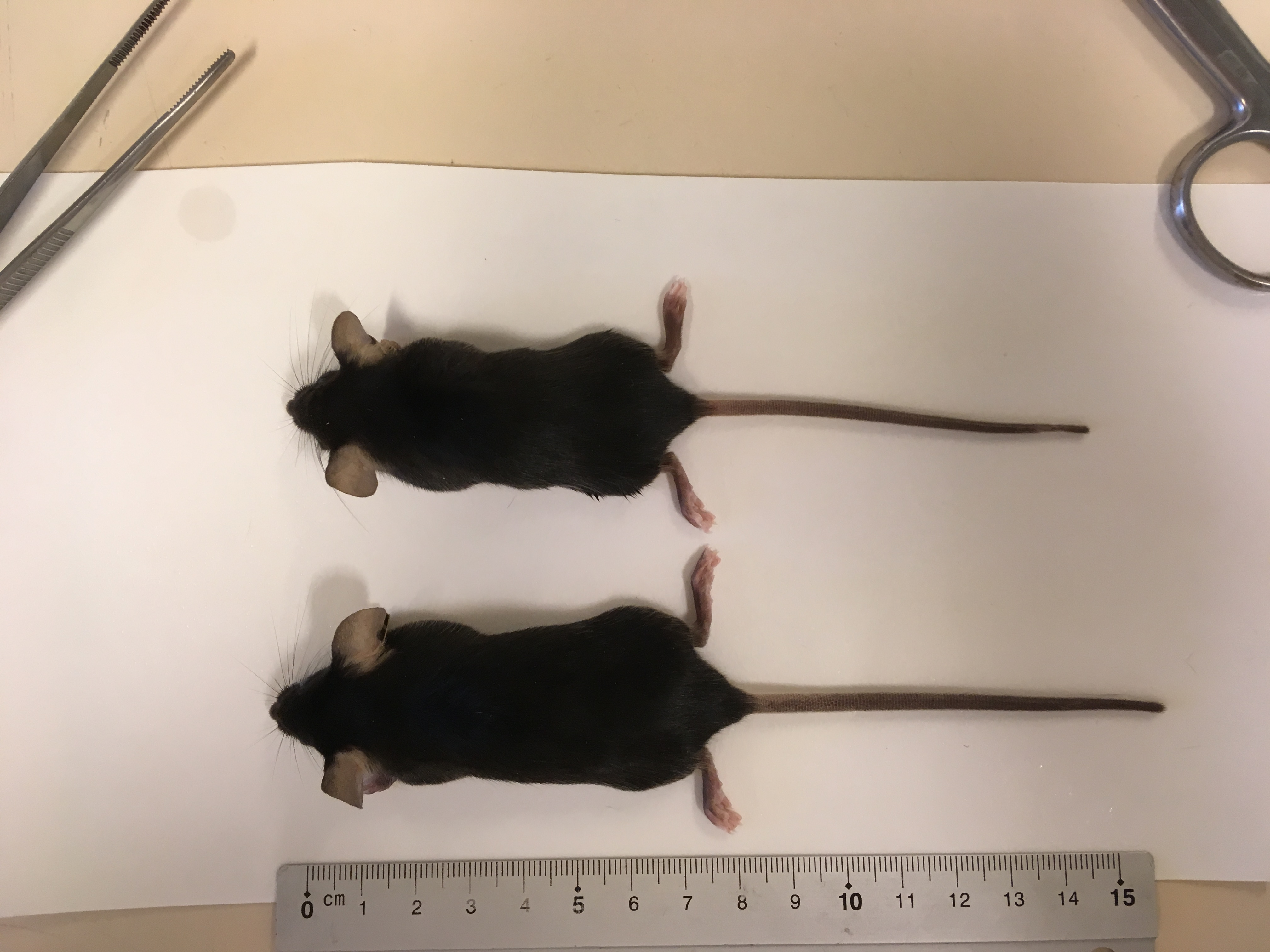

Supplement: Supplementary file 13 — Source data Fig. 10 [file 44318_2024_184_MOESM13_ESM.zip › Figure 10/10A/IMG_2709.JPG]

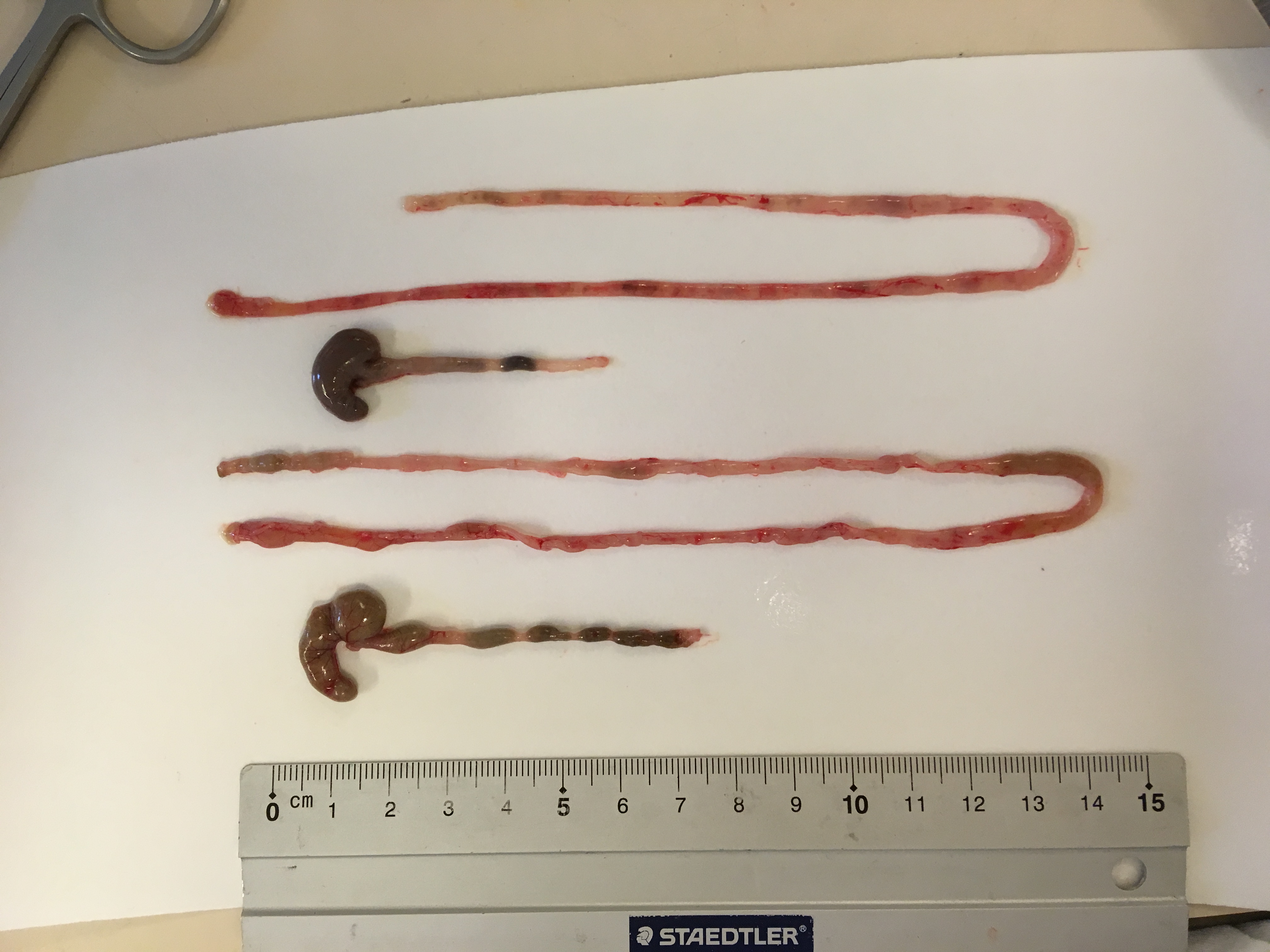

Supplement: Supplementary file 13 — Source data Fig. 10 [file 44318_2024_184_MOESM13_ESM.zip › Figure 10/10B/IMG_2729.JPG]

## Slide 1
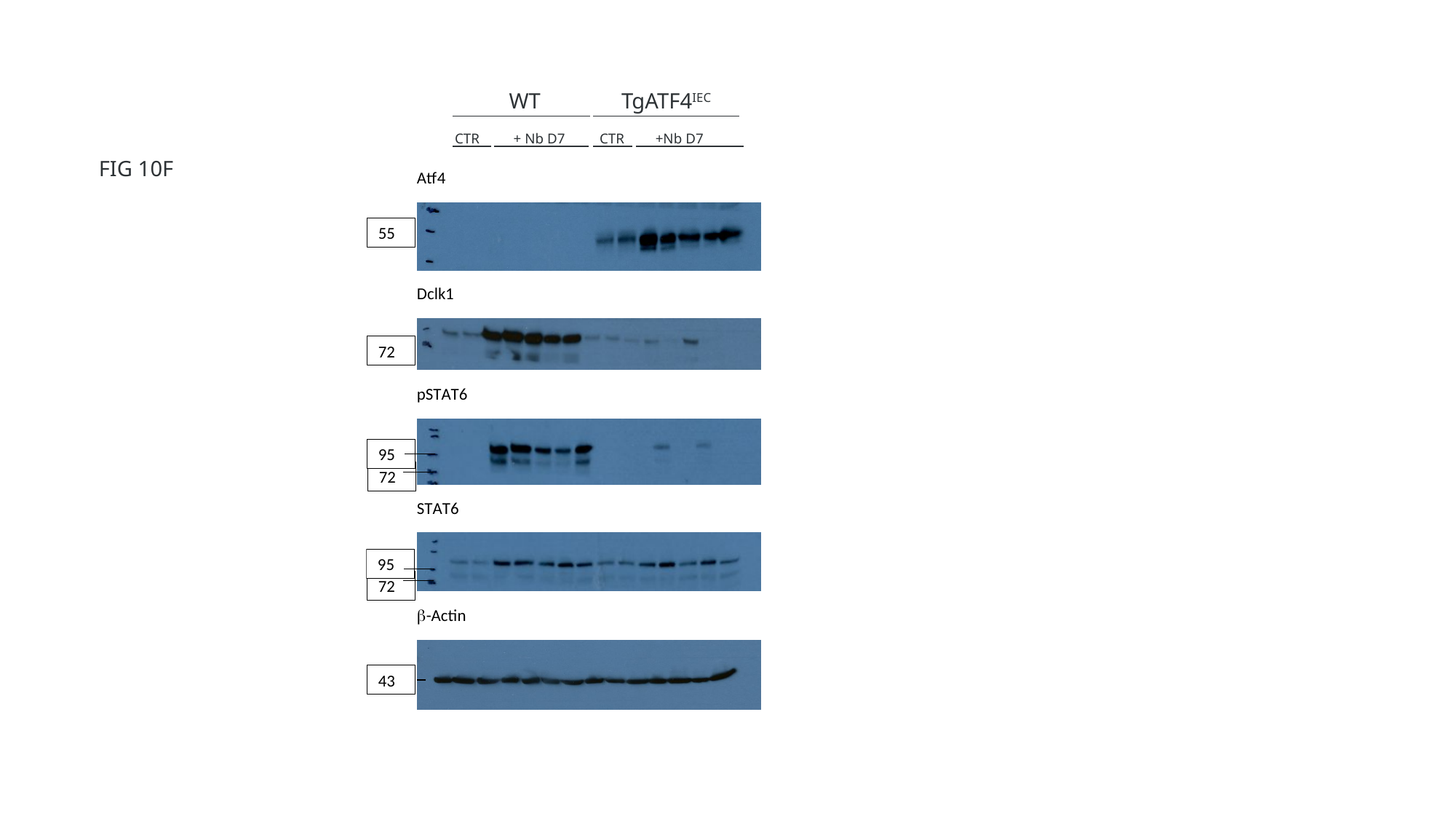

WT
TgATF4IEC
CTR
+ Nb D7
CTR
+Nb D7
FIG 10F

Supplement: Supplementary file 13 — Source data Fig. 10 [file 44318_2024_184_MOESM13_ESM.zip › Figure 10/10F/WB uncropped gels.pptx]

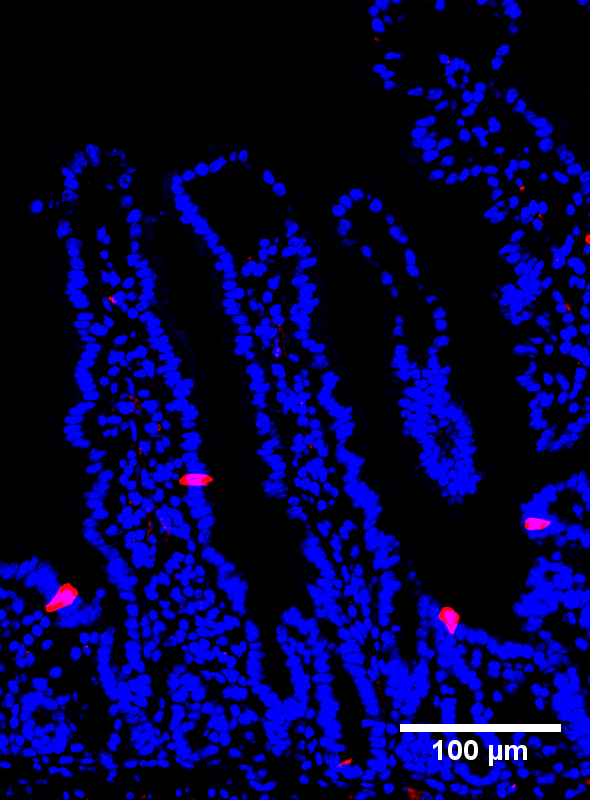

Supplement: Supplementary file 13 — Source data Fig. 10 [file 44318_2024_184_MOESM13_ESM.zip › Figure 10/10G/Dclk1 IF TgAtf4 + Nb D7.tif]
