# Supplementary figures and images for: Loss of Elp3 blocks intestinal tuft cell differentiation via an mTORC1-Atf4 axis (part 2 of 2)
Source: EMBO J. 2024 Jul 31;43(18):6. doi: 10.1038/s44318-024-00184-4 (PMC11405396; doi:10.1038/s44318-024-00184-4)

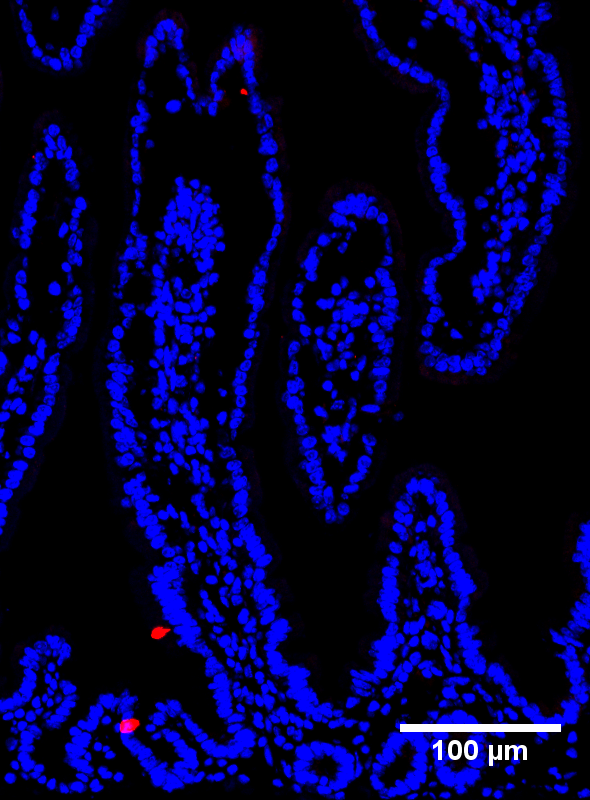

Supplement: Supplementary file 13 — Source data Fig. 10 [file 44318_2024_184_MOESM13_ESM.zip › Figure 10/10G/Dclk1 IF TgAtf4 NI.tif]

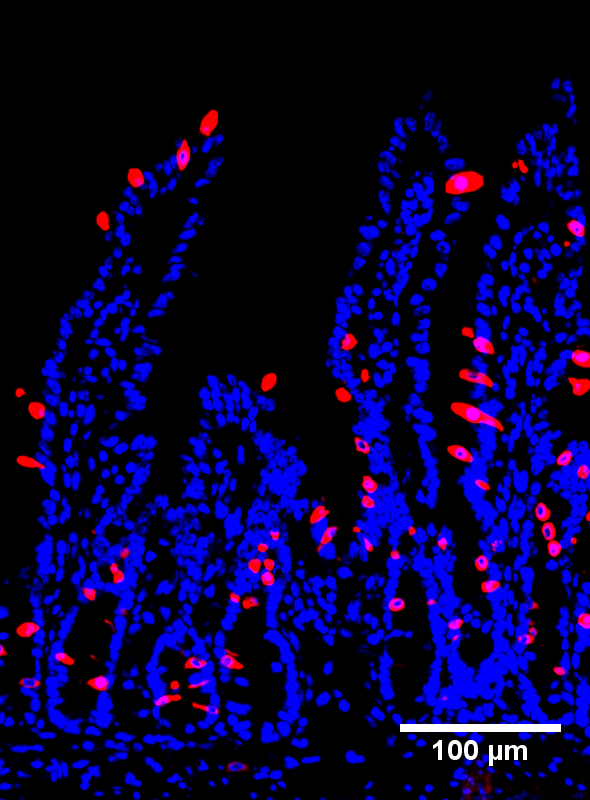

Supplement: Supplementary file 13 — Source data Fig. 10 [file 44318_2024_184_MOESM13_ESM.zip › Figure 10/10G/Dclk1 IF WT + Nb D7.tif]

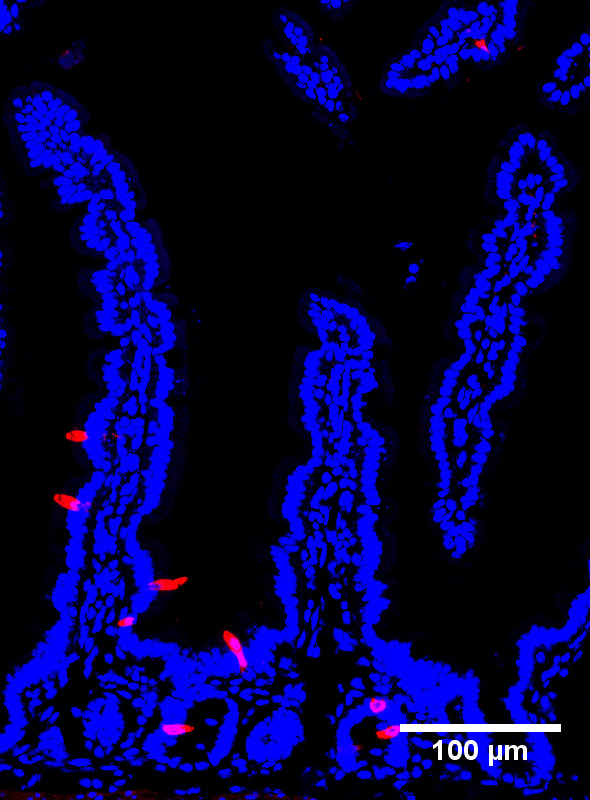

Supplement: Supplementary file 13 — Source data Fig. 10 [file 44318_2024_184_MOESM13_ESM.zip › Figure 10/10G/Dclk1 IF WT NI.tif]

## Slide 1
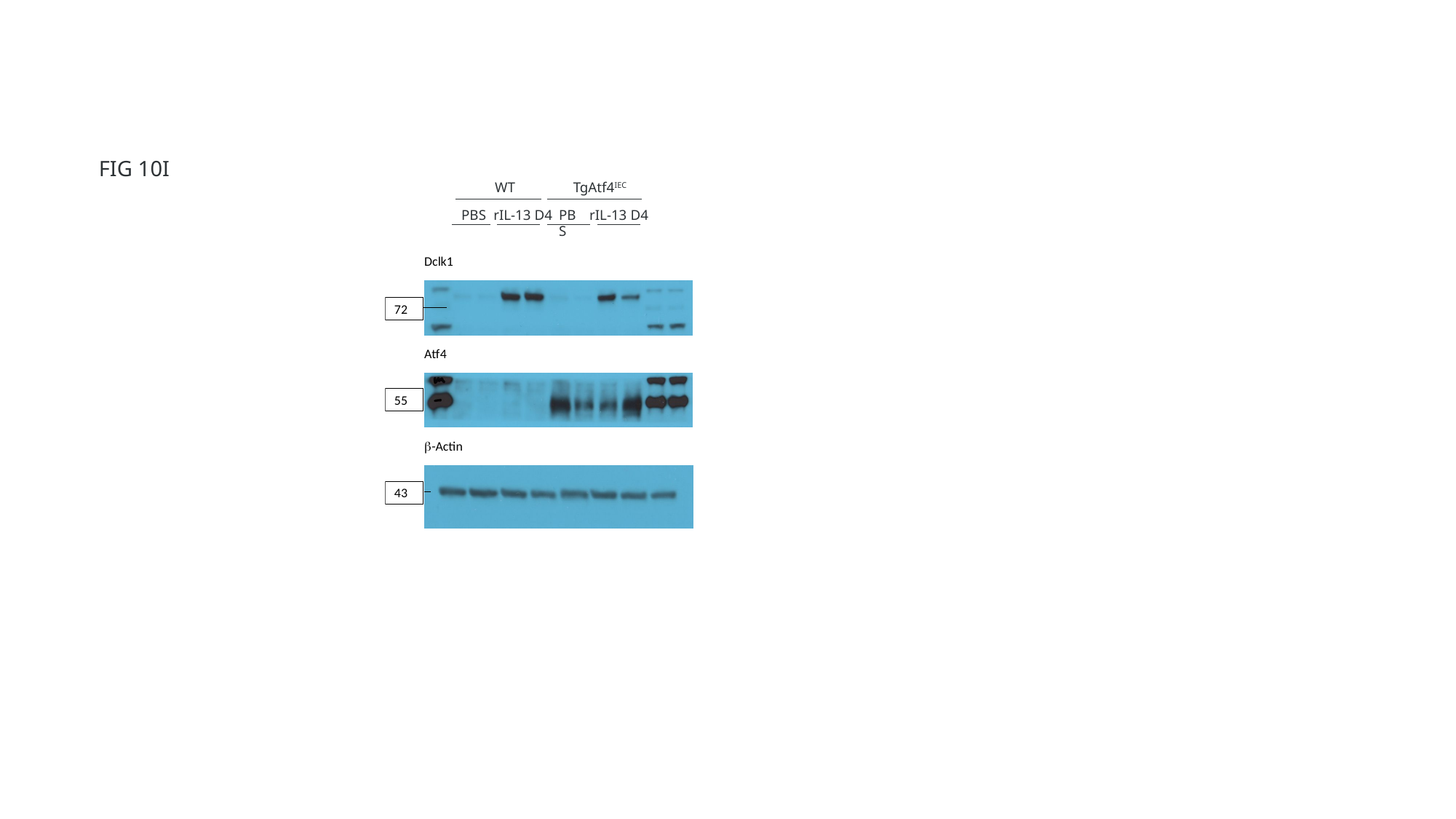

FIG 10I
WT
TgAtf4IEC
PBS
rIL-13 D4
PBS
rIL-13 D4

Supplement: Supplementary file 13 — Source data Fig. 10 [file 44318_2024_184_MOESM13_ESM.zip › Figure 10/10I/WB uncropped gels.pptx]

## Slide 1
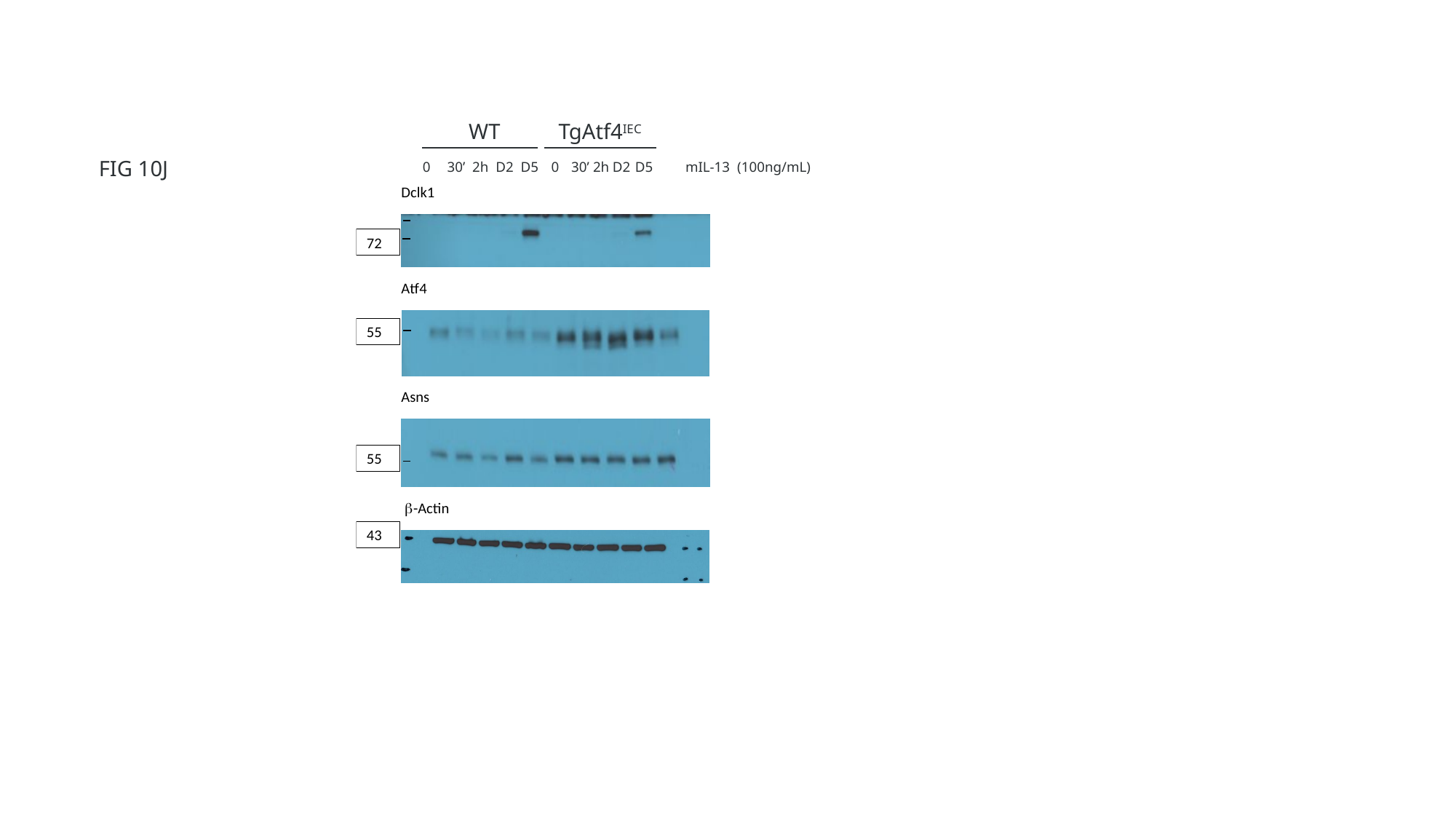

WT
TgAtf4IEC
FIG 10J
0
30’
2h
D2
D5
0
30’
2h
D2
D5
mIL-13 (100ng/mL)

Supplement: Supplementary file 13 — Source data Fig. 10 [file 44318_2024_184_MOESM13_ESM.zip › Figure 10/10J/WB uncropped gels.pptx]

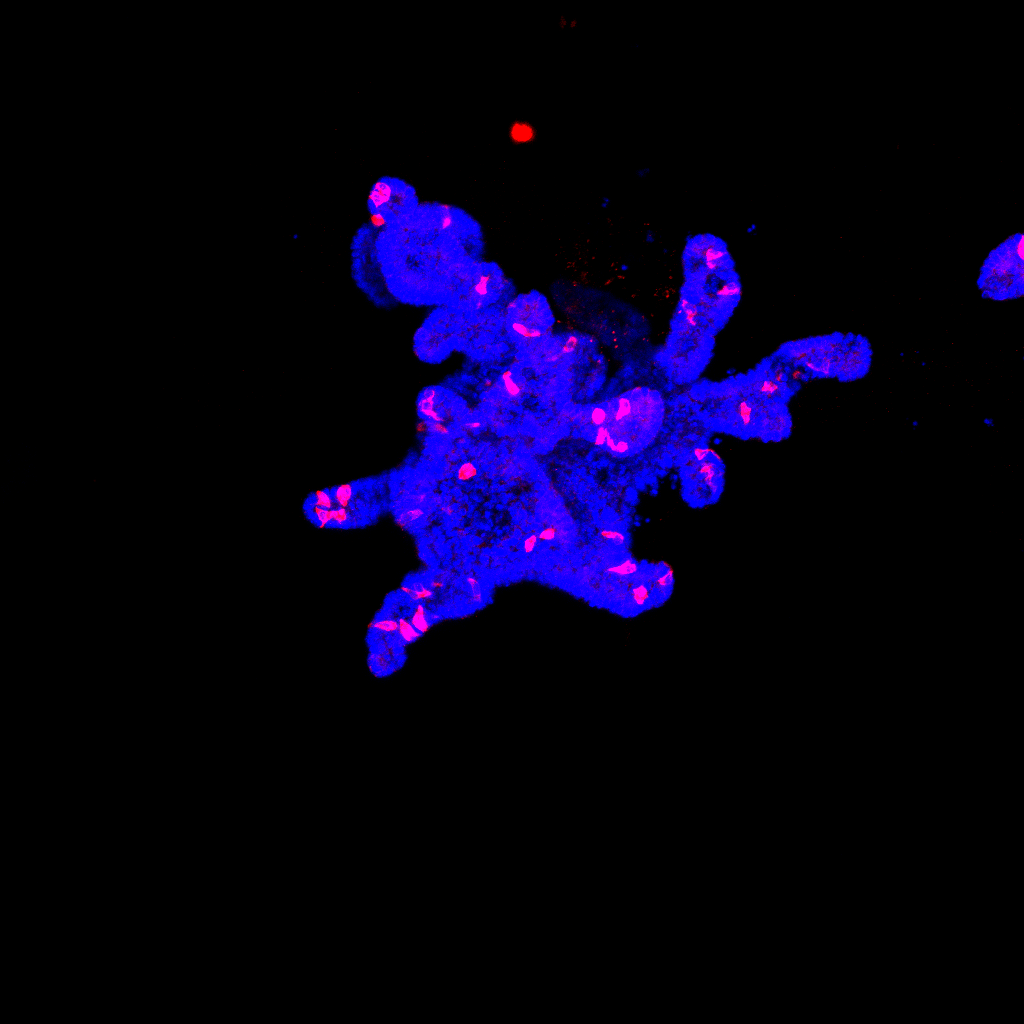

Supplement: Supplementary file 13 — Source data Fig. 10 [file 44318_2024_184_MOESM13_ESM.zip › Figure 10/10K/Dclk1 TgATF4 + IL13.tif]

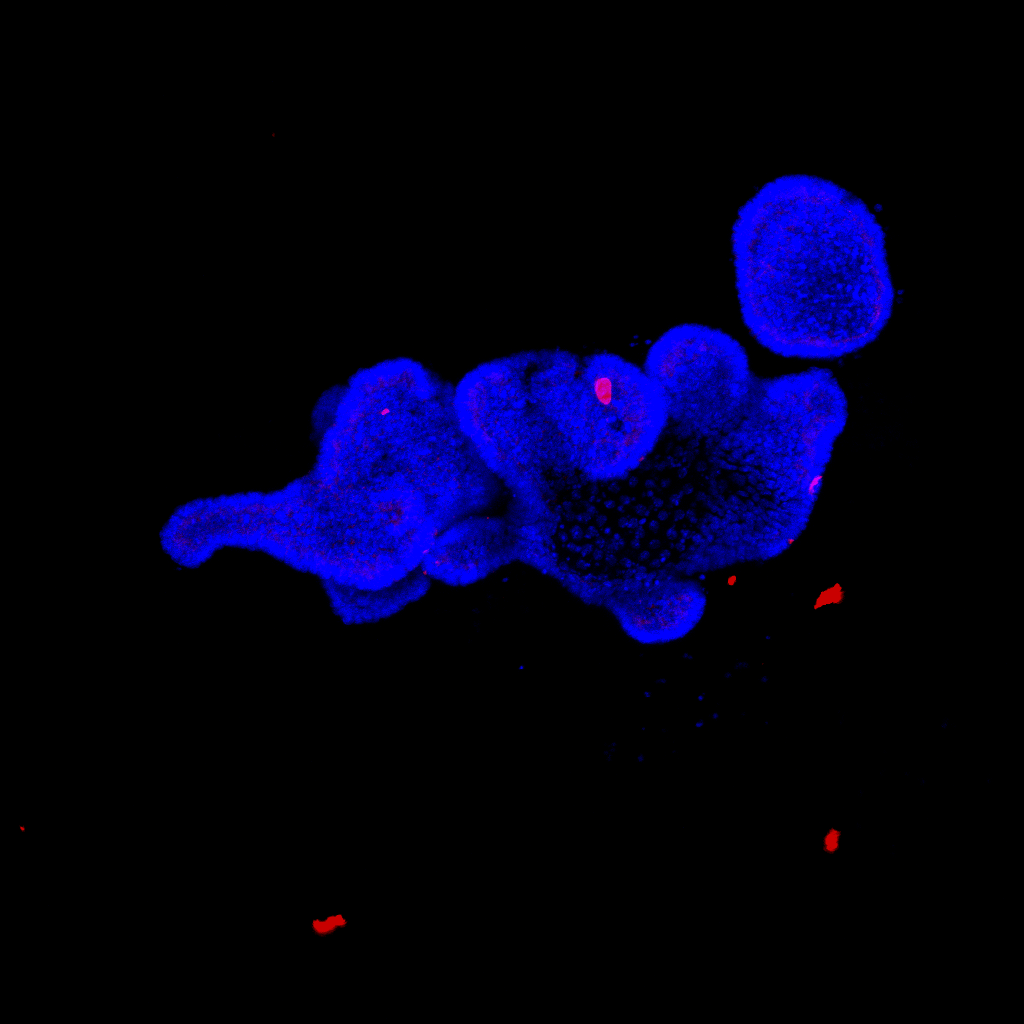

Supplement: Supplementary file 13 — Source data Fig. 10 [file 44318_2024_184_MOESM13_ESM.zip › Figure 10/10K/Dclk1 TgATF4 ctrl.tif]

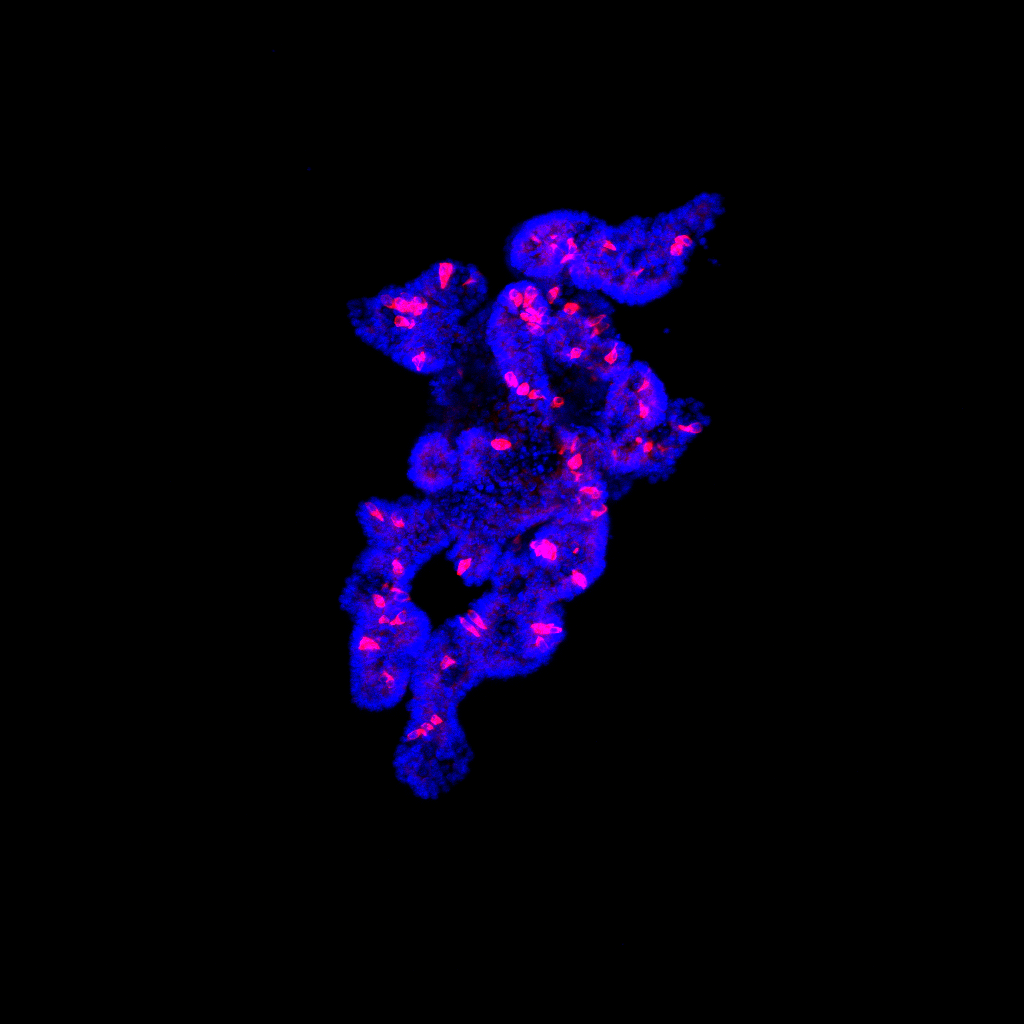

Supplement: Supplementary file 13 — Source data Fig. 10 [file 44318_2024_184_MOESM13_ESM.zip › Figure 10/10K/Dclk1 WT + IL13.tif]

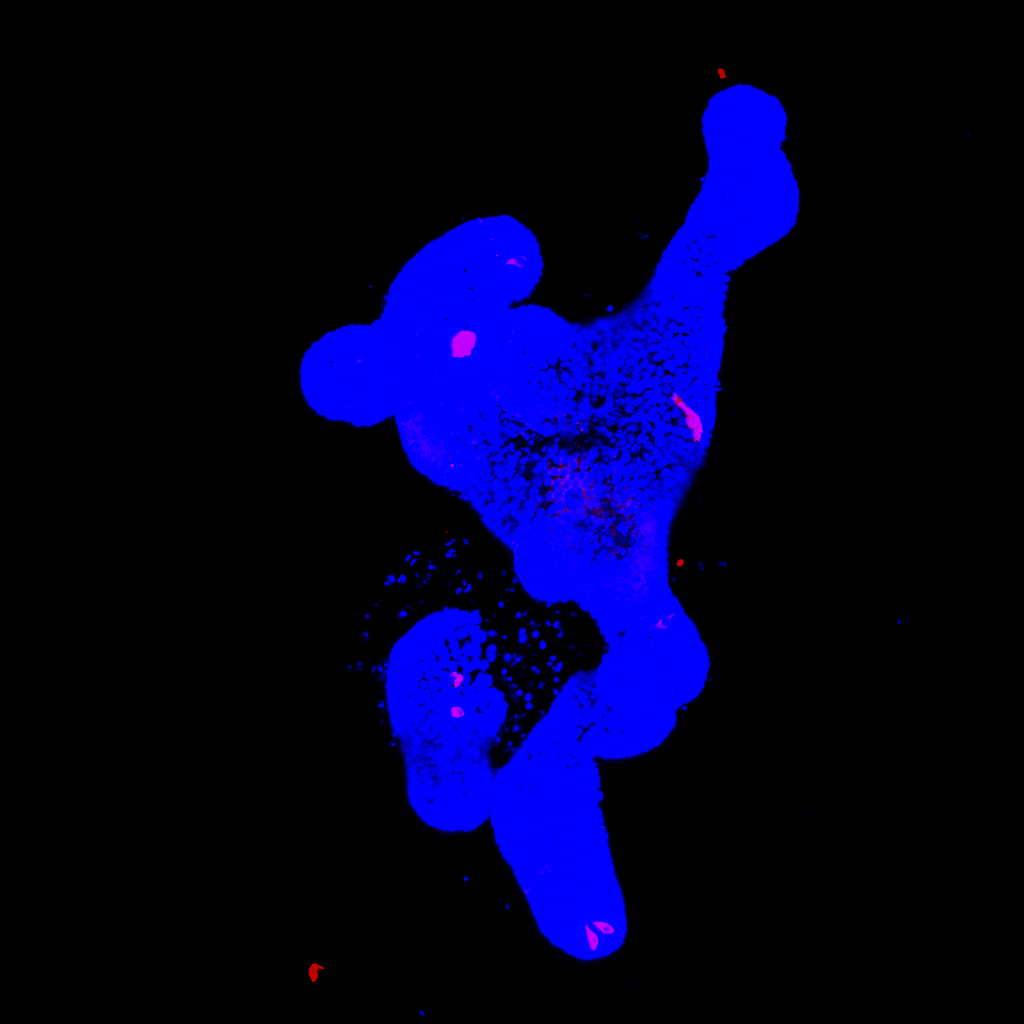

Supplement: Supplementary file 13 — Source data Fig. 10 [file 44318_2024_184_MOESM13_ESM.zip › Figure 10/10K/Dclk1 WT ctrl.tif]

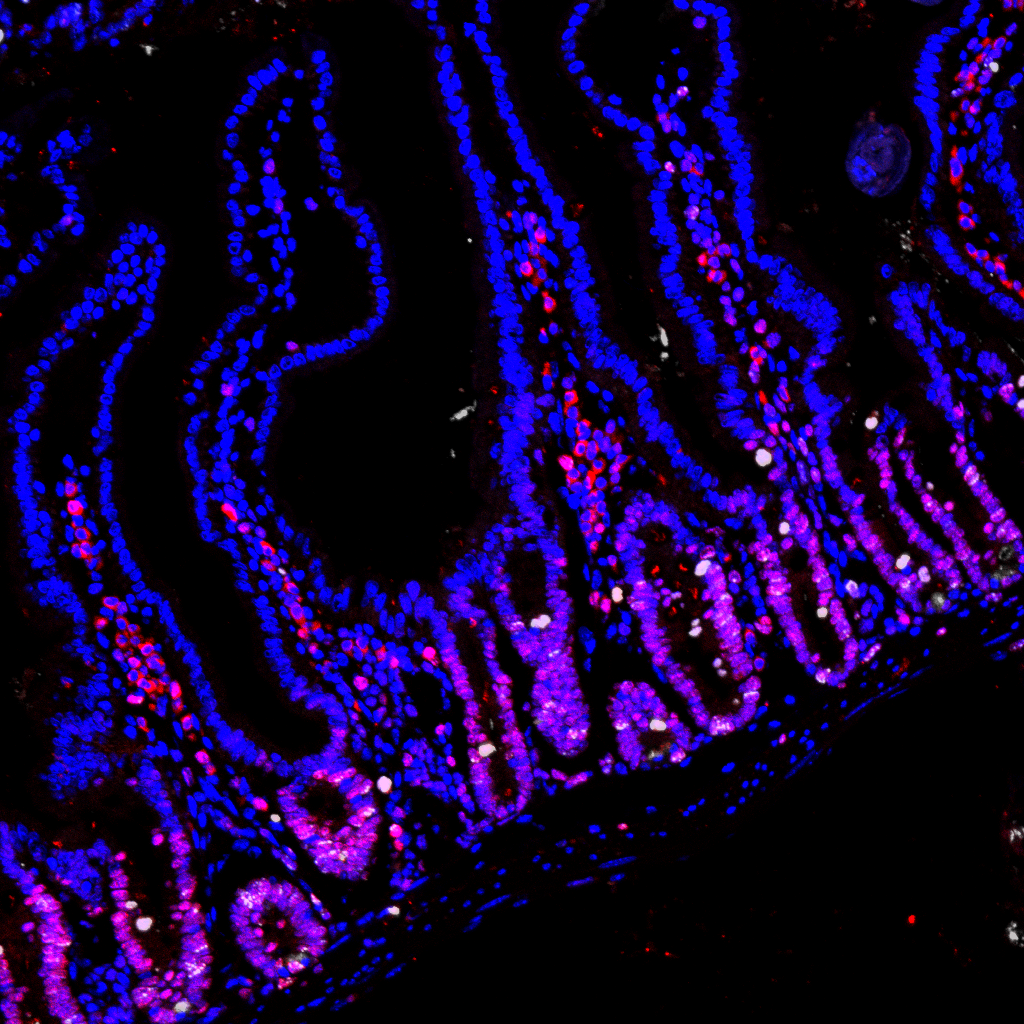

Supplement: Supplementary file 14 — EV Figure Source data [file 44318_2024_184_MOESM14_ESM.zip › EV figures/Figure EV1/EV1 A/IF pHH3 Ki67 KO D7.tif]

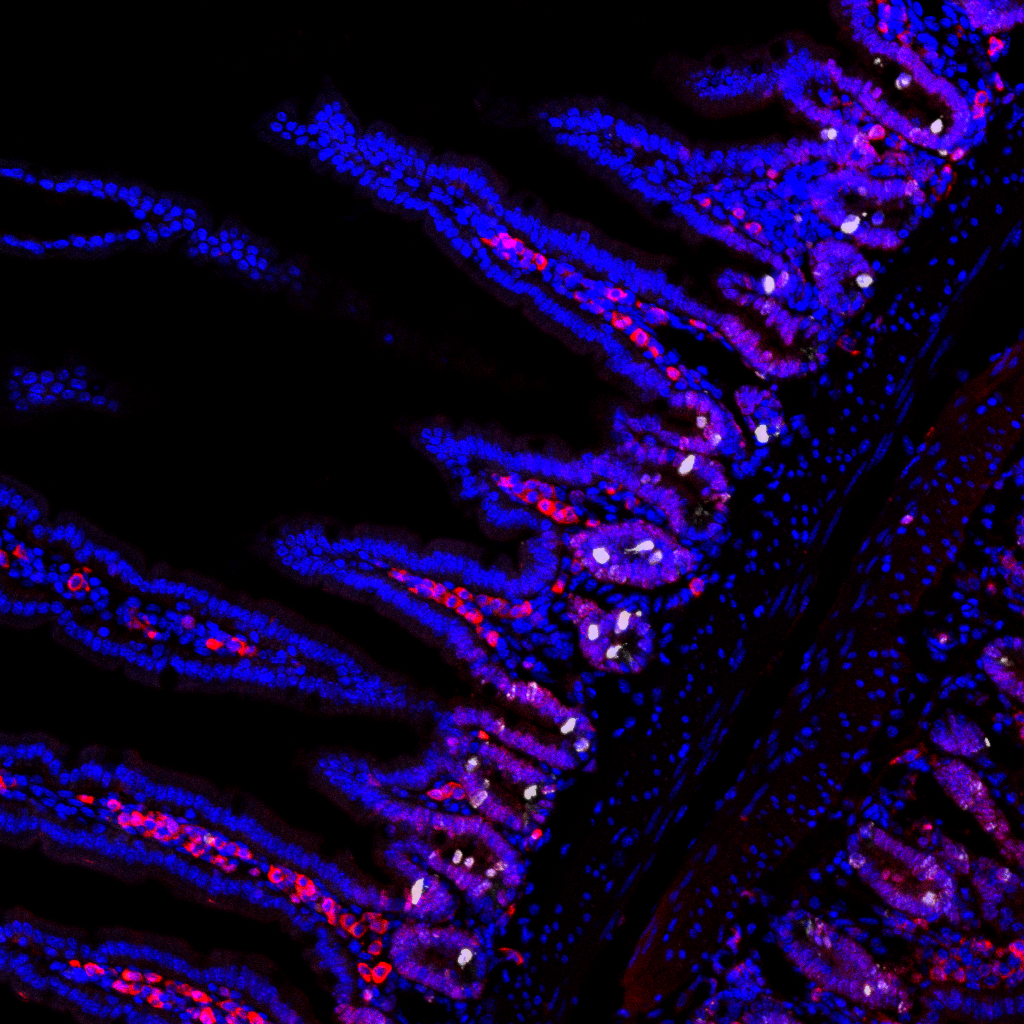

Supplement: Supplementary file 14 — EV Figure Source data [file 44318_2024_184_MOESM14_ESM.zip › EV figures/Figure EV1/EV1 A/IF pHH3 Ki67 KO NI.tif]

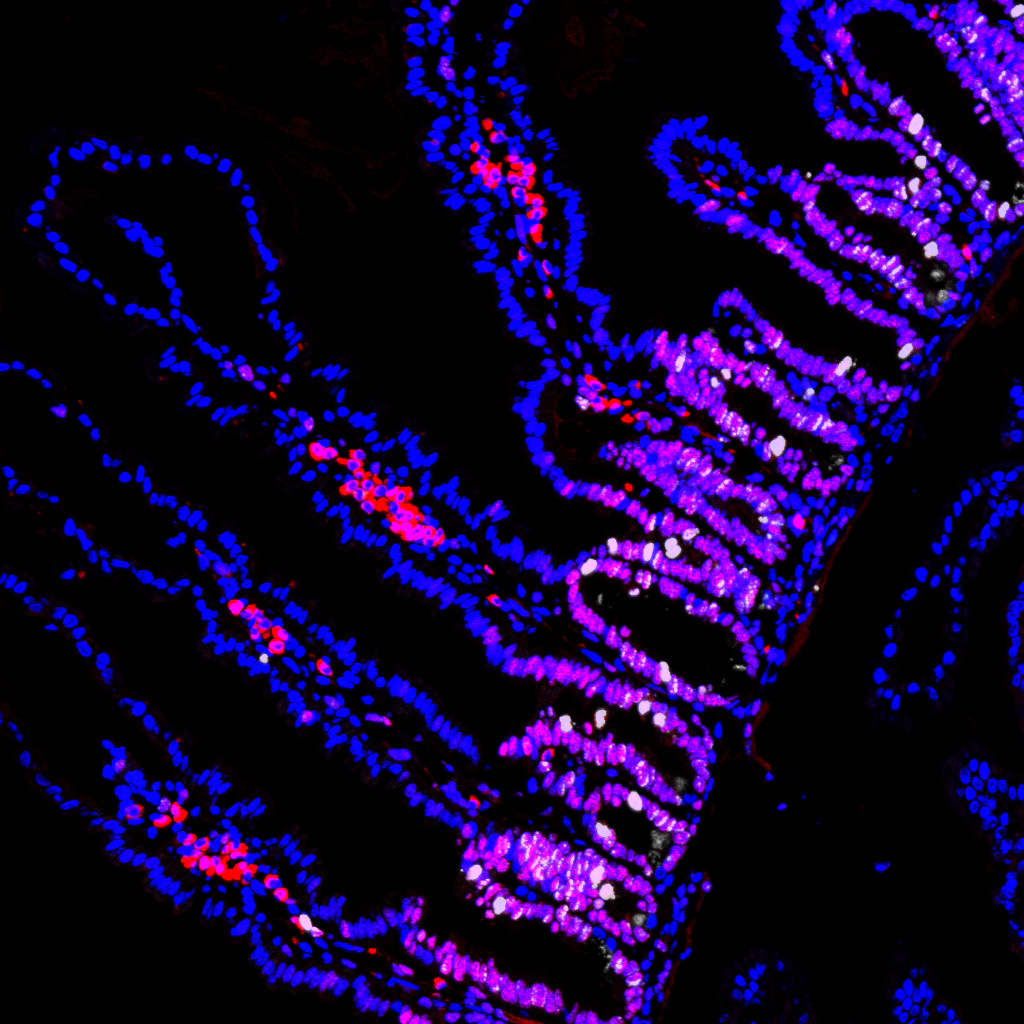

Supplement: Supplementary file 14 — EV Figure Source data [file 44318_2024_184_MOESM14_ESM.zip › EV figures/Figure EV1/EV1 A/IF pHH3 Ki67 WT D7.tif]

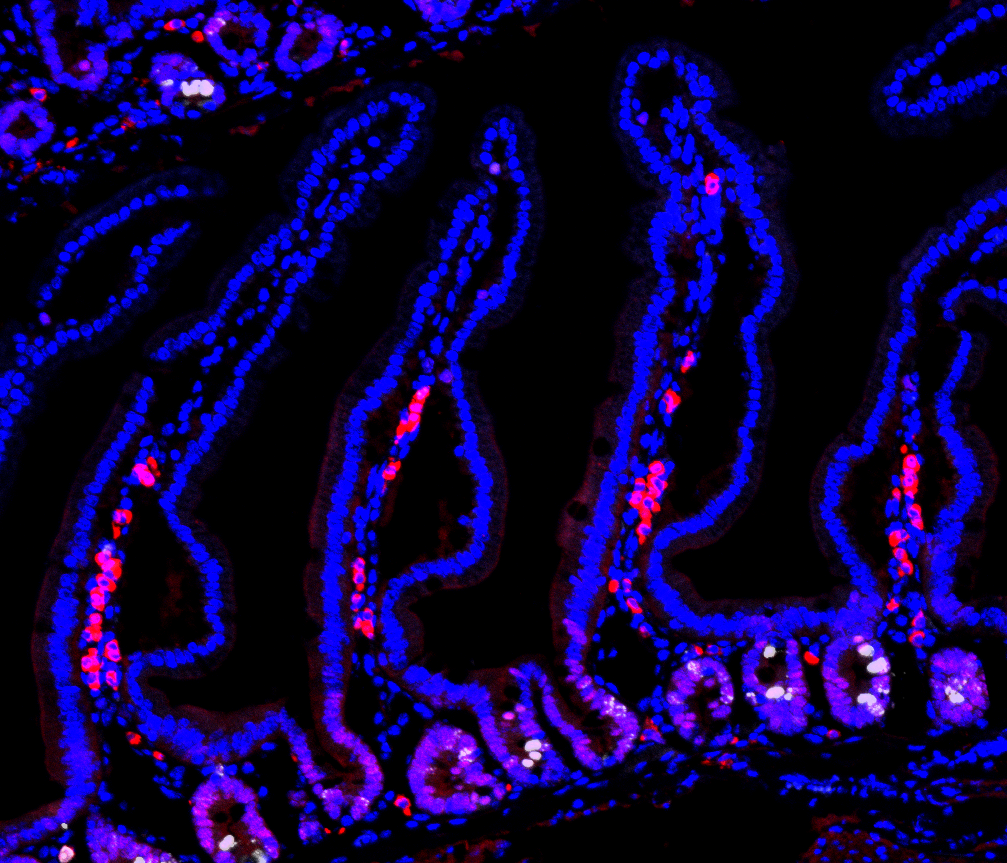

Supplement: Supplementary file 14 — EV Figure Source data [file 44318_2024_184_MOESM14_ESM.zip › EV figures/Figure EV1/EV1 A/IF pHH3 Ki67 WT NI.tif]

## Slide 1
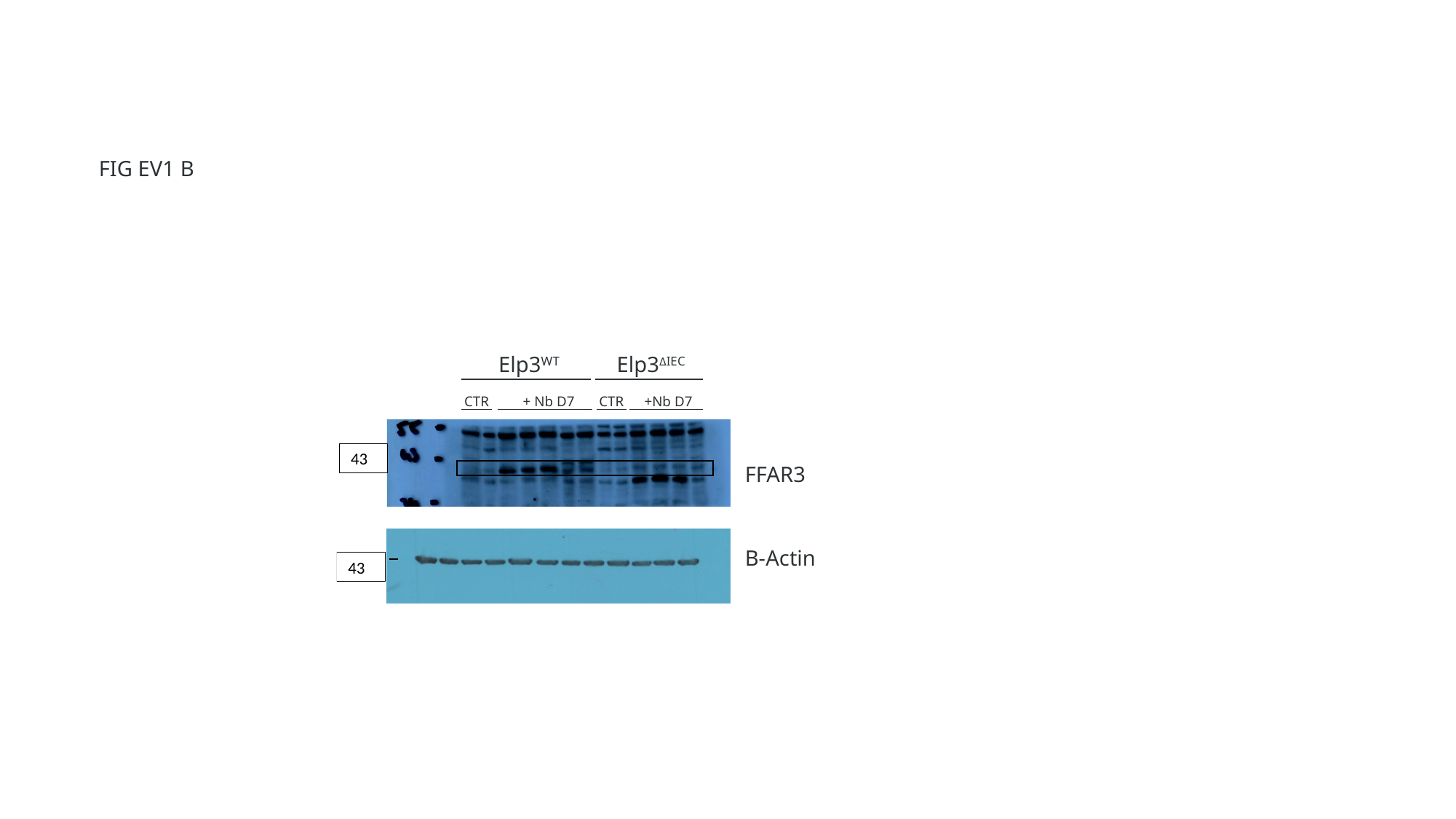

FIG EV1 B
Elp3WT
Elp3ΔIEC
CTR
+ Nb D7
CTR
+Nb D7
FFAR3
B-Actin

Supplement: Supplementary file 14 — EV Figure Source data [file 44318_2024_184_MOESM14_ESM.zip › EV figures/Figure EV1/EV1 B/WB uncropped gels.pptx]

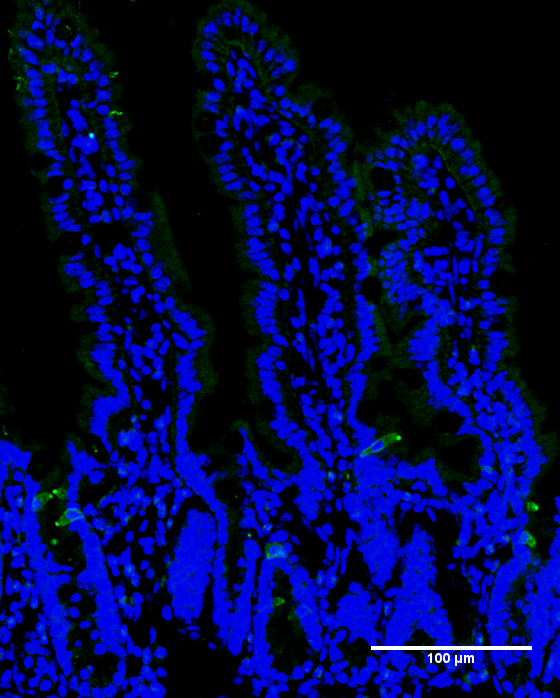

Supplement: Supplementary file 14 — EV Figure Source data [file 44318_2024_184_MOESM14_ESM.zip › EV figures/Figure EV2/EV2 A/IF Dclk1 pSTAT6 KO D7 2 (RGB) green.tif]

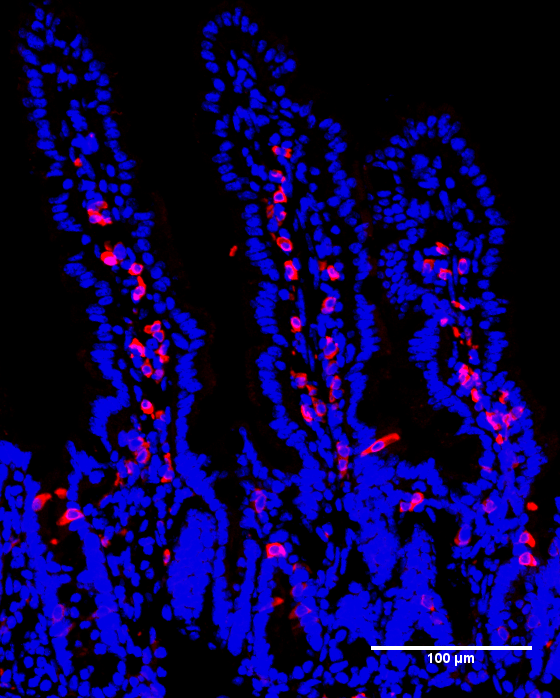

Supplement: Supplementary file 14 — EV Figure Source data [file 44318_2024_184_MOESM14_ESM.zip › EV figures/Figure EV2/EV2 A/IF Dclk1 pSTAT6 KO D7 2 (RGB) red.tif]

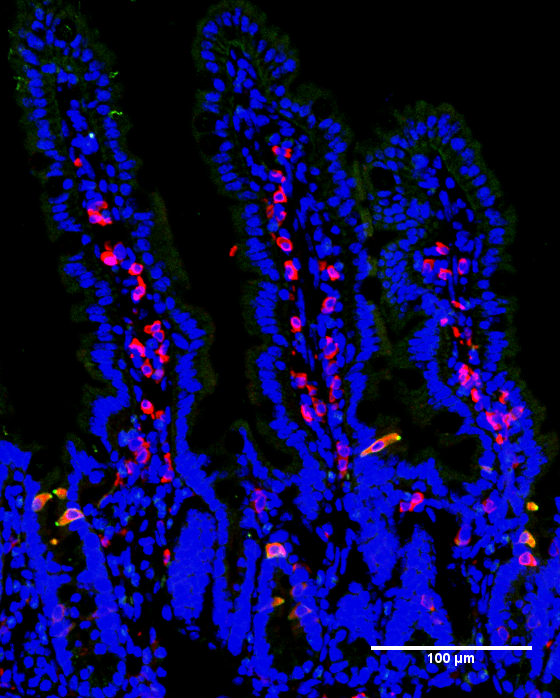

Supplement: Supplementary file 14 — EV Figure Source data [file 44318_2024_184_MOESM14_ESM.zip › EV figures/Figure EV2/EV2 A/IF Dclk1 pSTAT6 KO D7 2 (RGB).tif]

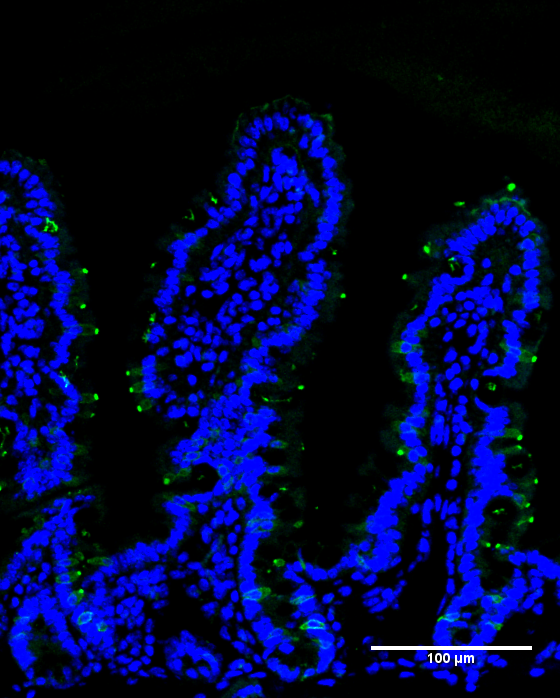

Supplement: Supplementary file 14 — EV Figure Source data [file 44318_2024_184_MOESM14_ESM.zip › EV figures/Figure EV2/EV2 A/IF Dclk1 pSTAT6 WT D7 4 (RGB) green.tif]

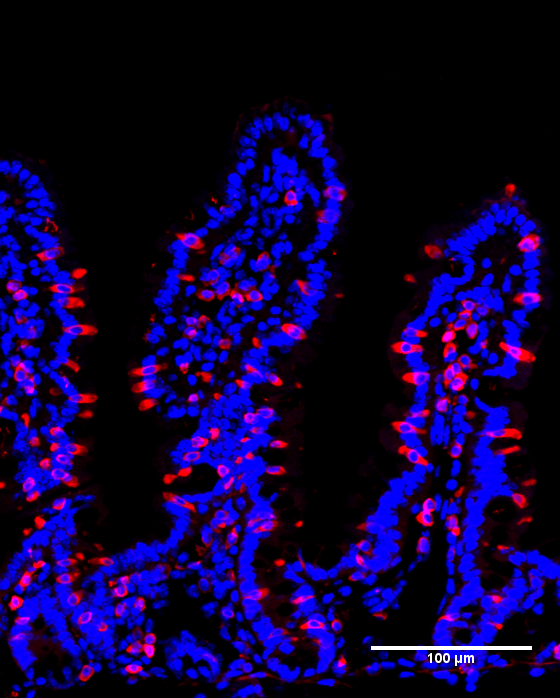

Supplement: Supplementary file 14 — EV Figure Source data [file 44318_2024_184_MOESM14_ESM.zip › EV figures/Figure EV2/EV2 A/IF Dclk1 pSTAT6 WT D7 4 (RGB) red.tif]

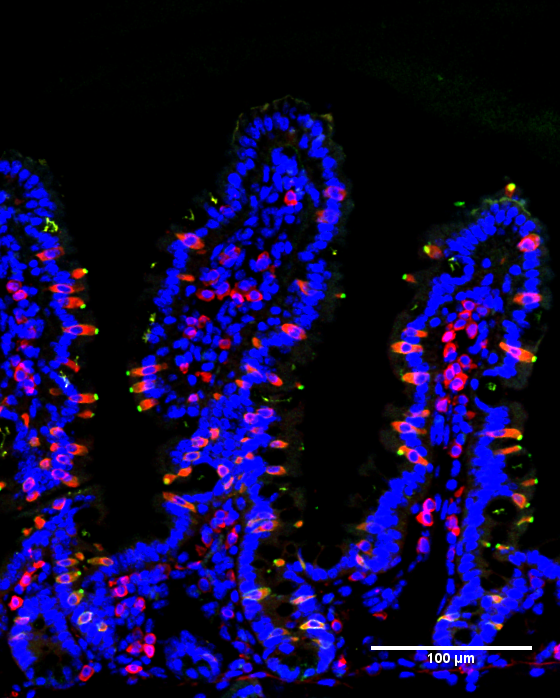

Supplement: Supplementary file 14 — EV Figure Source data [file 44318_2024_184_MOESM14_ESM.zip › EV figures/Figure EV2/EV2 A/IF Dclk1 pSTAT6 WT D7 4 (RGB).tif]

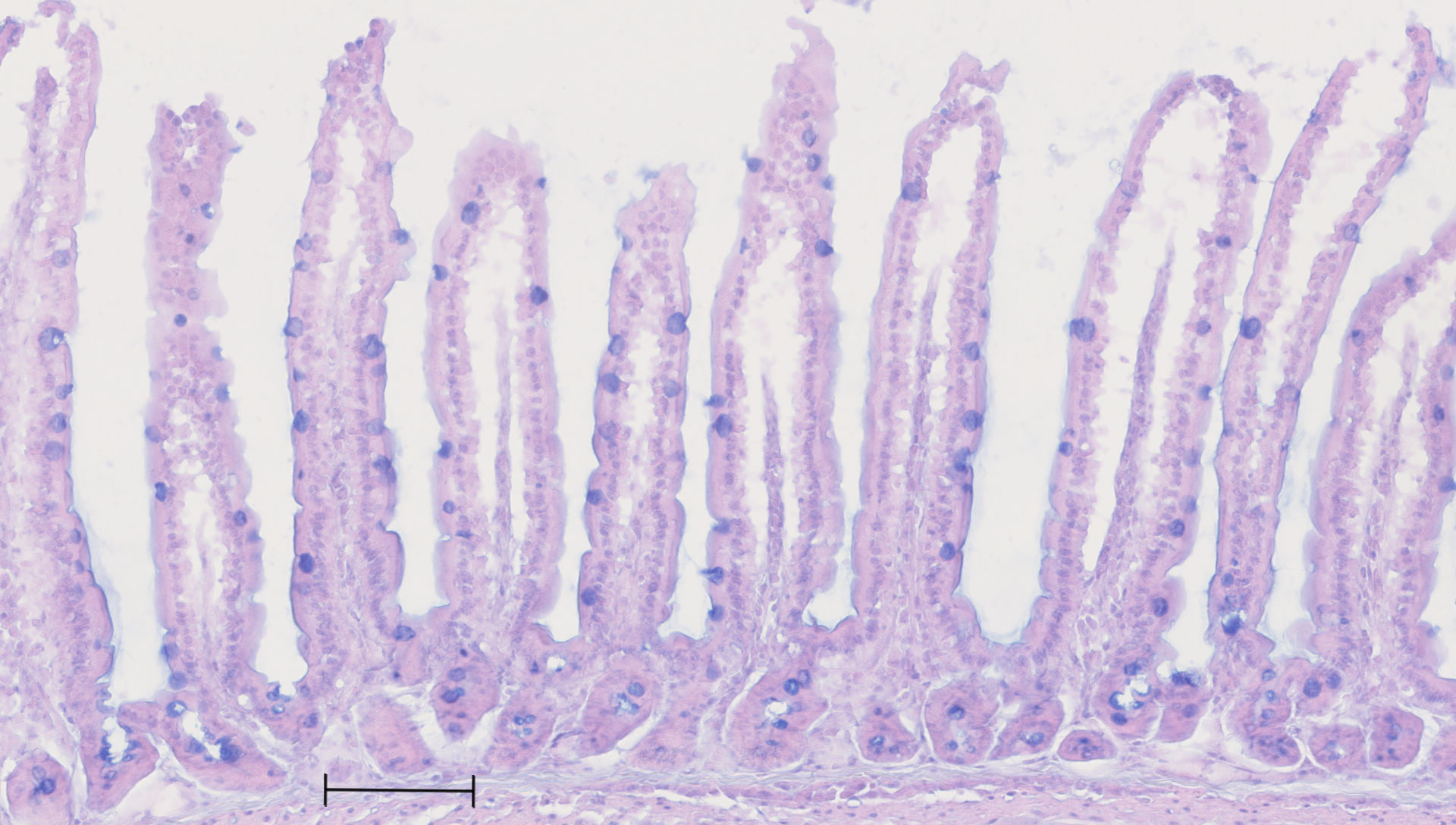

Supplement: Supplementary file 14 — EV Figure Source data [file 44318_2024_184_MOESM14_ESM.zip › EV figures/Figure EV2/EV2 B/IHC Alcian Blue KO IL13.jpg]

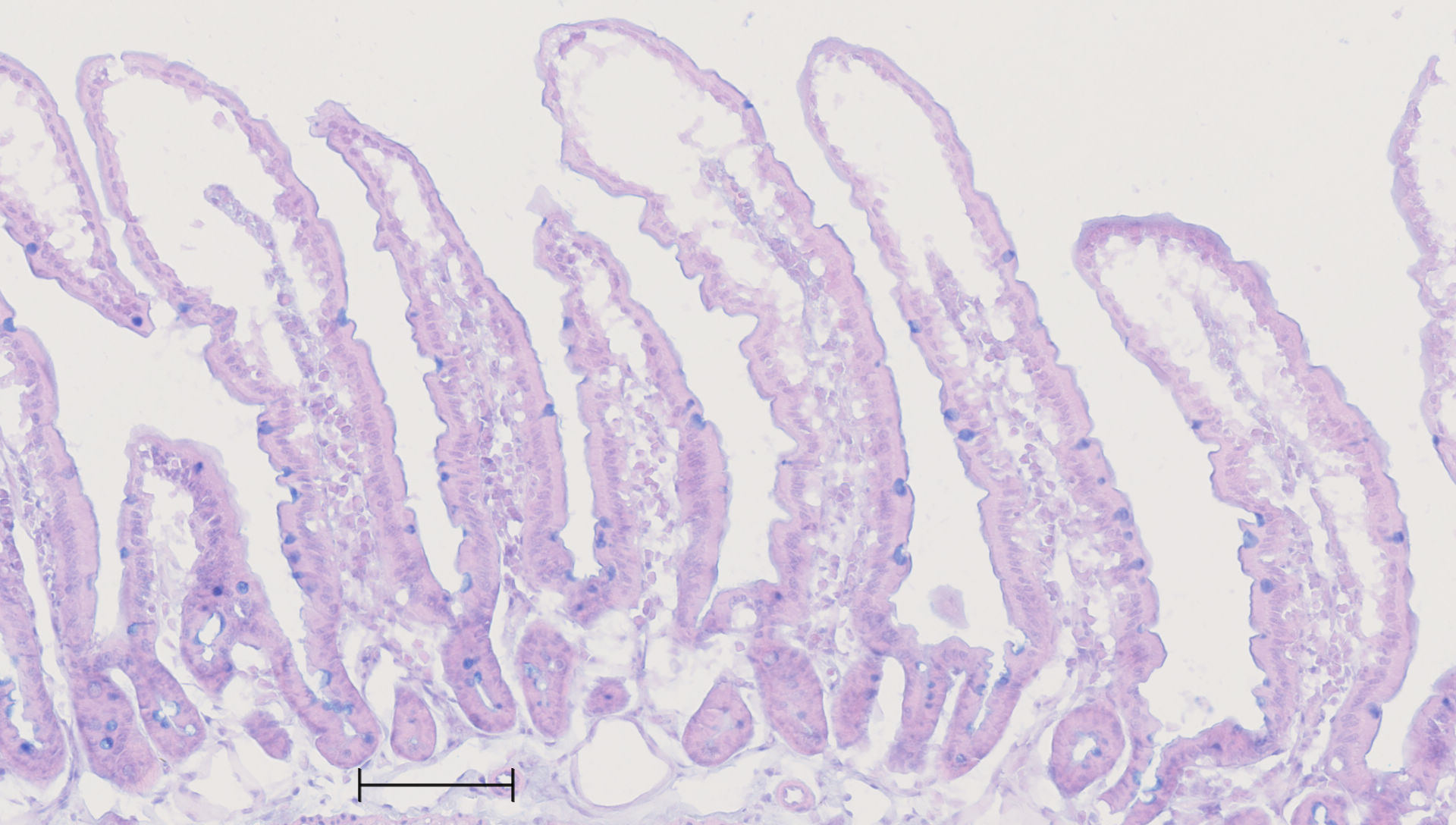

Supplement: Supplementary file 14 — EV Figure Source data [file 44318_2024_184_MOESM14_ESM.zip › EV figures/Figure EV2/EV2 B/IHC Alcian Blue KO PBS.jpg]

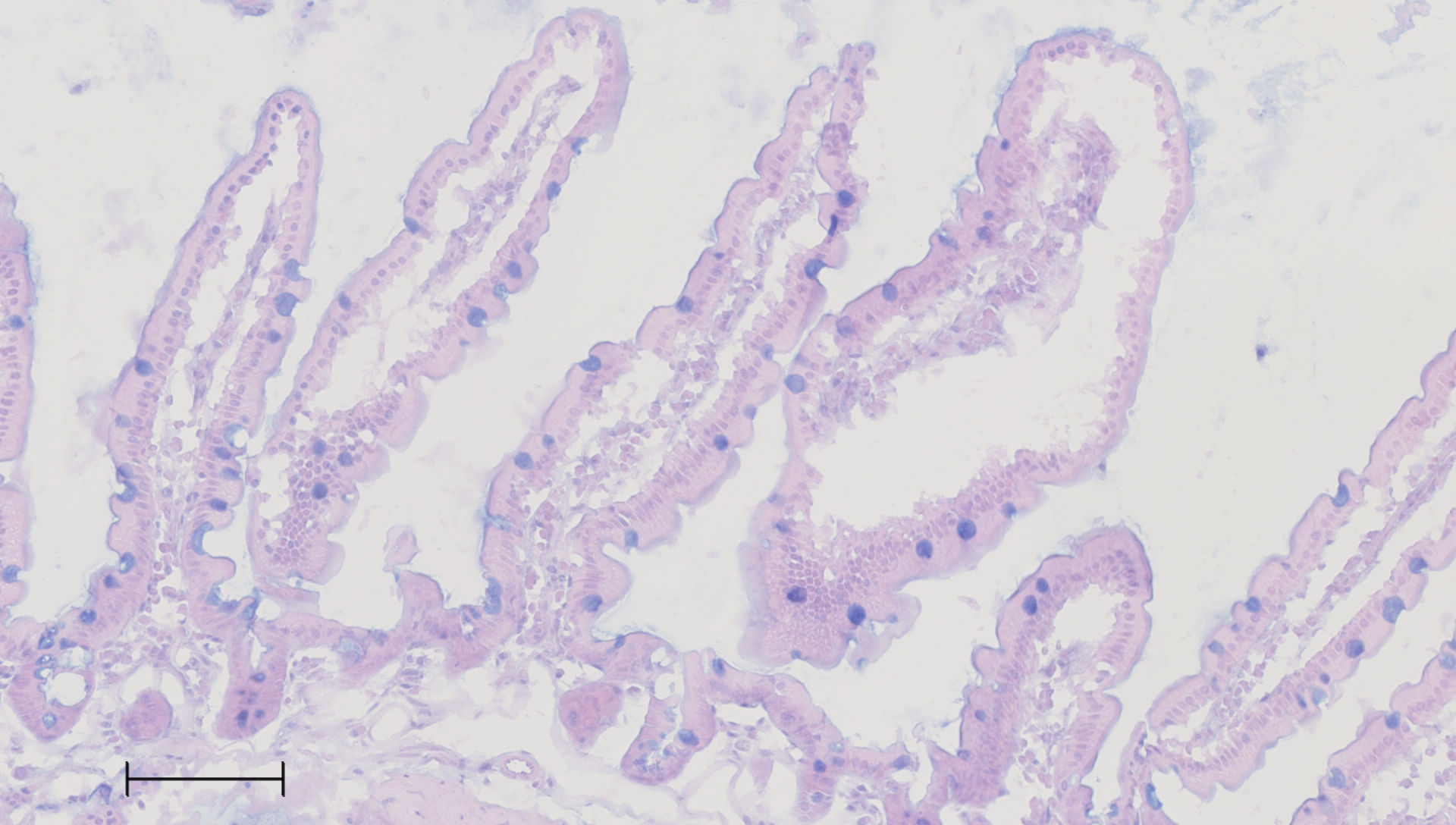

Supplement: Supplementary file 14 — EV Figure Source data [file 44318_2024_184_MOESM14_ESM.zip › EV figures/Figure EV2/EV2 B/IHC Alcian Blue WT IL13.jpg]

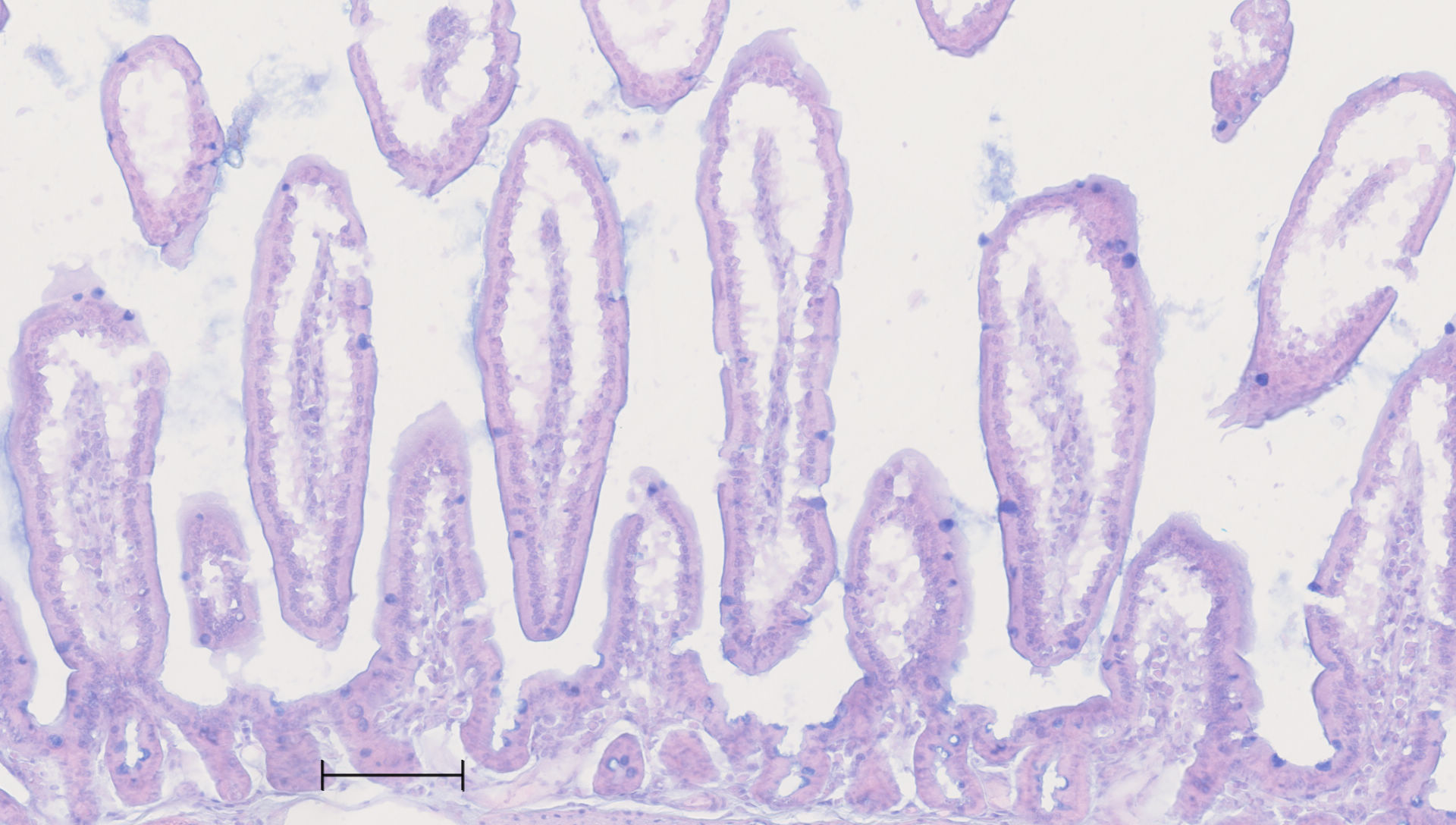

Supplement: Supplementary file 14 — EV Figure Source data [file 44318_2024_184_MOESM14_ESM.zip › EV figures/Figure EV2/EV2 B/IHC Alcian Blue WT PBS.jpg]

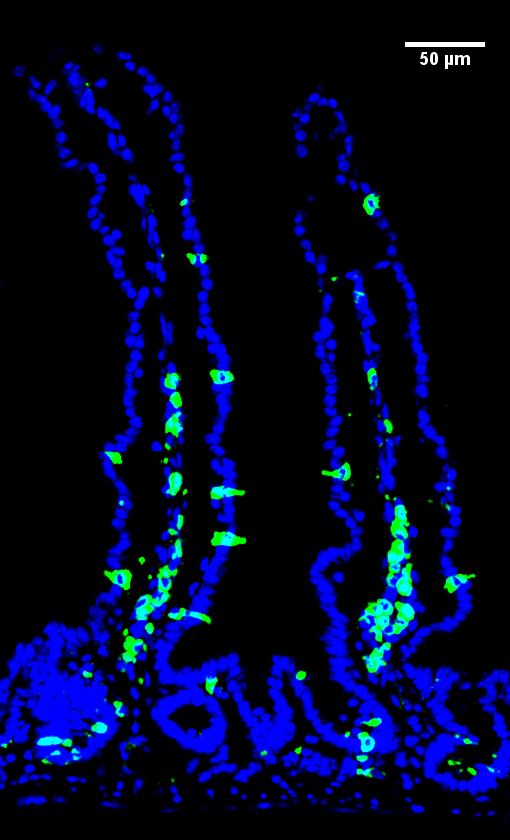

Supplement: Supplementary file 14 — EV Figure Source data [file 44318_2024_184_MOESM14_ESM.zip › EV figures/Figure EV2/EV2 C/IF ChgA KO DBZ.tif]

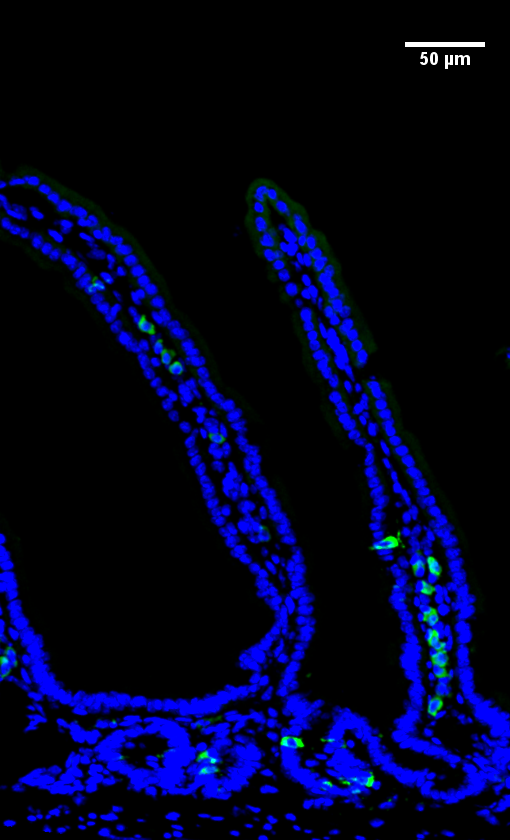

Supplement: Supplementary file 14 — EV Figure Source data [file 44318_2024_184_MOESM14_ESM.zip › EV figures/Figure EV2/EV2 C/IF ChgA KO PBS.tif]

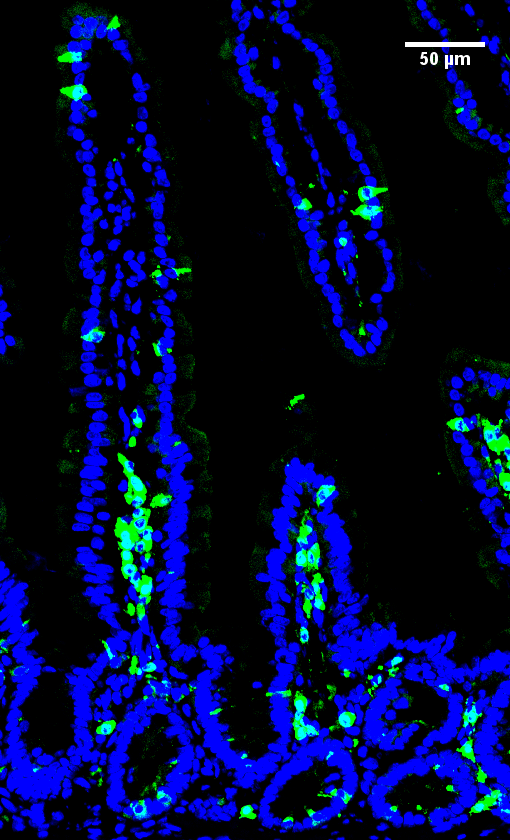

Supplement: Supplementary file 14 — EV Figure Source data [file 44318_2024_184_MOESM14_ESM.zip › EV figures/Figure EV2/EV2 C/IF ChgA WT DBZ.tif]

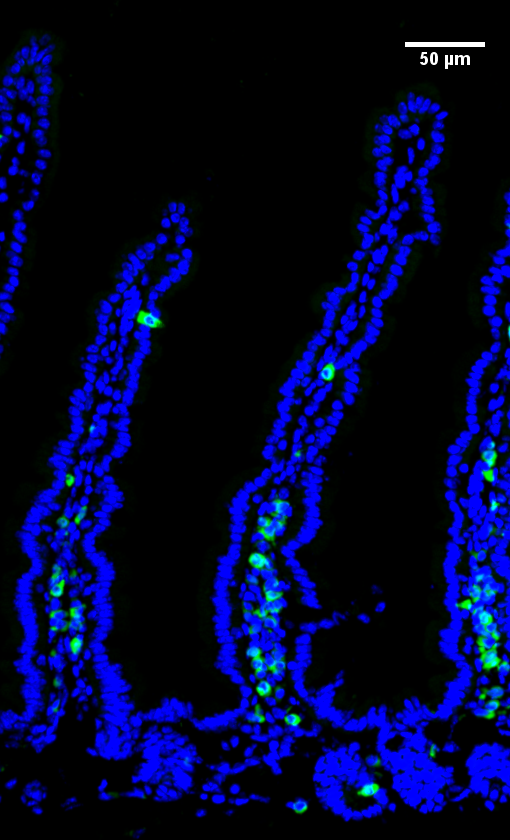

Supplement: Supplementary file 14 — EV Figure Source data [file 44318_2024_184_MOESM14_ESM.zip › EV figures/Figure EV2/EV2 C/IF ChgA WT PBS.tif]

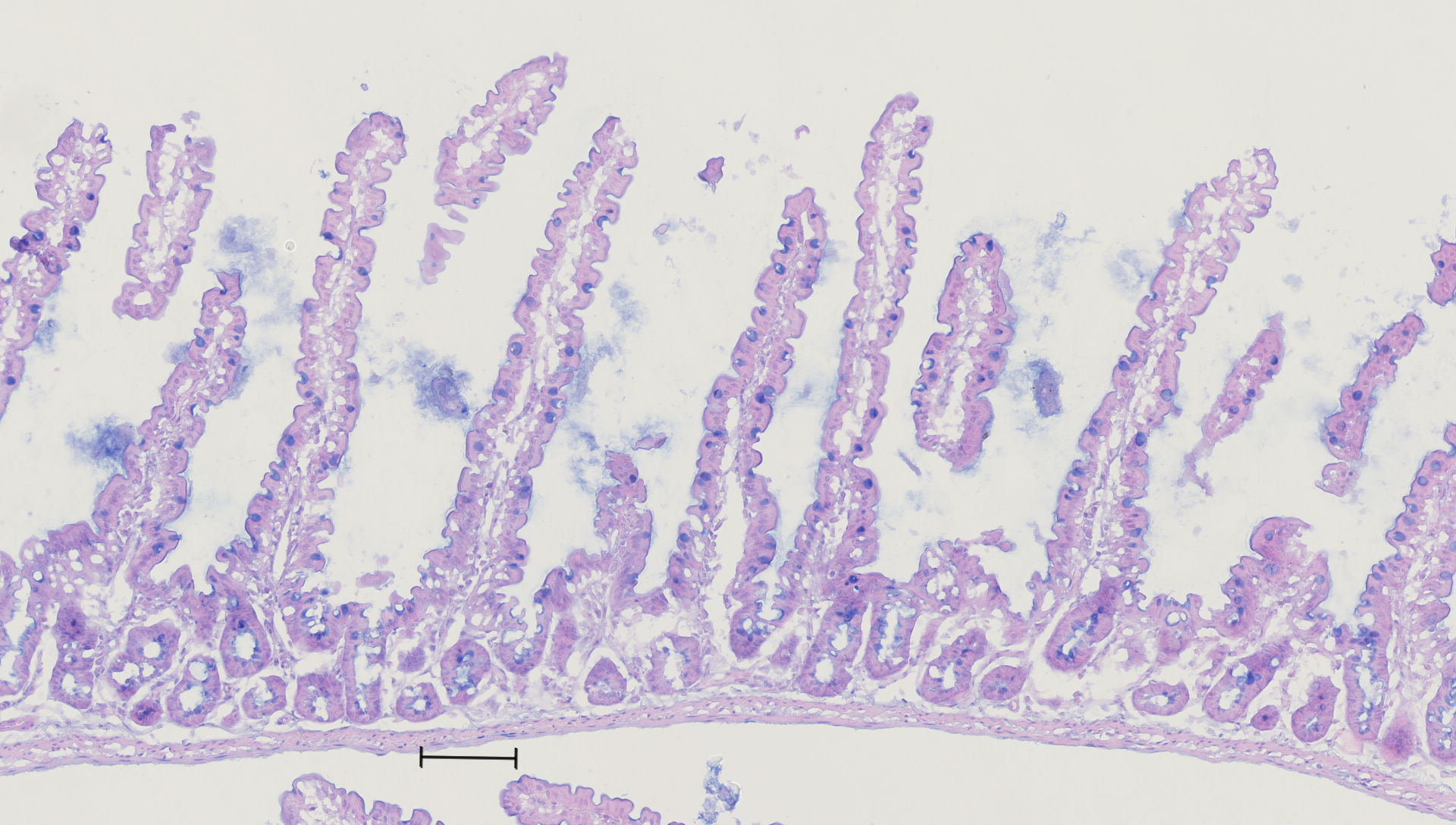

Supplement: Supplementary file 14 — EV Figure Source data [file 44318_2024_184_MOESM14_ESM.zip › EV figures/Figure EV2/EV2 D/IHC Alcian Blue KO DBZ.tiff]

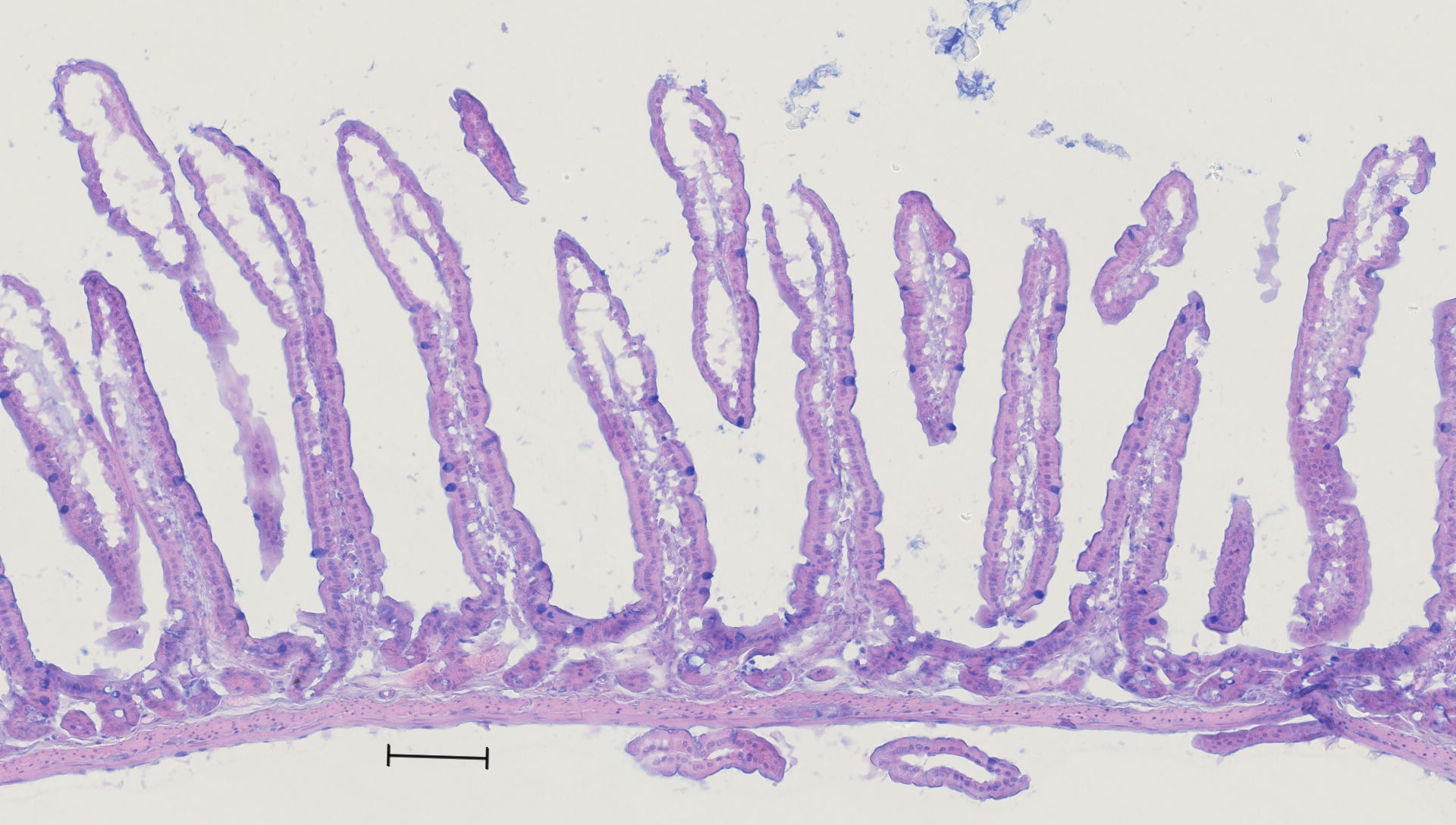

Supplement: Supplementary file 14 — EV Figure Source data [file 44318_2024_184_MOESM14_ESM.zip › EV figures/Figure EV2/EV2 D/IHC Alcian Blue KO Veh.jpg]

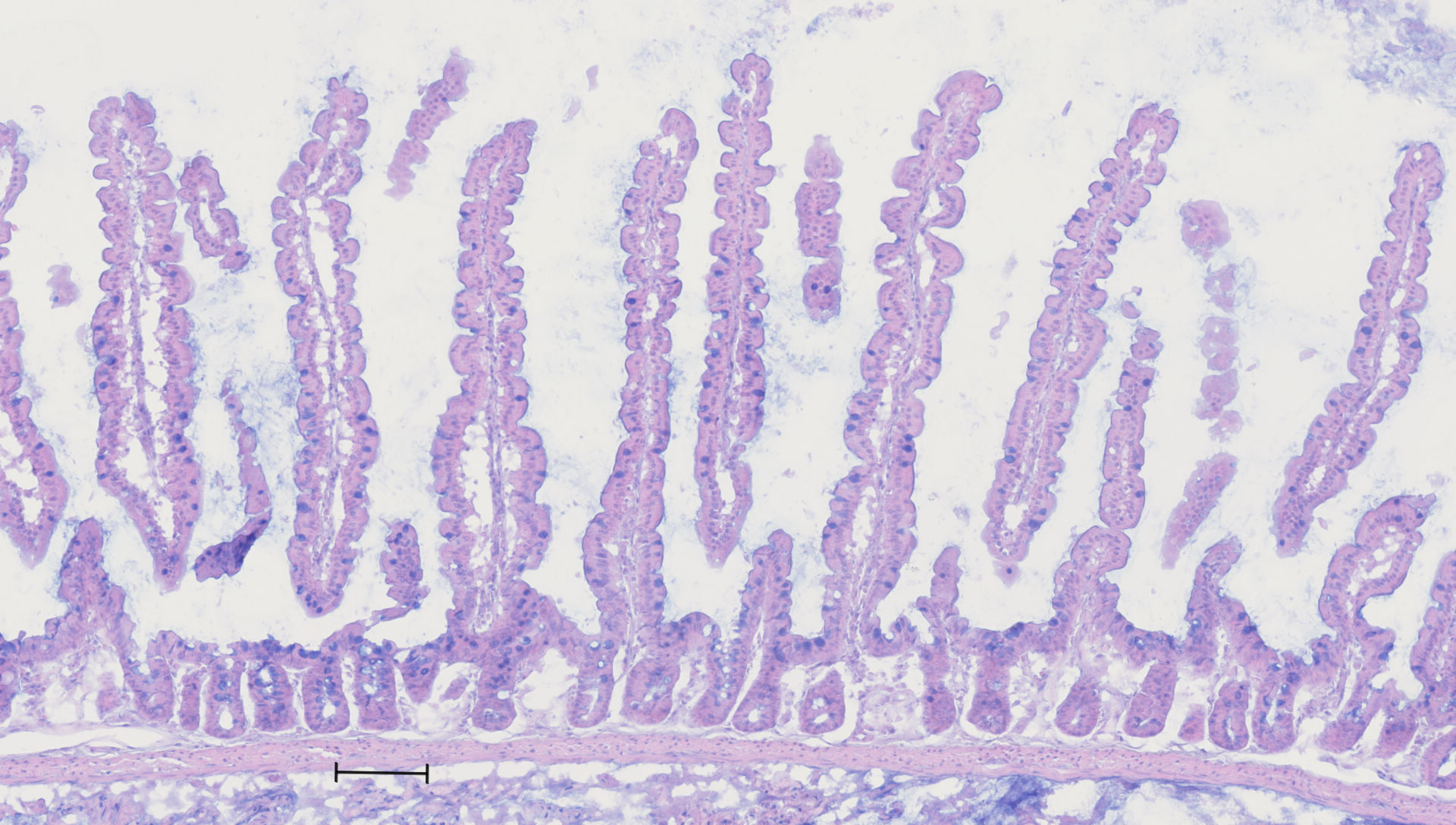

Supplement: Supplementary file 14 — EV Figure Source data [file 44318_2024_184_MOESM14_ESM.zip › EV figures/Figure EV2/EV2 D/IHC Alcian Blue WT DBZ.jpg]

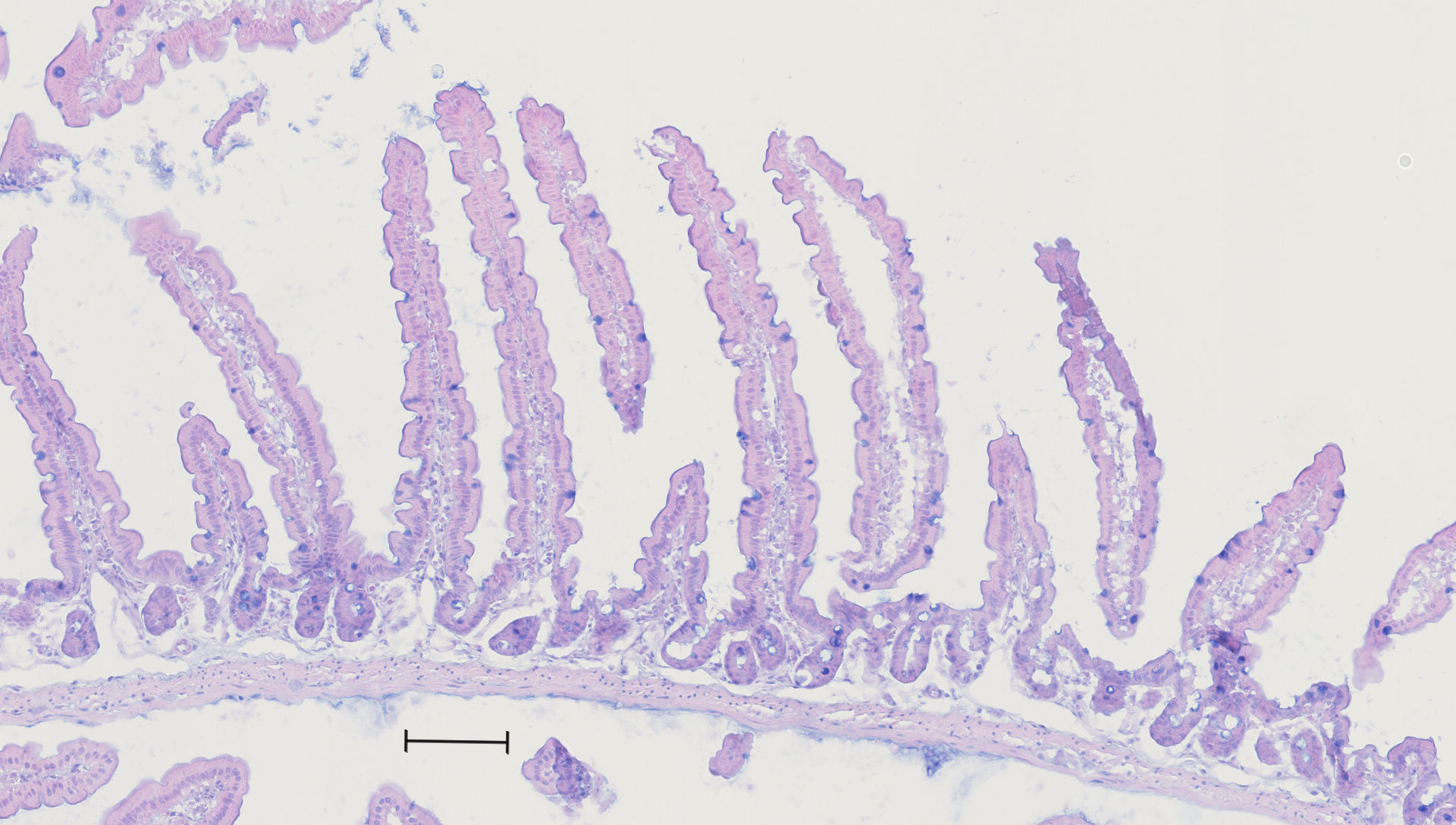

Supplement: Supplementary file 14 — EV Figure Source data [file 44318_2024_184_MOESM14_ESM.zip › EV figures/Figure EV2/EV2 D/IHC Alcian Blue WT Veh.tiff]

## Slide 1
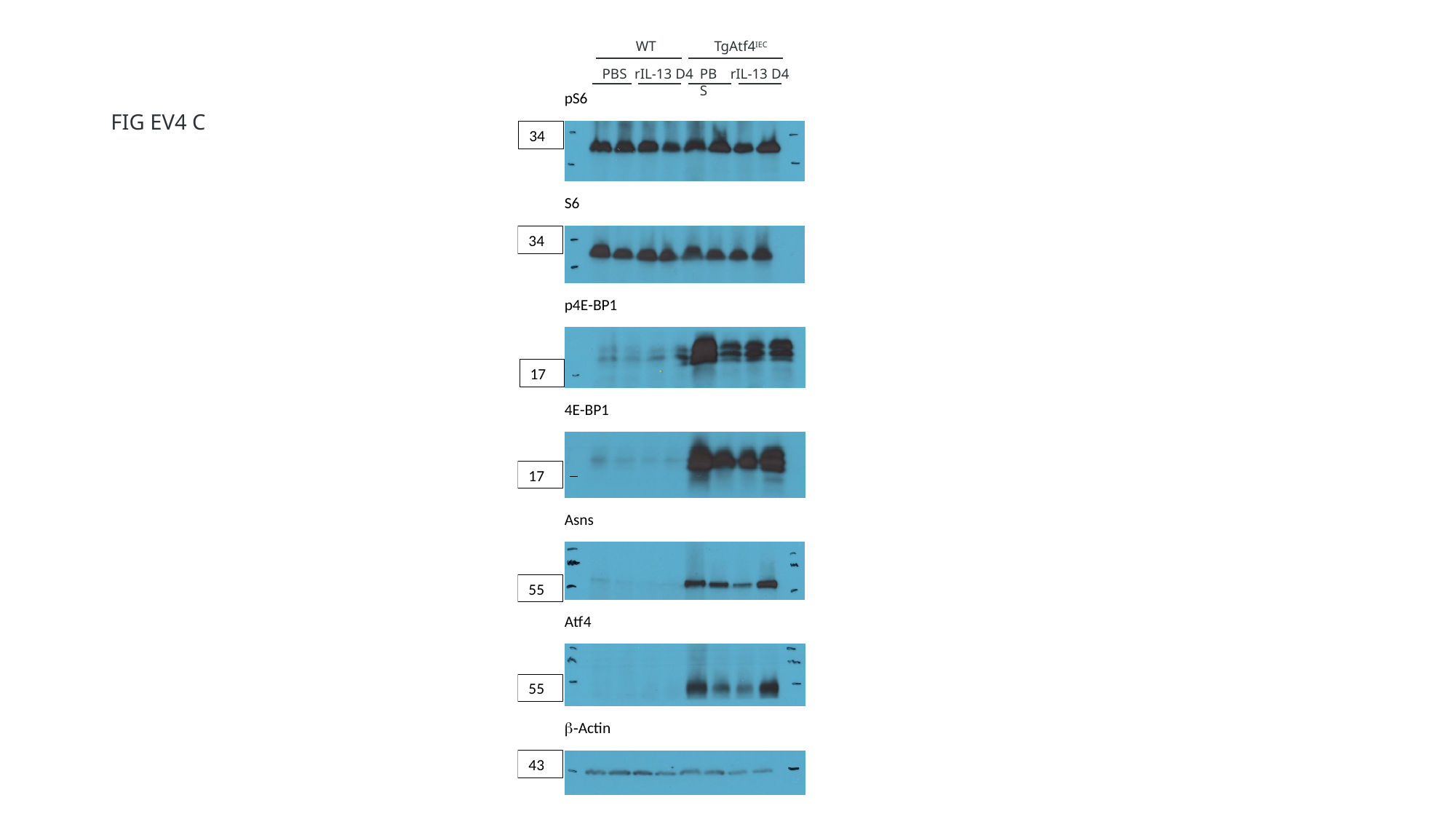

WT
TgAtf4IEC
PBS
rIL-13 D4
PBS
rIL-13 D4
# FIG EV4 C

Supplement: Supplementary file 14 — EV Figure Source data [file 44318_2024_184_MOESM14_ESM.zip › EV figures/Figure EV4/EV4 C/WB uncropped gels.pptx]

## Slide 1
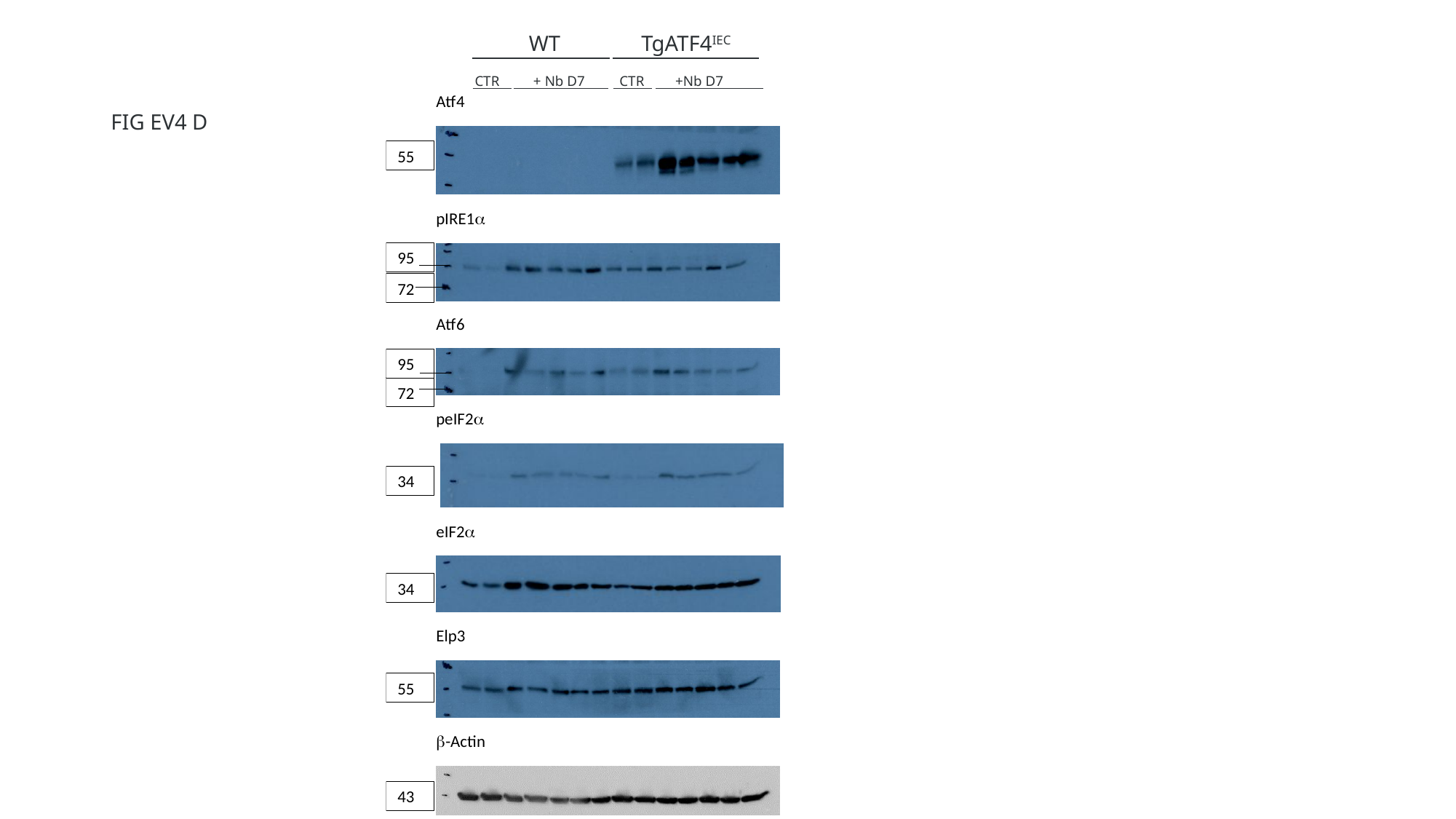

WT
TgATF4IEC
CTR
+ Nb D7
CTR
+Nb D7
# FIG EV4 D

Supplement: Supplementary file 14 — EV Figure Source data [file 44318_2024_184_MOESM14_ESM.zip › EV figures/Figure EV4/EV4 D/WB uncropped gels.pptx]

## Slide 1
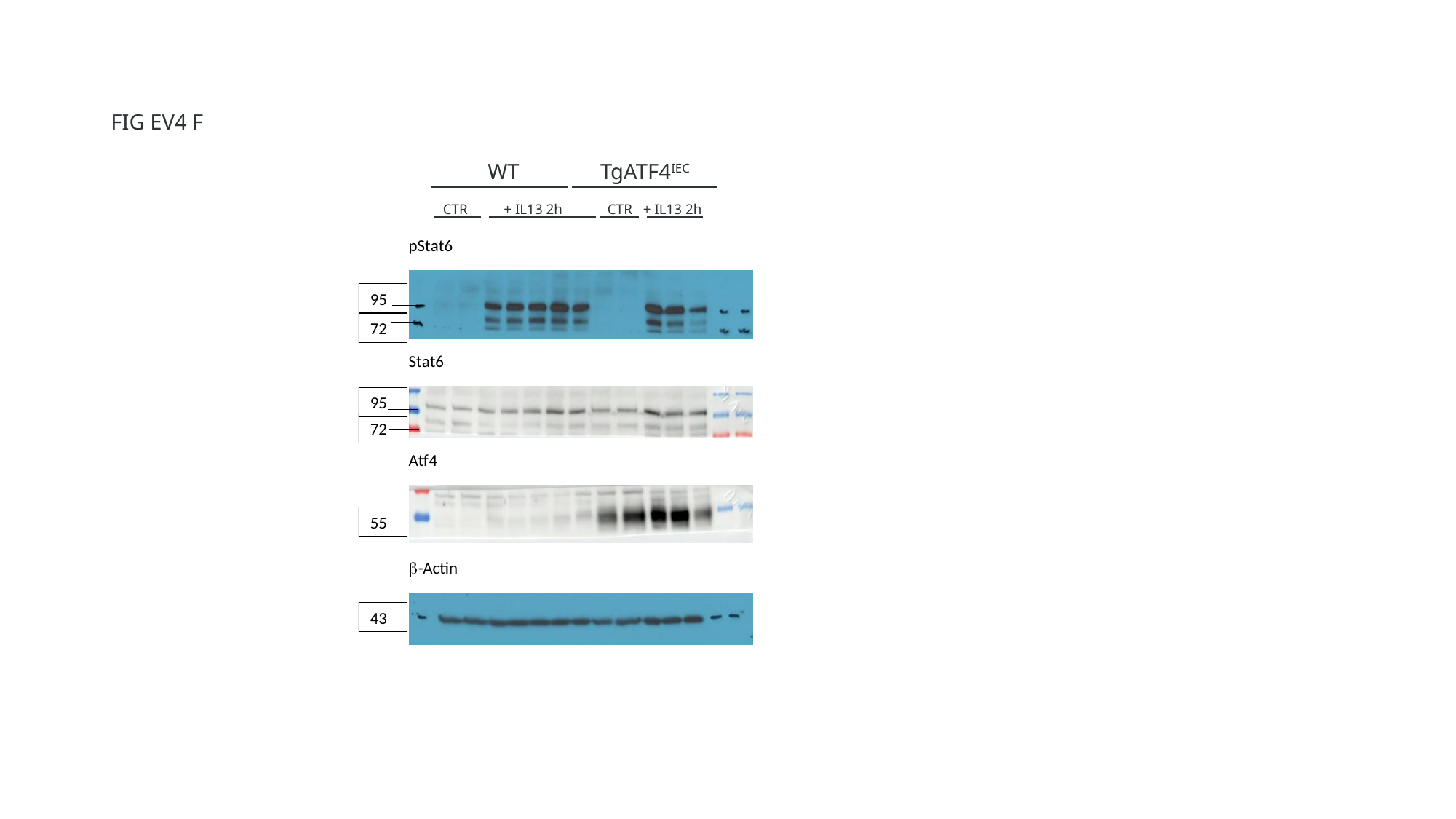

# FIG EV4 F
WT
TgATF4IEC
CTR
+ IL13 2h
CTR
+ IL13 2h

Supplement: Supplementary file 14 — EV Figure Source data [file 44318_2024_184_MOESM14_ESM.zip › EV figures/Figure EV4/EV4 F/WB uncropped gels.pptx]

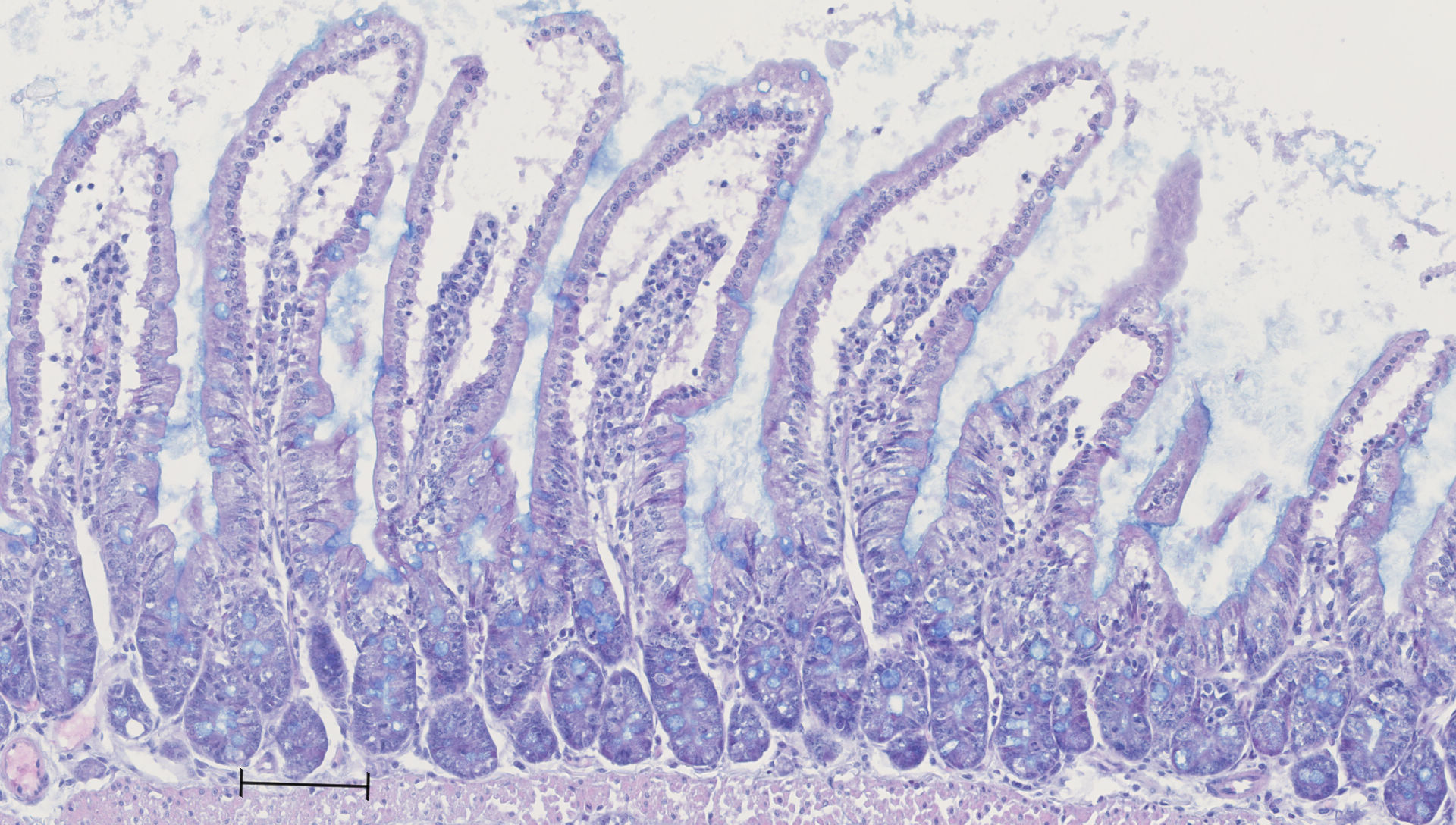

Supplement: Supplementary file 14 — EV Figure Source data [file 44318_2024_184_MOESM14_ESM.zip › EV figures/Figure EV4/EV4 G/IHC Alcian Blue TgAtf4 Il13 b.jpg]

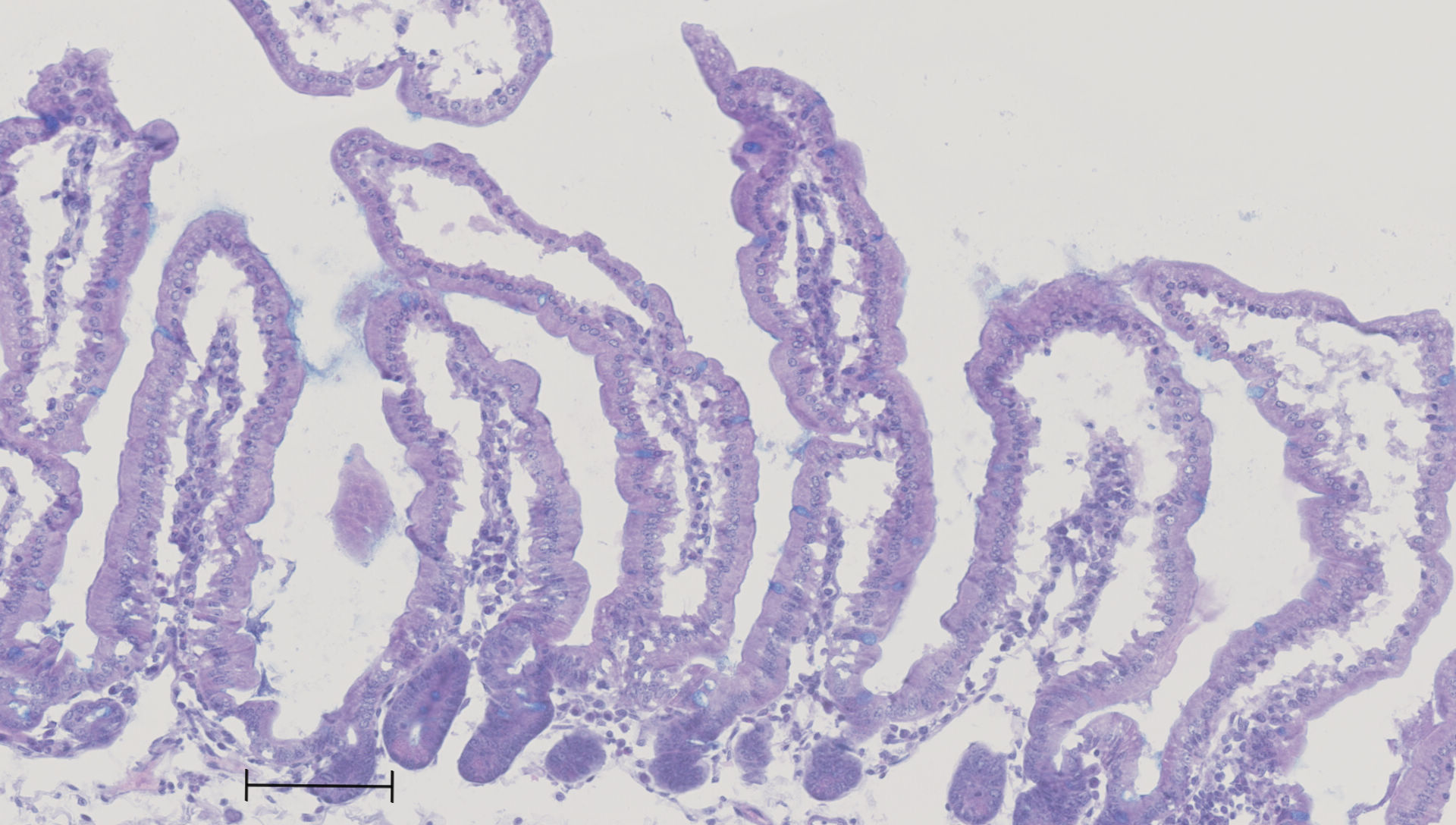

Supplement: Supplementary file 14 — EV Figure Source data [file 44318_2024_184_MOESM14_ESM.zip › EV figures/Figure EV4/EV4 G/IHC Alcian Blue TgATf4 PBS.jpg]

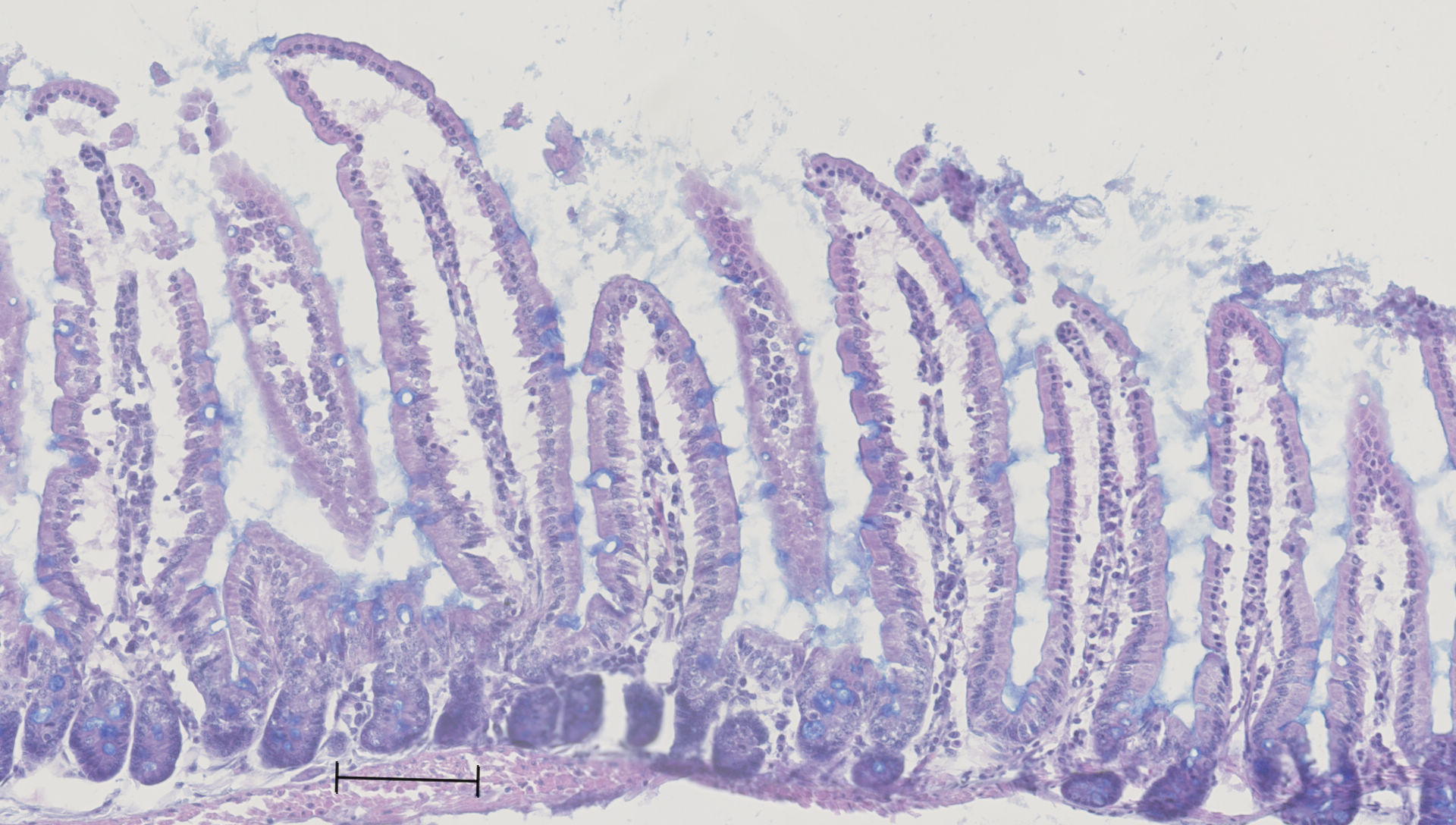

Supplement: Supplementary file 14 — EV Figure Source data [file 44318_2024_184_MOESM14_ESM.zip › EV figures/Figure EV4/EV4 G/IHC Alcian Blue WT IL13.jpg]

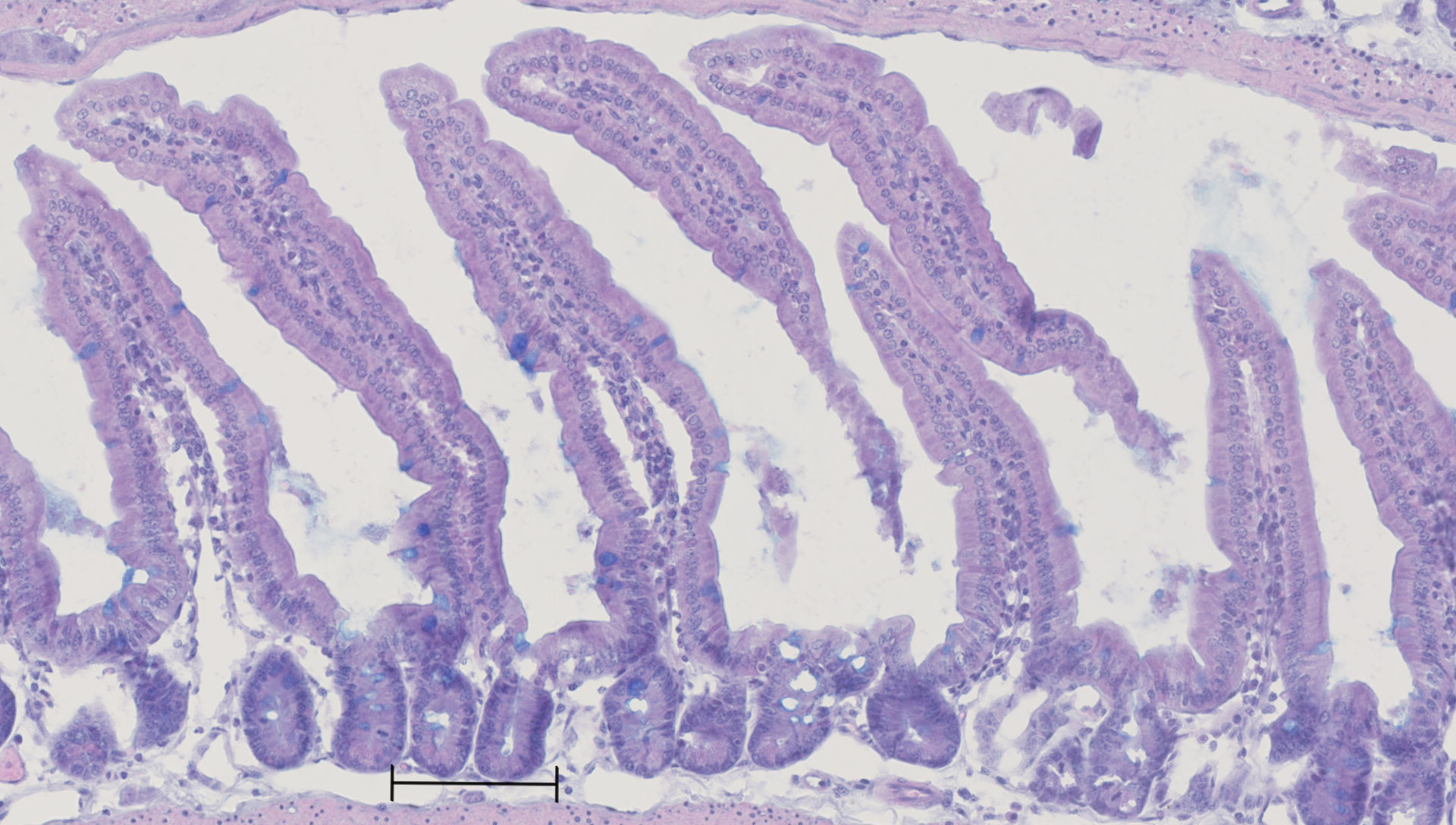

Supplement: Supplementary file 14 — EV Figure Source data [file 44318_2024_184_MOESM14_ESM.zip › EV figures/Figure EV4/EV4 G/IHC Alcian Blue WT PBS.jpg]

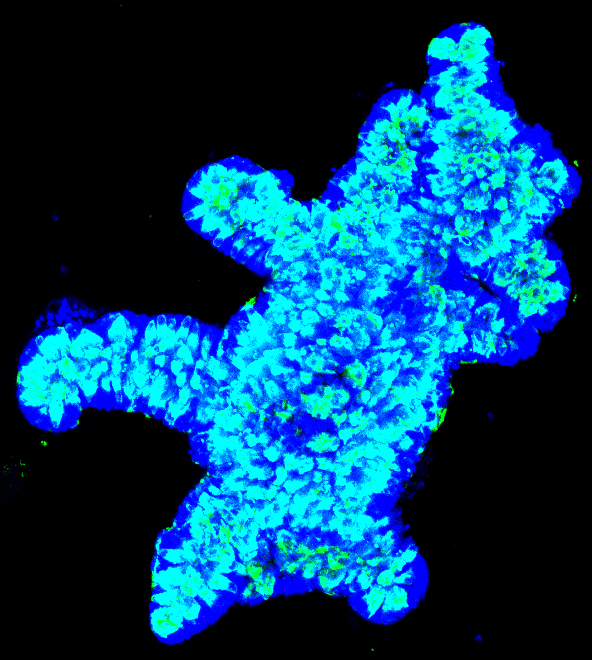

Supplement: Supplementary file 14 — EV Figure Source data [file 44318_2024_184_MOESM14_ESM.zip › EV figures/Figure EV4/EV4 J/Mucin2 IF TgATF4 + IL13.tif]

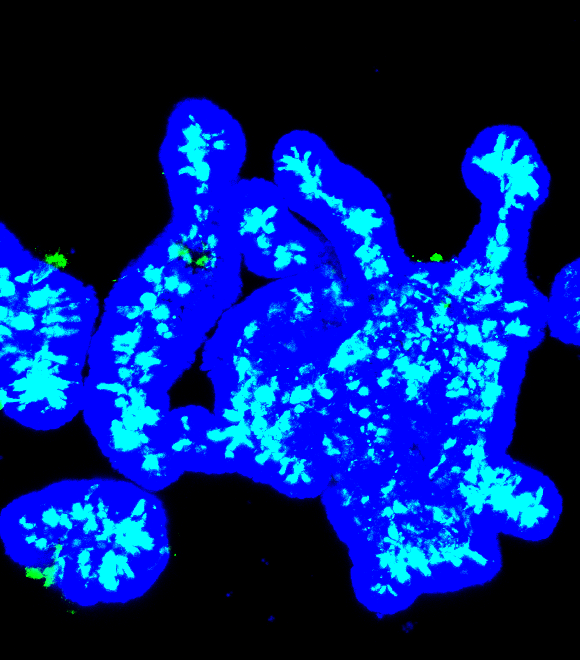

Supplement: Supplementary file 14 — EV Figure Source data [file 44318_2024_184_MOESM14_ESM.zip › EV figures/Figure EV4/EV4 J/Mucin2 IF WT Ctr.tif]

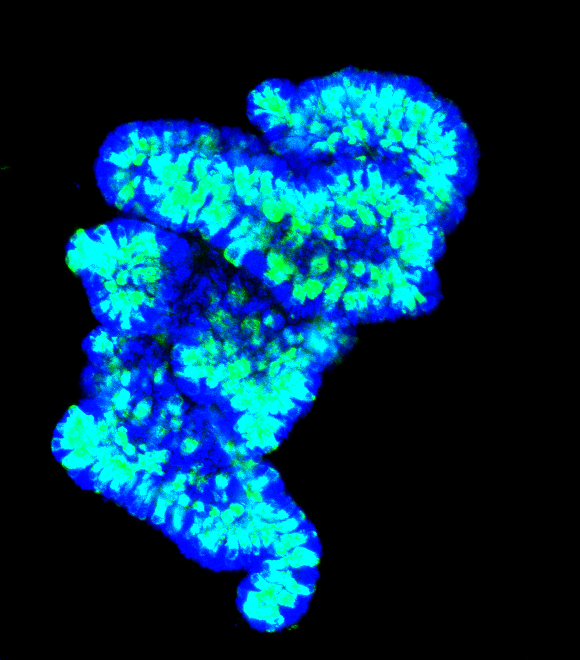

Supplement: Supplementary file 14 — EV Figure Source data [file 44318_2024_184_MOESM14_ESM.zip › EV figures/Figure EV4/EV4 J/Mucin2 IF WT+ IL13.tif]

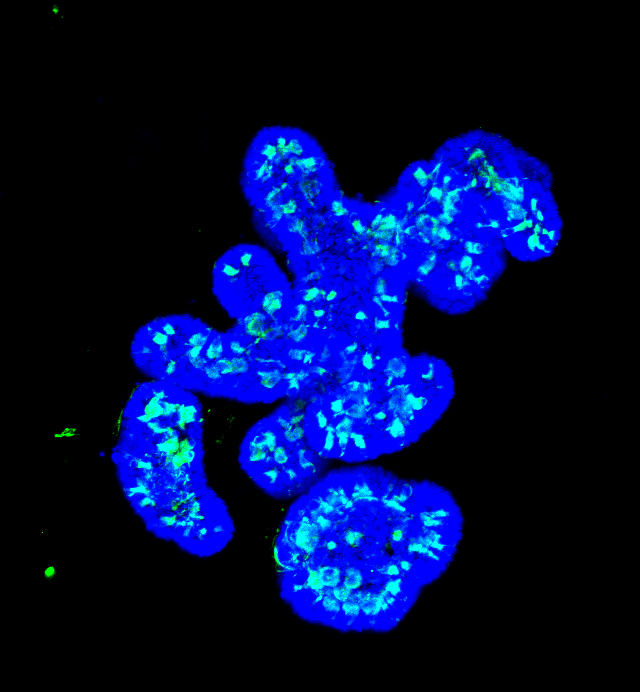

Supplement: Supplementary file 14 — EV Figure Source data [file 44318_2024_184_MOESM14_ESM.zip › EV figures/Figure EV4/EV4 J/Mucin2 TgATF4 Ctr.tif]
