# Supplementary material for: Loss of Elp3 blocks intestinal tuft cell differentiation via an mTORC1-Atf4 axis
Source: EMBO J. 2024 Jul 31;43(18):6. doi: 10.1038/s44318-024-00184-4 (PMC11405396; doi:10.1038/s44318-024-00184-4)
Supplement: Supplementary file 15 — Expanded View Figures [file 44318_2024_184_MOESM15_ESM.pdf]

# Expanded View Figures

**Figure EV1. Intestinal homeostasis is unaffected upon *Elp3* inactivation.**

(**A**) Intestines lacking Elp3 properly proliferate. Immunolabelings of intestinal sections from mice of the indicated genotypes and infected or not with *N. brasiliensis* are illustrated (KI67 and pHH3 stainings are in red and white, respectively). On the right, histograms show corresponding quantifications ($n = 5$ mice per genotype, mean value ± SEM; Mann–Whitney test shows no significant difference between $Elp3^{WT}$ and $Elp3^{\Delta IEC}$ mice). (**B**) *Elp3* deficiency impairs the induction of FFAR3. Mice of the indicated genotypes were infected with *N. brasiliensis* and protein extracts from intestines 7 days post-infection were subjected to western blot analyses to assess FFAR3 and β-actin expression. (**C**, **D**) Defa5 and ChgA are properly expressed in intestinal epithelial cells lacking Elp3. Mice of the indicated genotypes were infected or not with *N. brasiliensis* and RNAs from the resulting intestines were subjected to real-time PCR analyses to quantify both Defa5 (**C**) and ChgA (**D**) mRNA levels. Expression levels of these candidates were normalized on the average of four housekeeping genes, including Gapdh, β-Actin, 36B4 and β2-Microglobulin ($n \geq 6$ mice per genotype; mean ± SEM; a Mann–Whitney test shows no significant differences between $Elp3^{WT}$ and $Elp3^{\Delta IEC}$ mice). Source data are available online for this figure.


© The Author(s)

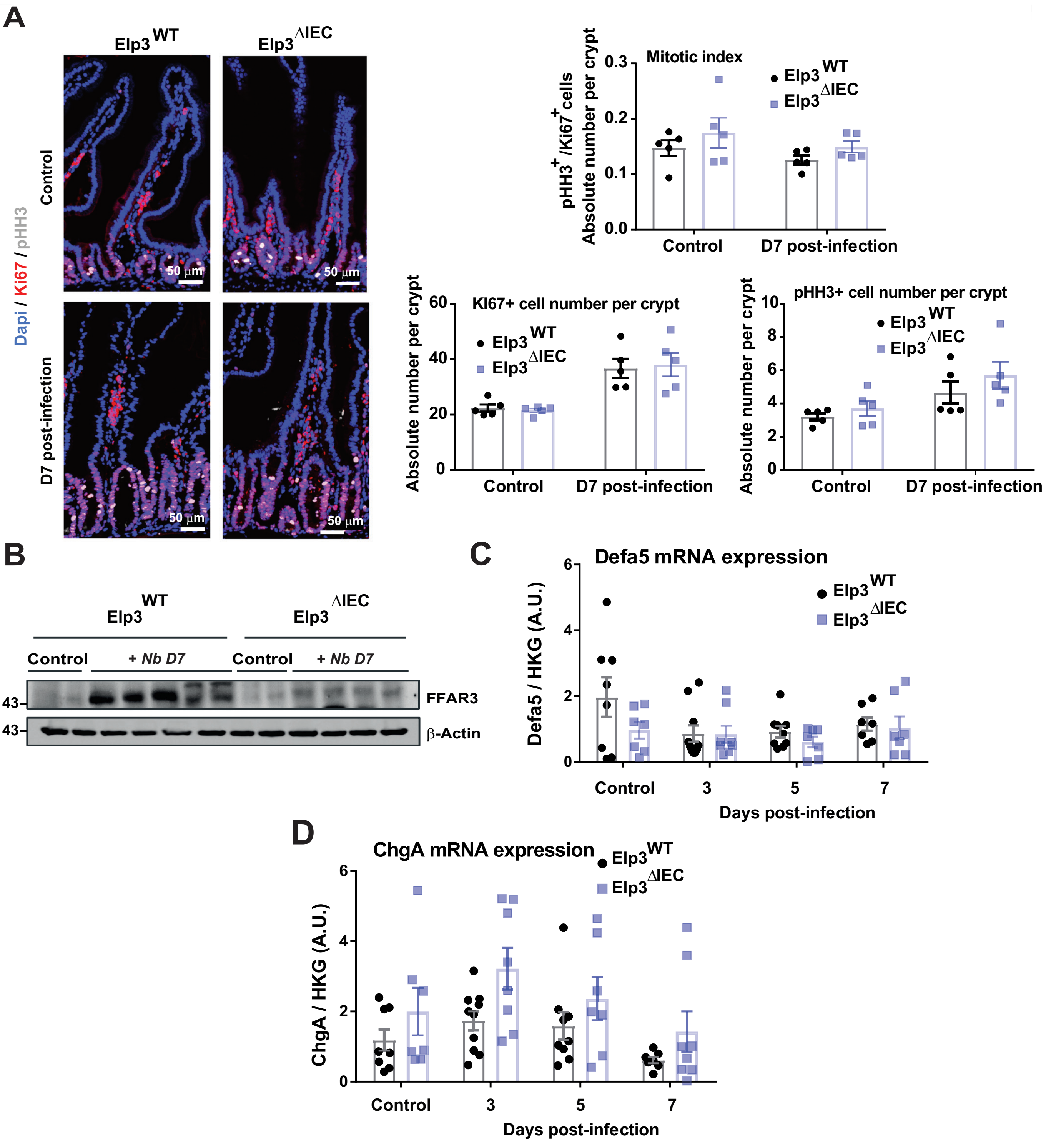
A
Elp3 WT
Elp3 ΔIEC
Dapi / Ki67 / pHH3
Control
D7 post-infection
50 μm
Mitotic index
pHH3+ /Ki67+ cells
Absolute number per crypt
Elp3 WT
Elp3 ΔIEC
Control
D7 post-infection
KI67+ cell number per crypt
pHH3+ cell number per crypt
B
Control
+ Nb D7
FFAR3
β-Actin
43
C
Defa5 mRNA expression
Defa5 / HKG (A.U.)
Days post-infection
D
ChgA mRNA expression
ChgA / HKG (A.U.)

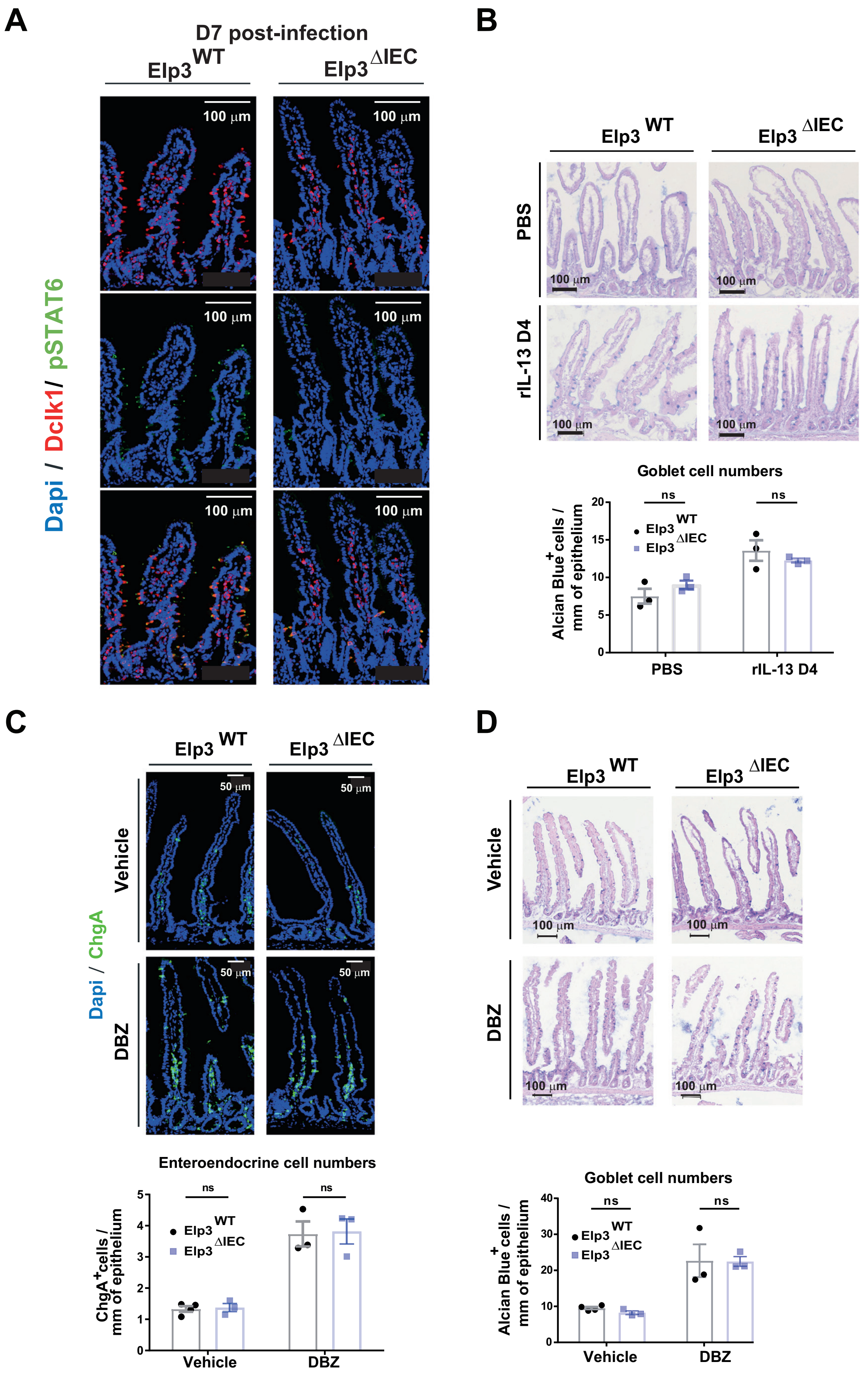
A
D7 post-infection
Elp3 WT
Elp3 ΔIEC
100 μm
Dapi / Dclk1/ pSTAT6
B
Elp3 WT
Elp3 ΔIEC
PBS
rIL-13 D4
100 μm
Goblet cell numbers
ns
Alcian Blue+ cells / mm of epithelium
PBS
rIL-13 D4
C
Elp3 WT
Elp3 ΔIEC
Vehicle
DBZ
50 μm
Dapi / ChgA
Enteroendocrine cell numbers
ChgA+cells / mm of epithelium
D
Elp3 WT
Elp3 ΔIEC
Vehicle
DBZ
100 μm
Goblet cell numbers
Alcian Blue+ cells / mm of epithelium

◀ **Figure EV2. Elp3 is dispensable for the expansion of both enteroendocrine and goblet cells.**

(**A**) Mice of the indicated genotypes were infected with *N. brasiliensis* and immunofluorescence analyses in the intestine were conducted 7 days post-infection to detect tuft cells (Dclk1$^{+}$ cells in red) as well as Stat6 phosphorylation (in green). (**B–D**) Mice of the indicated genotypes were treated or not with recombinant IL-13 (rIL-13) for 4 days (**B**) or with the Notch inhibitor DBZ (**C**, **D**), and the resulting intestines were subjected to immunohistochemistry analyses to quantify goblet cells (Alcian Blue$^{+}$ cells) (**B**, **D**) or enteroendocrine cells (Chromogranin A$^{+}$ cells (**C**) (top panels). At the bottom, a quantification is provided (mean values ± SEM; Mann–Whitney test, $n = 3$ and $n \geq 3$ for IL-13 and DBZ treatments, respectively). Source data are available online for this figure.

© The Author(s) 

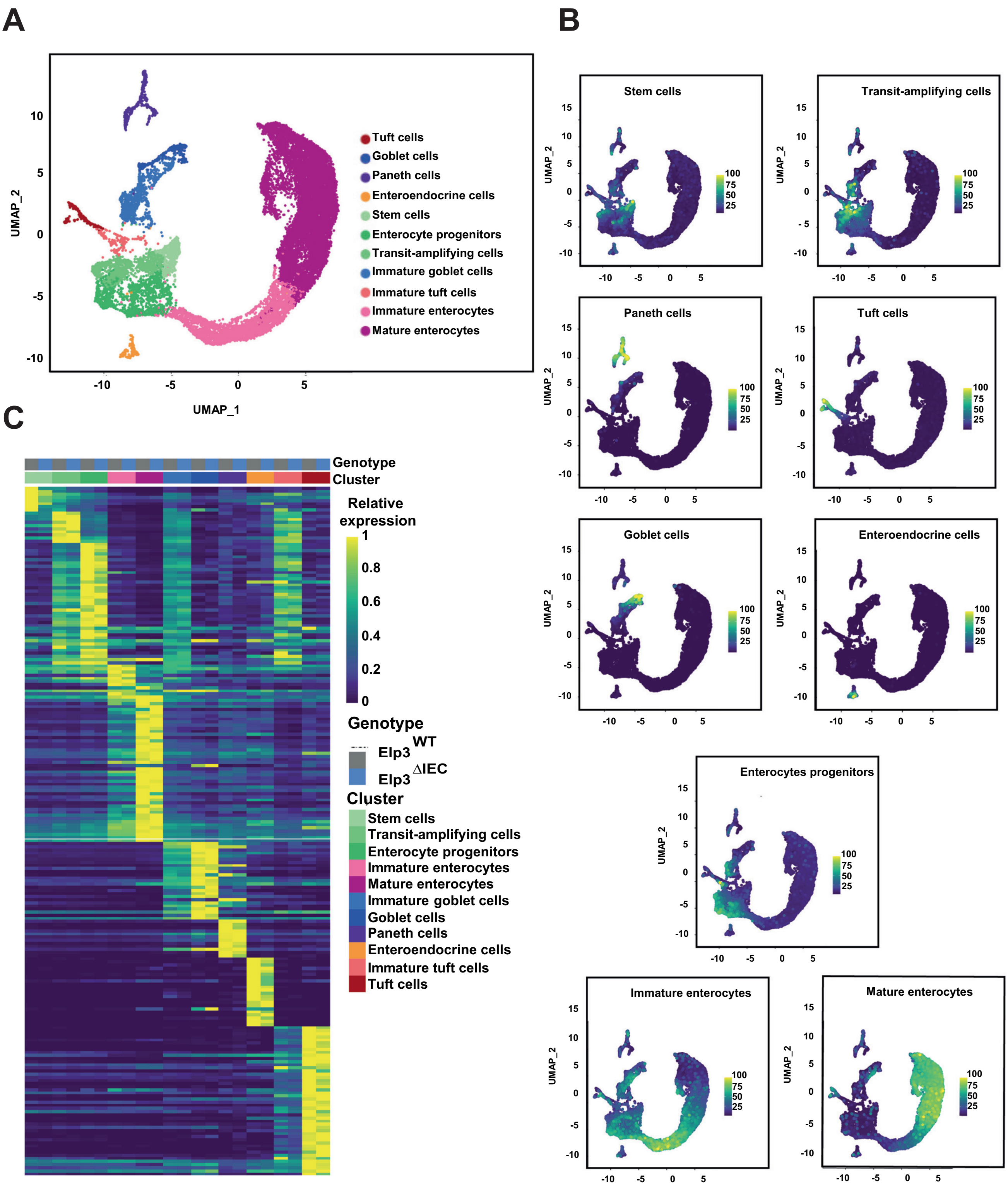


**Figure EV3. Single-cell RNA sequencing analysis of mouse intestinal epithelium.**

(**A**) Cell-type clustering. Two-dimensional graphical representation of the cell-type clustering in the small intestine of both Elp3$^{WT}$ and Elp3$^{\Delta IEC}$ mice after overnight rIL-13 treatment ($n = 4$ pooled mice). (**B**, **C**) Cell-type signatures in mouse intestinal epithelium. UMAP showing expression and distribution of representative genes in clusters from A (**B**). Heatmap of cluster marker genes in the small intestine of both Elp3$^{WT}$ and Elp3$^{\Delta IEC}$ mice after overnight rIL-13 treatment ($n = 2$ pooled mice per genotype) (**C**).

 © The Author(s)

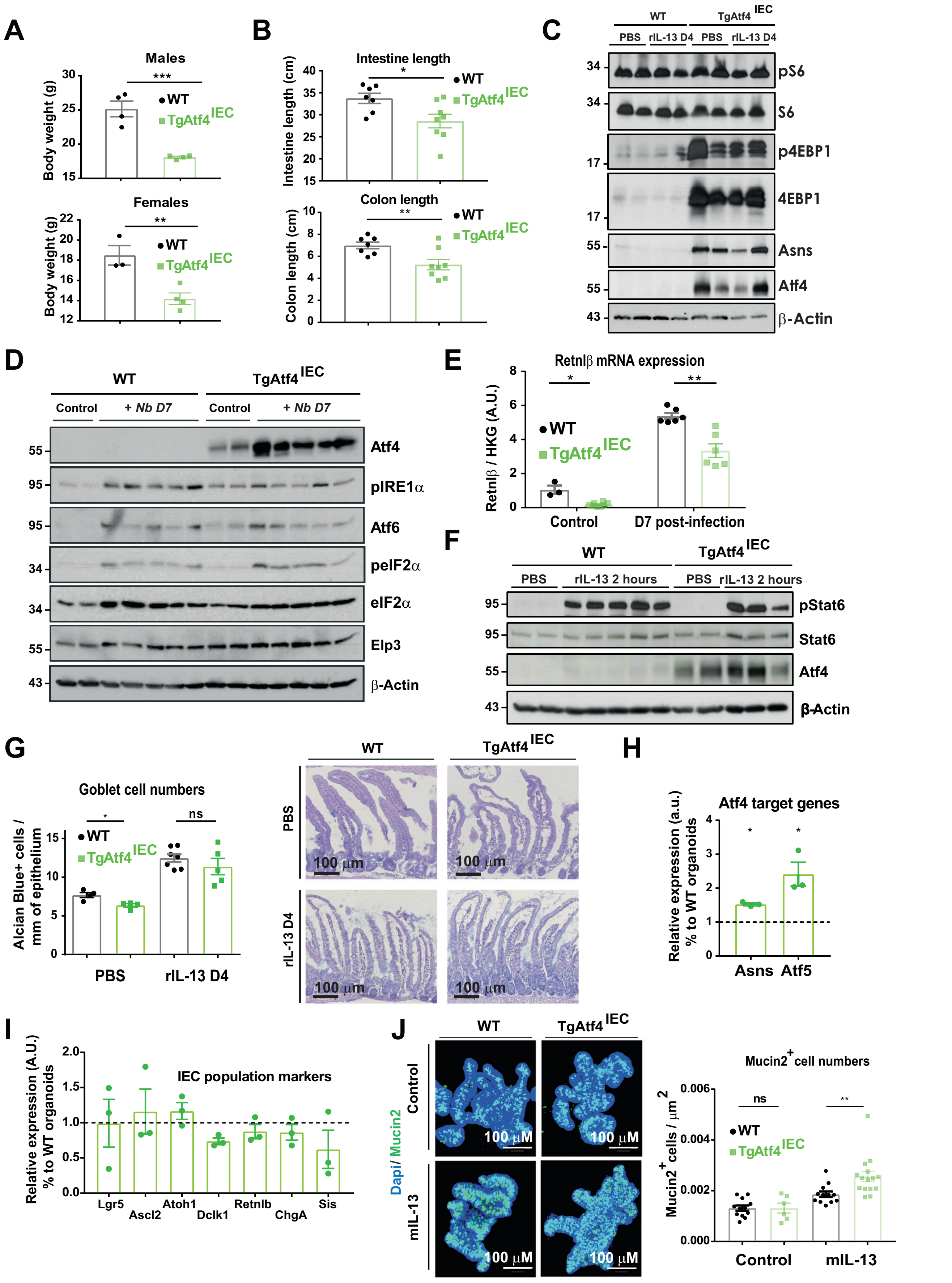
A
Males
Body weight (g)
WT
TgAtf4 IEC
Females
B
Intestine length
Intestine length (cm)
Colon length
Colon length (cm)
C
WT
TgAtf4 IEC
PBS
rIL-13 D4
pS6
S6
p4EBP1
4EBP1
Asns
Atf4
β-Actin
D
Control
+ Nb D7
pIRE1α
Atf6
peIF2α
eIF2α
Elp3
E
Retnlβ mRNA expression
Retnlβ / HKG (A.U.)
Control
D7 post-infection
F
rIL-13 2 hours
pStat6
Stat6
G
Goblet cell numbers
Alcian Blue+ cells / mm of epithelium
ns
100 µm
H
Atf4 target genes
Relative expression (a.u.) % to WT organoids
Asns
Atf5
I
IEC population markers
Relative expression (A.U.) % to WT organoids
Lgr5
Ascl2
Atoh1
Dclk1
Retnlb
ChgA
Sis
J
Dapi/ Mucin2
mIL-13
100 µM
Mucin2+ cell numbers
Mucin2+ cells / µm2

**Figure EV4. Atf4 overexpression in mouse intestinal epithelium inhibits tuft cell differentiation.**

(**A**) Atf4 overexpression in IECs deregulates the body weight. A quantification of 8 weeks old WT and TgAtf4$^{IEC}$ mice body weight (mean values ± SEM; Student *t*-test; $n \geq 3$ for females and $n = 4$ for males; $^{**}p < 0.01$, $^{***}p < 0.001$) is illustrated. (**B**) Atf4 overexpression in IECs shortens the length of both small intestines and colons. A quantification of both intestine and colon lengths in WT ($n = 7$) and TgAtf4 $^{IEC}$ ($n = 8$) mice (mean values ± SEM; Student *t*-test; $^{*}p < 0.05$, $^{**}p < 0.01$) is illustrated. (**C**) Atf4 overexpression in the intestinal epithelium does not change S6 phosphorylation but enhances 4EBP1 protein levels. Extracts from the intestinal epithelium of both WT and TgAtf4$^{IEC}$ mice were subjected to western blot analyses. (**D**) Atf4 overexpression in IECs does not trigger the canonical UPR pathway upon helminth infection. Extracts from both WT and TgAtf4$^{IEC}$ mice naive or infected with *N. brasiliensis* for 7 days were subjected to western blot analyses using the indicated antibodies. Note that the anti-Atf4 blot is identical to the one illustrated in Fig. 10F. (**E**) Defective mRNA induction of Retnlβ upon helminth infection in Atf4-overexpressing IECs. Mice of the indicated genotypes were infected or not with *N. brasiliensis* for 7 days and total RNAs were subjected to real-time PCRs. Retnlβ mRNA levels were quantified and normalization was calculated on the average of the two housekeeping genes Gapdh and 36b4 (mean values ± SEM; Mann–Whitney test; $n \geq 3$; $^{*}p < 0.05$, $^{**}p < 0.01$). (**F**) IL-13-dependent Stat6 phosphorylation does not change upon Atf4 overexpression. WT and TgAtf4$^{IEC}$ mice were treated or not with rIL-13 for 2 hours, and the resulting extracts were subjected to western blot analyses. (**G**) IL-13-dependent goblet cell expansion is similar in intestines from WT and TgAtf4$^{IEC}$ mice. Immunostainings and quantifications (right and left panels, respectively) of goblet cells (Alcian Blue$^{+}$ cells) in intestines from WT and TgAtf4 mice treated or not with rIL-13 for 4 days (mean values ± SEM; Mann–Whitney test; $n \geq 3$ mice; $^{*}p < 0.05$) are illustrated. (**H**, **I**) Atf4 overexpression in ex-vivo organoids induces the expression of Atf4 target genes but does not change levels of epithelial cell markers. mRNA levels of Atf4 target genes (Asns and Atf5) (**H**) and IEC population markers (**I**) in ex-vivo organoids extracted from WT and TgAtf4$^{IEC}$ mice (ratio to WT organoids) were quantified by real-time PCRs. Normalization was calculated on the average of the three housekeeping genes Gapdh, β-Actin and 36b4 (mean values ± SEM; Student *t*-test ; $n = 3$; $^{*}p < 0.05$). (**J**) IL-13-dependent goblet cell expansion is not reduced in ex-vivo organoids from TgAtf4$^{IEC}$ mice. Immunostainings and quantifications (right and left panels, respectively) of goblet cells (Mucin2$^{+}$ cells in green) in ex-vivo organoids of WT and TgAtf4 mice treated or not with mIL-13 (mean values ± SEM; Mann–Whitney test; $n \geq 6$ organoids from $n = 2$ mice; $^{**}p < 0.01$) are illustrated. Source data are available online for this figure.

 © The Author(s)
